# Supplementary figures and images for: Accuracy of rapid point-of-care antigen-based diagnostics for SARS-CoV-2: An updated systematic review and meta-analysis with meta-regression analyzing influencing factors
Source: PLoS Med. 2022 May 26;19(5):e1004011. doi: 10.1371/journal.pmed.1004011 (PMC9187092; doi:10.1371/journal.pmed.1004011)

S2 Fig. Details of QUADAS assessment.

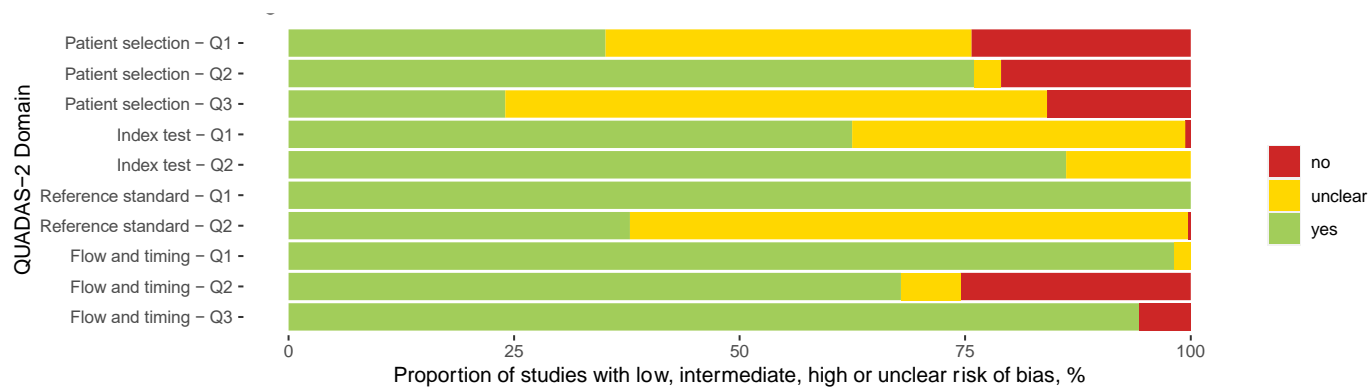

Supplement: S2 Fig — (PDF) [file pmed.1004011.s003.pdf]
