# Supplementary material for: Accuracy of rapid point-of-care antigen-based diagnostics for SARS-CoV-2: An updated systematic review and meta-analysis with meta-regression analyzing influencing factors
Source: PLoS Med. 2022 May 26;19(5):e1004011. doi: 10.1371/journal.pmed.1004011 (PMC9187092; doi:10.1371/journal.pmed.1004011)

S3 Fig. Forest plots for subgroup analysis by Ct-values.

Caption: CI = confidence interval

Fig A – Forest plots for Ct-values lower 20

Author, Study ID	Sample size	Sensitivity [95%CI]
Alemany, a02.ov	258	0.98 [0.96, 1.00]
Gupta, a13.1	5	0.80 [0.28, 1.00]
Gupta, a13.1	27	0.96 [0.81, 1.00]
Krüttgen, a16.1	5	1.00 [0.48, 1.00]
Takeda, a50.1	9	1.00 [0.66, 1.00]
Favresse, f31.1	2	1.00 [0.16, 1.00]
Favresse, f31.1	34	0.97 [0.85, 1.00]
Favresse, f31.2	2	0.50 [0.01, 0.99]
Favresse, f31.2	34	0.94 [0.80, 0.99]
Favresse, f31.3	2	1.00 [0.16, 1.00]
Favresse, f31.3	34	0.97 [0.85, 1.00]
Favresse, f31.4	2	1.00 [0.16, 1.00]
Favresse, f31.4	34	0.97 [0.85, 1.00]
Pena, f36.1	31	0.97 [0.83, 1.00]
Young, f56.1	71	0.83 [0.72, 0.91]
Young, f56.1	62	0.76 [0.63, 0.86]
Faíco−Filho, f63.1	13	1.00 [0.75, 1.00]
Bouassa, f67.1	25	1.00 [0.86, 1.00]
Thell, f81.1	51	1.00 [0.93, 1.00]
Sberna, f83.1	4	0.75 [0.19, 0.99]
Pérez...García, f111.1	41	1.00 [0.91, 1.00]
Pérez...García, f111.2	41	1.00 [0.91, 1.00]
Blairon, f113.1	28	1.00 [0.88, 1.00]
Blairon, f113.2	28	1.00 [0.88, 1.00]
Blairon, f113.3	28	1.00 [0.88, 1.00]
Nordgren, f117.1	47	0.94 [0.82, 0.99]
Nordgren, f117.2	47	0.98 [0.89, 1.00]
Fernandez−Montero, f126.1	9	1.00 [0.66, 1.00]
García−Fiñana, f131.1	11	0.91 [0.59, 1.00]
Lee, f136.1	44	0.89 [0.75, 0.96]
Carbonell−Sahuquillo, f141.1	17	1.00 [0.80, 1.00]
Van Honacker, f143.1	14	1.00 [0.77, 1.00]
Van Honacker, f143.2	14	1.00 [0.77, 1.00]
Van Honacker, f143.3	14	1.00 [0.77, 1.00]
Van Honacker, f143.4	13	1.00 [0.75, 1.00]
Van Honacker, f143.5	14	1.00 [0.77, 1.00]
Van Honacker, f143.6	61	1.00 [0.94, 1.00]
Kurihara, f152.1	16	1.00 [0.79, 1.00]
Kolwijck, f168.1	25	1.00 [0.86, 1.00]
Mayanskiy, f188.1	26	1.00 [0.87, 1.00]
Fourati, f190.1	40	0.90 [0.76, 0.97]
Fourati, f190.2	40	1.00 [0.91, 1.00]
Fourati, f190.3	40	0.95 [0.83, 0.99]
Fourati, f190.4	40	1.00 [0.91, 1.00]
Fourati, f190.5	40	0.80 [0.64, 0.91]
Fourati, f190.6	40	0.98 [0.87, 1.00]
Suzuki, f194.1	28	0.96 [0.82, 1.00]
Suzuki, f194.2	28	1.00 [0.88, 1.00]
Thirion−Romero, f195.1	75	0.88 [0.78, 0.94]

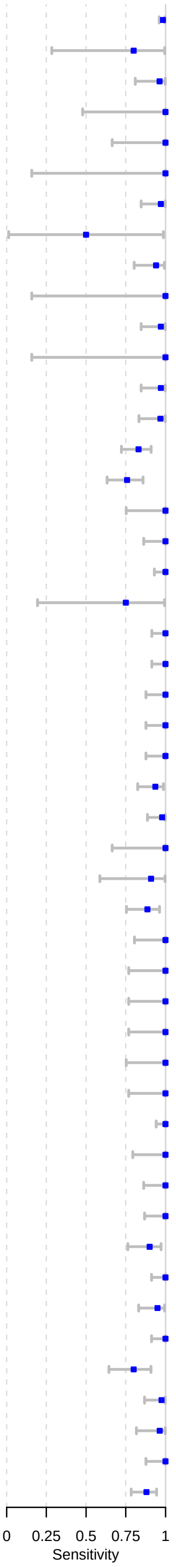

Fig B – Forest plots for Ct-values greater 20

Author, Study ID	Sample size	Sensitivity [95%CI]
Alemany, a02.ov	305	0.99 [0.98, 1.00]
Gupta, a13.1	28	0.86 [0.67, 0.96]
Krüttgen, a16.1	12	1.00 [0.74, 1.00]
Takeda, a50.1	23	1.00 [0.85, 1.00]
Favresse, f31.1	22	0.86 [0.65, 0.97]
Favresse, f31.2	22	0.96 [0.77, 1.00]
Favresse, f31.3	22	0.96 [0.77, 1.00]
Favresse, f31.4	22	0.96 [0.77, 1.00]
Young, f56.1	54	0.43 [0.29, 0.57]
Bouassa, f67.1	52	0.94 [0.84, 0.99]
Sberna, f83.1	14	0.07 [0.00, 0.34]
Pérez...García, f111.1	43	0.93 [0.81, 0.98]
Pérez...García, f111.2	43	0.95 [0.84, 0.99]
Nordgren, f117.1	66	0.85 [0.74, 0.92]
Nordgren, f117.2	66	0.91 [0.81, 0.97]
Fernandez−Montero, f126.1	23	0.91 [0.72, 0.99]
Lee, f136.1	65	0.55 [0.42, 0.68]
Van Honacker, f143.1	18	1.00 [0.81, 1.00]
Van Honacker, f143.2	18	0.89 [0.65, 0.99]
Van Honacker, f143.3	18	1.00 [0.81, 1.00]
Van Honacker, f143.4	18	0.94 [0.73, 1.00]
Van Honacker, f143.5	18	1.00 [0.81, 1.00]
Van Honacker, f143.6	108	0.93 [0.86, 0.97]
Kurihara, f152.1	29	0.97 [0.82, 1.00]
Mayanskiy, f188.1	62	0.98 [0.91, 1.00]
Fourati, f190.1	90	0.63 [0.52, 0.73]
Fourati, f190.2	90	0.90 [0.82, 0.95]
Fourati, f190.3	90	0.83 [0.74, 0.90]
Fourati, f190.4	90	0.94 [0.88, 0.98]
Fourati, f190.5	90	0.54 [0.44, 0.65]
Fourati, f190.6	90	0.92 [0.85, 0.97]
Suzuki, f194.1	32	0.81 [0.64, 0.93]
Suzuki, f194.2	32	0.84 [0.67, 0.95]
Thirion−Romero, f195.1	92	0.73 [0.63, 0.82]

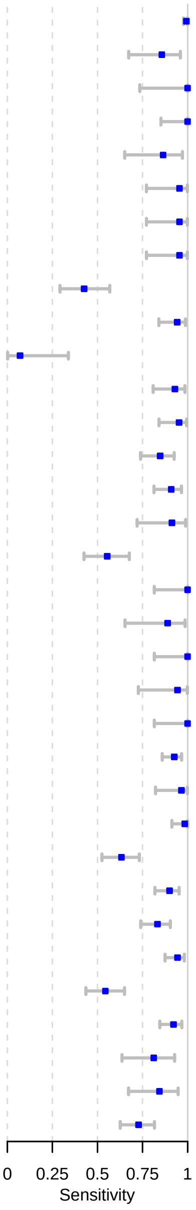

Fig C – Forest plots for Ct-values lower 25

Author, Study ID	Sample size	Sensitivity [95%CI]
Alemany, a02.ov	305	0.99 [0.98, 1.00]
Bulilete, a07.1	95	0.90 [0.81, 0.95]
Gupta, a13.1	28	0.86 [0.67, 0.96]
Krüttgen, a16.1	12	1.00 [0.74, 1.00]
Krüger, a17.1	18	1.00 [0.81, 1.00]
Porte, a31.1	52	1.00 [0.93, 1.00]
Porte, a32.1	27	1.00 [0.87, 1.00]
Porte, a32.2	27	1.00 [0.87, 1.00]
Scohy, a34.1	10	1.00 [0.69, 1.00]
Weitzel, a41.1	53	0.85 [0.72, 0.93]
Weitzel, a41.3	52	0.21 [0.11, 0.35]
Weitzel, a41.4	54	1.00 [0.93, 1.00]
Takeda, a50.1	23	1.00 [0.85, 1.00]
Krüger, a52.ov	72	0.96 [0.88, 0.99]
Agullo, a56.2	68	0.75 [0.63, 0.85]
Agullo, a56.3	63	0.38 [0.26, 0.51]
Strömer, f11.1	32	1.00 [0.89, 1.00]
Pollock, f17.1	150	0.99 [0.96, 1.00]
Favresse, f31.1	22	0.86 [0.65, 0.97]
Favresse, f31.2	22	0.96 [0.77, 1.00]
Favresse, f31.3	22	0.96 [0.77, 1.00]
Favresse, f31.4	22	0.96 [0.77, 1.00]
Pena, f36.1	30	0.67 [0.47, 0.83]
Jääskeläinen, f50.1	89	0.99 [0.94, 1.00]
Jääskeläinen, f50.2	97	0.99 [0.94, 1.00]
Jääskeläinen, f50.3	92	0.98 [0.92, 1.00]
Pérez−García, f52.1	84	0.94 [0.87, 0.98]
Pérez−García, f52.2	84	0.96 [0.90, 0.99]
Young, f56.1	54	0.43 [0.29, 0.57]
Krüger, f58.1	95	0.93 [0.85, 0.97]
Pollock, f59.1	95	0.96 [0.90, 0.99]
Pollock, f59.1	12	1.00 [0.74, 1.00]
Faíco−Filho, f63.1	37	0.97 [0.86, 1.00]
Pickering, f73.1	69	0.99 [0.92, 1.00]
Pickering, f73.3	69	0.91 [0.82, 0.97]
Pickering, f73.4	69	0.96 [0.88, 0.99]
Pickering, f73.5	69	0.94 [0.86, 0.98]
Pickering, f73.6	69	0.88 [0.78, 0.95]
Pickering, f73.7	82	0.84 [0.74, 0.91]
Pickering, f73.8	66	0.97 [0.90, 1.00]
Pickering, f73.9	52	0.96 [0.87, 1.00]
Thell, f81.1	125	0.98 [0.94, 1.00]
Sberna, f83.1	14	0.07 [0.00, 0.34]
Thakur, f88.1	60	0.45 [0.32, 0.58]
Pérez...García, f111.1	43	0.93 [0.81, 0.98]
Pérez...García, f111.2	43	0.95 [0.84, 0.99]
Bornemann, f112.1	43	0.95 [0.84, 0.99]
Blairon, f113.1	69	0.96 [0.88, 0.99]
Blairon, f113.2	69	0.97 [0.90, 1.00]
Blairon, f113.3	69	0.97 [0.90, 1.00]
Nordgren, f117.1	66	0.85 [0.74, 0.92]
Nordgren, f117.2	66	0.91 [0.81, 0.97]
Leixner, f125.1	33	1.00 [0.89, 1.00]
Fernandez−Montero, f126.1	23	0.91 [0.72, 0.99]
Terpos, f130.1	70	1.00 [0.95, 1.00]
García−Fiñana, f131.1	25	0.60 [0.39, 0.79]
Lee, f136.1	65	0.55 [0.42, 0.68]
Carbonell−Sahuquillo, f141.1	25	0.96 [0.80, 1.00]
Van Honacker, f143.1	18	1.00 [0.81, 1.00]
Van Honacker, f143.2	18	0.89 [0.65, 0.99]
Van Honacker, f143.3	18	1.00 [0.81, 1.00]
Van Honacker, f143.4	18	0.94 [0.73, 1.00]
Van Honacker, f143.5	18	1.00 [0.81, 1.00]
Van Honacker, f143.6	108	0.93 [0.86, 0.97]
Kurihara, f152.1	29	0.97 [0.82, 1.00]
Baccani, f165.2	5	1.00 [0.48, 1.00]
Baccani, f165.3	5	1.00 [0.48, 1.00]
Kolwijck, f168.1	34	1.00 [0.90, 1.00]
FINDdx, f175.1	48	1.00 [0.93, 1.00]
FINDdx, f176.1	29	1.00 [0.88, 1.00]
FINDdx, f177.1	64	0.70 [0.58, 0.81]
FINDdx, f177.2	33	0.88 [0.72, 0.97]
FINDdx, f178.1	89	0.96 [0.89, 0.99]
FINDdx, f179.1	37	0.92 [0.78, 0.98]
FINDdx, f180.1	80	0.98 [0.91, 1.00]
FINDdx, f180.2	81	0.88 [0.78, 0.94]
FINDdx, f181.1	47	0.89 [0.77, 0.96]
FINDdx, f181.2	52	0.96 [0.87, 1.00]
FINDdx, f182.1	46	1.00 [0.92, 1.00]
FINDdx, f182.2	46	1.00 [0.92, 1.00]
Kim, f186.1	15	1.00 [0.78, 1.00]
Kim, f186.2	54	0.91 [0.80, 0.97]
Mayanskiy, f188.1	62	0.98 [0.91, 1.00]
Fourati, f190.1	90	0.63 [0.52, 0.73]
Fourati, f190.1	130	0.71 [0.62, 0.78]
Fourati, f190.2	90	0.90 [0.82, 0.95]
Fourati, f190.2	130	0.93 [0.87, 0.97]
Fourati, f190.3	90	0.83 [0.74, 0.90]
Fourati, f190.3	130	0.87 [0.80, 0.92]
Fourati, f190.4	90	0.94 [0.88, 0.98]
Fourati, f190.4	130	0.96 [0.91, 0.99]
Fourati, f190.5	90	0.54 [0.44, 0.65]
Fourati, f190.5	130	0.62 [0.53, 0.71]
Fourati, f190.6	90	0.92 [0.85, 0.97]
Fourati, f190.6	130	0.94 [0.88, 0.97]
Orsi, f191.1	23	1.00 [0.85, 1.00]
Orsi, f191.2	23	1.00 [0.85, 1.00]
Thirion−Romero, f195.1	194	0.82 [0.76, 0.87]
Thirion−Romero, f195.1	92	0.73 [0.63, 0.82]

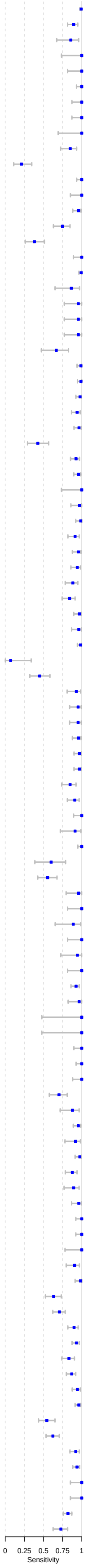

Fig D – Forest plots for Ct-values greater 25

Author, Study ID	Sample size	Sensitivity [95%CI]
Alemany, a02.ov	285	0.90 [0.86, 0.93]
Bulilete, a07.1	25	0.40 [0.21, 0.61]
Gupta, a13.1	13	0.62 [0.32, 0.86]
Krüger, a17.1	29	0.62 [0.42, 0.79]
Porte, a31.1	18	0.72 [0.47, 0.90]
Weitzel, a41.1	26	0.15 [0.04, 0.35]
Weitzel, a41.3	26	0.08 [0.01, 0.25]
Weitzel, a41.4	26	0.54 [0.33, 0.73]
Takeda, a50.1	18	0.89 [0.65, 0.99]
Krüger, a52.ov	33	0.67 [0.48, 0.82]
Favresse, f31.1	19	0.32 [0.13, 0.57]
Favresse, f31.2	19	0.53 [0.29, 0.76]
Favresse, f31.3	19	0.68 [0.43, 0.87]
Favresse, f31.4	19	0.53 [0.29, 0.76]
Jääskeläinen, f50.1	34	0.82 [0.66, 0.93]
Jääskeläinen, f50.2	35	0.69 [0.51, 0.83]
Jääskeläinen, f50.3	34	0.76 [0.59, 0.89]
Pérez−García, f52.1	86	0.14 [0.07, 0.23]
Pérez−García, f52.2	86	0.24 [0.16, 0.35]
Young, f56.1	23	0.17 [0.05, 0.39]
Krüger, f58.1	51	0.63 [0.48, 0.76]
Sberna, f83.1	23	0.04 [0.00, 0.22]
Thakur, f88.1	21	0.10 [0.01, 0.30]
Pérez...García, f111.1	46	0.41 [0.27, 0.57]
Pérez...García, f111.2	46	0.52 [0.37, 0.67]
Nordgren, f117.1	20	0.45 [0.23, 0.69]
Nordgren, f117.2	20	0.55 [0.32, 0.77]
Fernandez−Montero, f126.1	9	0.44 [0.14, 0.79]
García−Fiñana, f131.1	17	0.18 [0.04, 0.43]
Lee, f136.1	118	0.17 [0.11, 0.25]
Van Honacker, f143.1	18	0.89 [0.65, 0.99]
Van Honacker, f143.2	18	0.44 [0.22, 0.69]
Van Honacker, f143.3	18	0.72 [0.47, 0.90]
Van Honacker, f143.4	18	0.72 [0.47, 0.90]
Van Honacker, f143.5	18	0.72 [0.47, 0.90]
Van Honacker, f143.6	64	0.55 [0.42, 0.67]
Kurihara, f152.1	13	1.00 [0.75, 1.00]
Baccani, f165.2	9	0.56 [0.21, 0.86]
Baccani, f165.3	9	0.44 [0.14, 0.79]
Kim, f186.1	12	0.92 [0.62, 1.00]
Kim, f186.2	45	0.98 [0.88, 1.00]
Mayanskiy, f188.1	42	0.93 [0.80, 0.98]
Fourati, f190.1	74	0.15 [0.08, 0.25]
Fourati, f190.2	74	0.65 [0.53, 0.76]
Fourati, f190.3	74	0.58 [0.46, 0.70]
Fourati, f190.4	74	0.66 [0.54, 0.77]
Fourati, f190.5	74	0.19 [0.11, 0.30]
Fourati, f190.6	74	0.64 [0.52, 0.74]
Orsi, f191.1	32	0.97 [0.84, 1.00]
Orsi, f191.2	32	0.91 [0.75, 0.98]
Thirion−Romero, f195.1	88	0.33 [0.23, 0.44]
Thirion−Romero, f195.1	195	0.25 [0.19, 0.31]

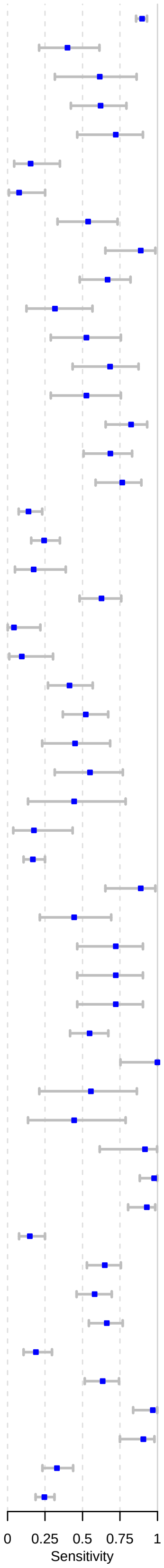

Fig E – Forest plots for Ct-values lower 30

Author, Study ID	Sample size	Sensitivity [95%CI]
Alemany, a02.ov	285	0.90 [0.86, 0.93]
Bulilete, a07.1	25	0.40 [0.21, 0.61]
Gremmels, a12.1	106	0.95 [0.89, 0.98]
Gremmels, a12.2	49	0.98 [0.89, 1.00]
Gupta, a13.1	13	0.62 [0.32, 0.86]
Merino−Amador, a25.1	254	0.94 [0.91, 0.97]
Nalumansi, a27.1	37	0.92 [0.78, 0.98]
Pilarowski, a29.1	15	0.93 [0.68, 1.00]
Pilarowski, a29.1	16	0.94 [0.70, 1.00]
Takeda, a50.1	18	0.89 [0.65, 0.99]
Krüger, a52.ov	93	0.94 [0.86, 0.98]
Agullo, a56.2	96	0.58 [0.48, 0.68]
Agullo, a56.3	87	0.32 [0.23, 0.43]
Strömer, f11.1	108	0.73 [0.64, 0.81]
Pollock, f17.1	214	0.96 [0.92, 0.98]
Favresse, f31.1	19	0.32 [0.13, 0.57]
Favresse, f31.2	19	0.53 [0.29, 0.76]
Favresse, f31.3	19	0.68 [0.43, 0.87]
Favresse, f31.4	19	0.53 [0.29, 0.76]
Jääskeläinen, f50.1	34	0.82 [0.66, 0.93]
Jääskeläinen, f50.1	123	0.94 [0.89, 0.98]
Jääskeläinen, f50.2	35	0.69 [0.51, 0.83]
Jääskeläinen, f50.2	132	0.91 [0.85, 0.95]
Jääskeläinen, f50.3	34	0.76 [0.59, 0.89]
Jääskeläinen, f50.3	126	0.92 [0.86, 0.96]
Okoye, f51.1	36	0.67 [0.49, 0.81]
Okoye, f51.1	42	0.57 [0.41, 0.72]
Young, f56.1	23	0.17 [0.05, 0.39]
Krüger, f58.1	122	0.90 [0.83, 0.95]
Pollock, f59.1	133	0.79 [0.71, 0.86]
Pollock, f59.1	29	0.83 [0.64, 0.94]
Faíco−Filho, f63.1	59	0.88 [0.77, 0.95]
Thell, f81.1	173	0.92 [0.88, 0.96]
Sberna, f83.1	23	0.04 [0.00, 0.22]
Thakur, f88.1	21	0.10 [0.01, 0.30]
Holzner, f97.1	456	0.80 [0.76, 0.84]
Holzner, f97.1	423	0.80 [0.76, 0.83]
Pérez...García, f111.1	46	0.41 [0.27, 0.57]
Pérez...García, f111.2	46	0.52 [0.37, 0.67]
Blairon, f113.1	97	0.86 [0.77, 0.92]
Blairon, f113.2	97	0.86 [0.77, 0.92]
Blairon, f113.3	97	0.87 [0.78, 0.93]
Nordgren, f117.1	20	0.45 [0.23, 0.69]
Nordgren, f117.2	20	0.55 [0.32, 0.77]
Leixner, f125.1	61	0.92 [0.82, 0.97]
Fernandez−Montero, f126.1	9	0.44 [0.14, 0.79]
Terpos, f130.1	100	0.99 [0.95, 1.00]
García−Fiñana, f131.1	17	0.18 [0.04, 0.43]
Lee, f136.1	118	0.17 [0.11, 0.25]
Seynaeve, f137.1	45	0.91 [0.79, 0.98]
Seynaeve, f137.2	45	0.69 [0.53, 0.82]
Carbonell−Sahuquillo, f141.1	29	0.83 [0.64, 0.94]
Van Honacker, f143.1	18	0.89 [0.65, 0.99]
Van Honacker, f143.2	18	0.44 [0.22, 0.69]
Van Honacker, f143.3	18	0.72 [0.47, 0.90]
Van Honacker, f143.4	18	0.72 [0.47, 0.90]
Van Honacker, f143.5	18	0.72 [0.47, 0.90]
Van Honacker, f143.6	64	0.55 [0.42, 0.67]
Kurihara, f152.1	13	1.00 [0.75, 1.00]
Kim, f155.1	54	0.94 [0.85, 0.99]
Tinker, f156.1	9	0.89 [0.52, 1.00]
Baccani, f165.2	9	0.56 [0.21, 0.86]
Baccani, f165.3	9	0.44 [0.14, 0.79]
Kahn, f167.1	81	0.64 [0.53, 0.75]
Kolwijck, f168.1	41	0.95 [0.84, 0.99]
Jegerlehner, f172.1	116	0.79 [0.71, 0.86]
Jegerlehner, f172.1	109	0.84 [0.76, 0.91]
FINDdx, f177.1	86	0.58 [0.47, 0.69]
FINDdx, f177.2	51	0.76 [0.62, 0.87]
FINDdx, f178.1	105	0.90 [0.82, 0.95]
FINDdx, f179.1	93	0.74 [0.64, 0.83]
FINDdx, f180.1	96	0.94 [0.87, 0.98]
FINDdx, f180.2	103	0.75 [0.65, 0.83]
FINDdx, f181.1	87	0.66 [0.55, 0.75]
FINDdx, f181.2	84	0.83 [0.74, 0.91]
FINDdx, f182.1	72	0.92 [0.83, 0.97]
FINDdx, f182.2	72	0.92 [0.83, 0.97]
Kim, f186.1	12	0.92 [0.62, 1.00]
Kim, f186.2	45	0.98 [0.88, 1.00]
Mayanskiy, f188.1	42	0.93 [0.80, 0.98]
Fourati, f190.1	74	0.15 [0.08, 0.25]
Fourati, f190.2	74	0.65 [0.53, 0.76]
Fourati, f190.3	74	0.58 [0.46, 0.70]
Fourati, f190.4	74	0.66 [0.54, 0.77]
Fourati, f190.5	74	0.19 [0.11, 0.30]
Fourati, f190.6	74	0.64 [0.52, 0.74]
Orsi, f191.1	32	0.97 [0.84, 1.00]
Orsi, f191.2	32	0.91 [0.75, 0.98]
Suzuki, f194.1	32	0.81 [0.64, 0.93]
Suzuki, f194.2	32	0.84 [0.67, 0.95]
Thirion−Romero, f195.1	88	0.33 [0.23, 0.44]
Cento, f196.1	139	0.91 [0.85, 0.96]

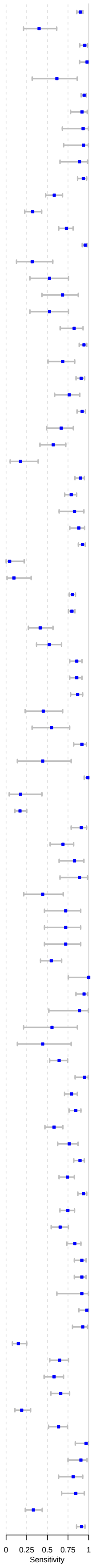

Fig F – Forest plots for Ct-values greater 30

Author, Study ID	Sample size	Sensitivity [95%CI]
Alemany, a02.ov	103	0.57 [0.47, 0.67]
Bulilete, a07.1	14	0.14 [0.02, 0.43]
Gupta, a13.1	4	0.25 [0.01, 0.81]
Merino−Amador, a25.1	41	0.51 [0.35, 0.67]
Nalumansi, a27.1	44	0.54 [0.39, 0.70]
Nalumansi, a27.1	9	0.56 [0.21, 0.86]
Takeda, a50.1	12	0.17 [0.02, 0.48]
Krüger, a52.ov	12	0.33 [0.10, 0.65]
Favresse, f31.1	16	0.19 [0.04, 0.46]
Favresse, f31.1	3	0.33 [0.01, 0.91]
Favresse, f31.2	3	0.00 [0.00, 0.71]
Favresse, f31.2	16	0.06 [0.00, 0.30]
Favresse, f31.3	3	0.67 [0.09, 0.99]
Favresse, f31.3	16	0.19 [0.04, 0.46]
Favresse, f31.4	3	0.00 [0.00, 0.71]
Favresse, f31.4	16	0.06 [0.00, 0.30]
Pena, f36.1	12	0.08 [0.00, 0.38]
Jääskeläinen, f50.1	25	0.12 [0.03, 0.31]
Jääskeläinen, f50.2	26	0.31 [0.14, 0.52]
Jääskeläinen, f50.3	26	0.38 [0.20, 0.59]
Okoye, f51.1	9	0.00 [0.00, 0.34]
Okoye, f51.1	3	0.00 [0.00, 0.71]
Young, f56.1	3	0.00 [0.00, 0.71]
Krüger, f58.1	24	0.42 [0.22, 0.63]
Bouassa, f67.1	23	0.70 [0.47, 0.87]
Sberna, f83.1	21	0.00 [0.00, 0.16]
Thakur, f88.1	3	0.00 [0.00, 0.71]
Pérez...García, f111.1	40	0.05 [0.01, 0.17]
Pérez...García, f111.2	40	0.17 [0.07, 0.33]
Nordgren, f117.1	23	0.13 [0.03, 0.34]
Nordgren, f117.2	23	0.30 [0.13, 0.53]
Fernandez−Montero, f126.1	8	0.12 [0.00, 0.53]
García−Fiñana, f131.1	17	0.00 [0.00, 0.20]
Lee, f136.1	136	0.03 [0.01, 0.07]
Van Honacker, f143.1	8	0.50 [0.16, 0.84]
Van Honacker, f143.2	8	0.12 [0.00, 0.53]
Van Honacker, f143.3	8	0.38 [0.09, 0.76]
Van Honacker, f143.4	8	0.25 [0.03, 0.65]
Van Honacker, f143.5	8	0.38 [0.09, 0.76]
Van Honacker, f143.6	84	0.05 [0.01, 0.12]
Kurihara, f152.1	25	0.20 [0.07, 0.41]
Kim, f155.1	11	0.64 [0.31, 0.89]
Baccani, f165.2	14	0.00 [0.00, 0.23]
Baccani, f165.3	14	0.00 [0.00, 0.23]
Mayanskiy, f188.1	34	0.85 [0.69, 0.95]
Fourati, f190.1	89	0.01 [0.00, 0.06]
Fourati, f190.2	89	0.11 [0.06, 0.20]
Fourati, f190.3	89	0.08 [0.03, 0.16]
Fourati, f190.4	89	0.09 [0.04, 0.17]
Fourati, f190.5	89	0.01 [0.00, 0.06]
Fourati, f190.6	89	0.09 [0.04, 0.17]
Orsi, f191.1	5	0.40 [0.05, 0.85]
Orsi, f191.2	5	0.00 [0.00, 0.52]
Suzuki, f194.1	14	0.00 [0.00, 0.23]
Suzuki, f194.2	14	0.21 [0.05, 0.51]
Thirion−Romero, f195.1	69	0.20 [0.12, 0.32]
Cento, f196.1	59	0.34 [0.22, 0.47]

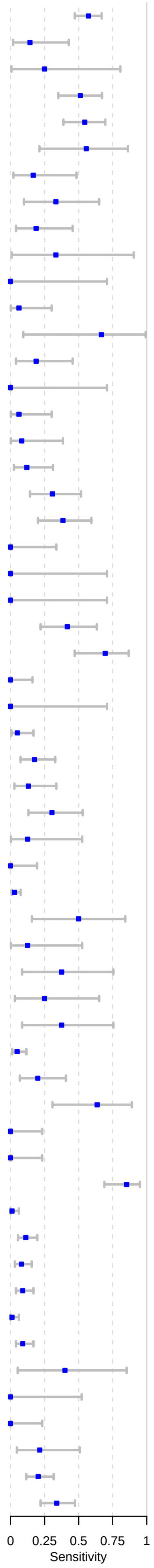

Supplement: S3 Fig — CI, confidence interval; Ct, cycle threshold. (PDF) [file pmed.1004011.s004.pdf]
