# Supplementary material for: Accuracy of rapid point-of-care antigen-based diagnostics for SARS-CoV-2: An updated systematic review and meta-analysis with meta-regression analyzing influencing factors
Source: PLoS Med. 2022 May 26;19(5):e1004011. doi: 10.1371/journal.pmed.1004011 (PMC9187092; doi:10.1371/journal.pmed.1004011)

S4 Fig. Forest plots for subgroup analysis by CT-values per test.

Caption: CI = confidence interval

Fig A – Forest plots for Ct-value lower 20

Test assessed	N datasets	Total Sample Size	Pooled Sensitivity (95% CI)
Panbio	14	616	97.2% (95.3–99.2)
Standard Q	15	833	98.1% (96.3–99.9)

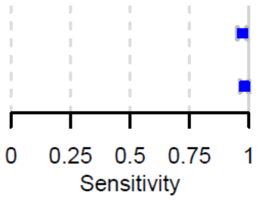

Fig B – Forest plots for Ct-value greater 20

Test assessed	N datasets	Total Sample Size	Pooled Sensitivity (95% CI)
Panbio	7	636	89.2% (82.1–96.3)
Standard Q	10	411	89% (81–96.9)

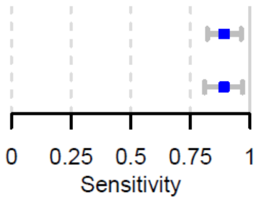

Fig C – Forest plots for Ct-value lower 25

Test assessed	N datasets	Total Sample Size	Pooled Sensitivity (95% CI)
Coris	4	233	76% (58–94)
Innova	4	214	75.5% (48–100)
Panbio	27	5769	89.8% (85.4–94.3)
Standard F	7	305	95.5% (91.3–99.8)
Standard Q	21	2309	92.6% (88.5–96.7)

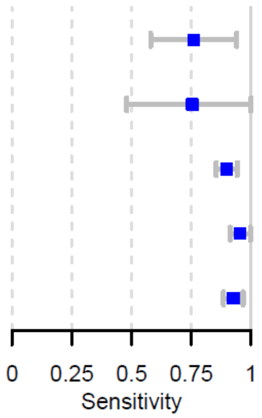

Fig D – Forest plots for Ct-value greater 25

Test assessed	N datasets	Total Sample Size	Pooled Sensitivity (95% CI)
Panbio	13	969	51.2% (39.4–63)
Standard Q	11	413	56.4% (45.1–67.8)

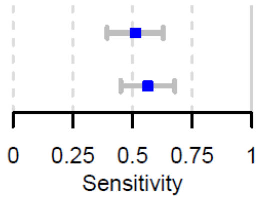

Fig E – Forest plots for Ct-value lower 30

Test assessed	N datasets	Total Sample Size	Pooled Sensitivity (95% CI)
BinaxNow	6	332	83% (69.5–96.5)
LumiraDx	6	358	92.6% (86.8–98.5)
Panbio	31	5993	73.7% (66–81.3)
Standard F	8	475	77.1% (65.8–88.3)
Standard Q	25	5477	75.7% (67.9–83.4)

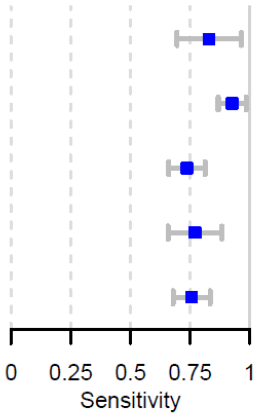

Fig F – Forest plots for Ct-value greater 30

Test assessed	N datasets	Total Sample Size	Pooled Sensitivity (95% CI)
LumiraDx	4	83	36% (26.1–45.9)
Panbio	13	444	22.8% (12.2–33.4)
Standard Q	15	499	20.4% (10.5–30.3)

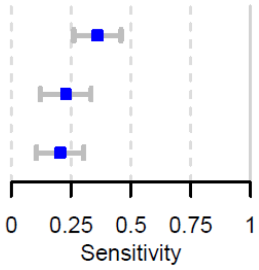

Supplement: S4 Fig — CI, confidence interval; Ct, cycle threshold. (PDF) [file pmed.1004011.s005.pdf]
