# Supplementary material for: Accuracy of rapid point-of-care antigen-based diagnostics for SARS-CoV-2: An updated systematic review and meta-analysis with meta-regression analyzing influencing factors
Source: PLoS Med. 2022 May 26;19(5):e1004011. doi: 10.1371/journal.pmed.1004011 (PMC9187092; doi:10.1371/journal.pmed.1004011)

<u>S5 Fig. Forest plots for subgroup analysis by IFU vs. non-IFU.</u>

Caption: TP = true positive; FP = false positive; FN = false negative; TN = true negative; CI = confidence interval

Fig A - Forest plots for IFU conforming studies

Author, Study ID	Sample size	TP	FN	TN	FP	Sensitivity [95%CI]	Specificity [95%CI]
Albert, a03.1	412	43	11	358	0	0.80 [0.66, 0.89]	1.00 [0.99, 1.00]
Beck, a04.1	346	47	14	284	1	0.77 [0.64, 0.87]	1.00 [0.98, 1.00]
Berger, a05.1	535	106	18	411	0	0.86 [0.78, 0.91]	1.00 [0.99, 1.00]
Berger, a05.2	529	170	21	337	1	0.89 [0.84, 0.93]	1.00 [0.98, 1.00]
Bulilete, a07.1	1362	100	40	1220	2	0.71 [0.63, 0.79]	1.00 [0.99, 1.00]
Drevinek, a10.1	591	148	75	368	0	0.66 [0.60, 0.72]	1.00 [0.99, 1.00]
Drevinek, a10.2	591	139	84	366	2	0.62 [0.56, 0.69]	1.00 [0.98, 1.00]
Fenollar. F., a11.1	341	154	50	130	7	0.76 [0.69, 0.81]	0.95 [0.90, 0.98]
Gremmels, a12.1	1367	101	38	1228	0	0.73 [0.64, 0.80]	1.00 [1.00, 1.00]
Gremmels, a12.2	208	51	12	145	0	0.81 [0.69, 0.90]	1.00 [0.98, 1.00]
Gupta, a13.1	330	63	14	252	1	0.82 [0.71, 0.90]	1.00 [0.98, 1.00]
Igl...i, a15.1	970	158	28	780	4	0.85 [0.79, 0.90]	1.00 [0.99, 1.00]
Lindner, a21.2	289	31	8	249	1	0.80 [0.64, 0.91]	1.00 [0.98, 1.00]
Merino−Amador, a25.1	958	325	34	592	7	0.90 [0.87, 0.93]	0.99 [0.98, 1.00]
Nalumansi, a27.1	262	63	27	159	13	0.70 [0.59, 0.79]	0.92 [0.87, 0.96]
Pilarowski, a29.1	878	15	11	852	0	0.58 [0.37, 0.77]	1.00 [1.00, 1.00]
Schwob, a35.2	271	105	17	149	0	0.86 [0.79, 0.92]	1.00 [0.98, 1.00]
Schwob, a35.1	333	104	8	221	0	0.93 [0.86, 0.97]	1.00 [0.98, 1.00]
Schwob, a35.3	324	116	22	186	0	0.84 [0.77, 0.90]	1.00 [0.98, 1.00]
Shrestha, a36.1	113	40	7	66	0	0.85 [0.72, 0.94]	1.00 [0.95, 1.00]
Torres, a37.1	634	38	41	555	0	0.48 [0.37, 0.60]	1.00 [0.99, 1.00]
Krüger, a52.1	1034	91	13	929	1	0.88 [0.80, 0.93]	1.00 [0.99, 1.00]
Lindner, a53.2	179	33	8	136	2	0.80 [0.65, 0.91]	0.99 [0.95, 1.00]
Lindner, a53.1	179	30	11	137	1	0.73 [0.57, 0.86]	0.99 [0.96, 1.00]
Agullo, a56.1	652	76	56	519	1	0.58 [0.49, 0.66]	1.00 [0.99, 1.00]
Parada−Ricart, a58.1	172	19	7	125	21	0.73 [0.52, 0.88]	0.86 [0.79, 0.91]
FINDdx, a61.1	400	91	11	290	8	0.89 [0.81, 0.94]	0.97 [0.95, 0.99]
FINDdx, a62.1	476	87	30	355	4	0.74 [0.66, 0.82]	0.99 [0.97, 1.00]
FINDdx, a62.2	1239	13	12	1214	0	0.52 [0.31, 0.72]	1.00 [1.00, 1.00]
FINDdx, a63.1	453	93	27	326	7	0.78 [0.69, 0.85]	0.98 [0.96, 0.99]
FINDdx, a63.2	676	27	12	617	20	0.69 [0.52, 0.83]	0.97 [0.95, 0.98]
FINDdx, a64.1	400	94	12	287	7	0.89 [0.81, 0.94]	0.98 [0.95, 0.99]
Takeuchi, f12.1	1186	91	14	1081	0	0.87 [0.79, 0.92]	1.00 [1.00, 1.00]
Lindner, f15.2	144	33	7	104	0	0.82 [0.67, 0.93]	1.00 [0.96, 1.00]
Lindner, f15.1	146	34	6	105	1	0.85 [0.70, 0.94]	0.99 [0.95, 1.00]
Pollock, f17.1	2308	226	66	2004	12	0.77 [0.72, 0.82]	0.99 [0.99, 1.00]
Möckel, f19.1	271	67	22	182	0	0.75 [0.65, 0.84]	1.00 [0.98, 1.00]
Möckel, f19.2	202	18	7	176	1	0.72 [0.51, 0.88]	0.99 [0.97, 1.00]
James, f23.1	2339	86	66	2184	3	0.57 [0.48, 0.65]	1.00 [1.00, 1.00]
Houston, f25.1	728	242	38	426	22	0.86 [0.82, 0.90]	0.95 [0.93, 0.97]
Torres, f29.1	178	73	18	87	0	0.80 [0.71, 0.88]	1.00 [0.96, 1.00]
Torres, f29.2	92	15	10	67	0	0.60 [0.39, 0.79]	1.00 [0.95, 1.00]
Nikolai, f35.1	132	31	5	96	0	0.86 [0.70, 0.95]	1.00 [0.96, 1.00]
Nikolai, f35.2	132	31	5	96	0	0.86 [0.70, 0.95]	1.00 [0.96, 1.00]

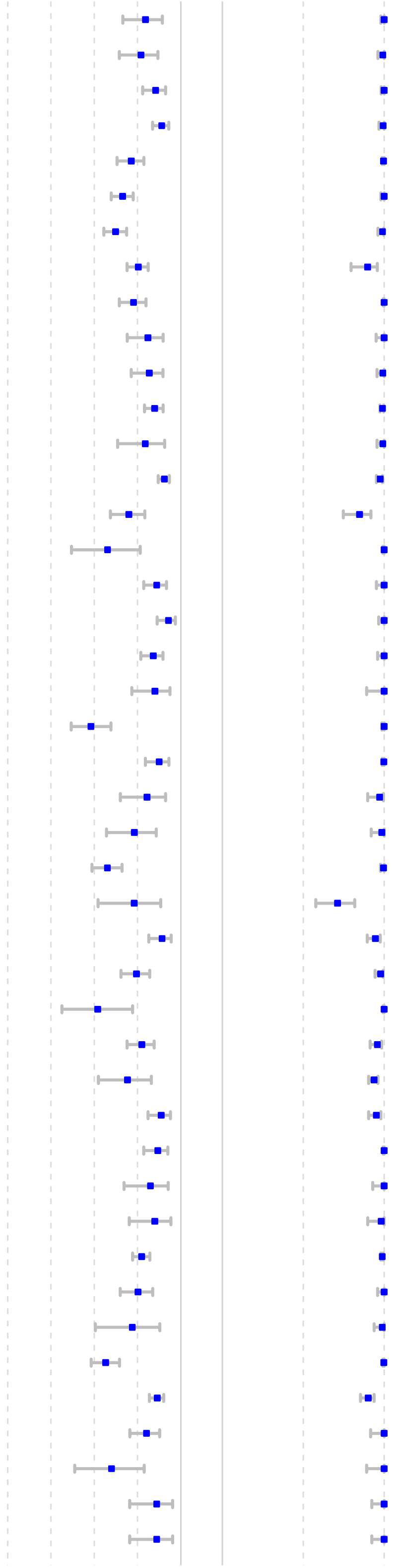

Author, Study ID	Sample size	TP	FN	TN	FP	Sensitivity [95%CI]	Specificity [95%CI]
Nikolai, f35.4	96	31	3	61	1	0.91 [0.76, 0.98]	0.98 [0.91, 1.00]
Nikolai, f35.3	96	31	3	62	0	0.91 [0.76, 0.98]	1.00 [0.94, 1.00]
Pena, f36.1	842	51	22	766	3	0.70 [0.58, 0.80]	1.00 [0.99, 1.00]
FINDdx, f39.1	232	30	11	191	0	0.73 [0.57, 0.86]	1.00 [0.98, 1.00]
FINDdx, f40.1	265	31	13	219	2	0.70 [0.55, 0.83]	0.99 [0.97, 1.00]
FINDdx, f41.1	328	48	8	272	0	0.86 [0.74, 0.94]	1.00 [0.99, 1.00]
FINDdx, f42.1	281	38	6	235	2	0.86 [0.73, 0.95]	0.99 [0.97, 1.00]
FINDdx, f42.2	281	40	4	235	2	0.91 [0.78, 0.98]	0.99 [0.97, 1.00]
Drain, f43.1	257	81	2	168	6	0.98 [0.92, 1.00]	0.97 [0.93, 0.99]
Drain, f43.2	255	39	1	210	5	0.98 [0.87, 1.00]	0.98 [0.95, 0.99]
Okoye, f51.1	2638	24	21	2593	0	0.53 [0.38, 0.68]	1.00 [1.00, 1.00]
Villaverde, f55.1	1620	35	42	1540	3	0.46 [0.34, 0.57]	1.00 [0.99, 1.00]
Krüger, f58.1	761	120	26	611	4	0.82 [0.75, 0.88]	0.99 [0.98, 1.00]
Pollock, f59.1	1498	135	99	1243	21	0.58 [0.51, 0.64]	0.98 [0.98, 0.99]
Shidlovskaya, f61.1	106	44	34	28	0	0.56 [0.45, 0.68]	1.00 [0.88, 1.00]
Shidlovskaya, f61.2	106	41	37	27	1	0.53 [0.41, 0.64]	0.96 [0.82, 1.00]
Faíco−Filho, f63.1	127	59	11	56	1	0.84 [0.74, 0.92]	0.98 [0.91, 1.00]
Schuit, f64.2	1596	83	49	1456	8	0.63 [0.54, 0.71]	1.00 [0.99, 1.00]
Stokes, f65.1	1641	231	37	1371	2	0.86 [0.81, 0.90]	1.00 [1.00, 1.00]
Kilic, f71.1	1384	77	39	1253	15	0.66 [0.57, 0.75]	0.99 [0.98, 0.99]
L...Huillier, f72.1	822	78	41	702	1	0.66 [0.56, 0.74]	1.00 [0.99, 1.00]
Osmanodja, f79.1	379	62	8	308	1	0.89 [0.79, 0.95]	1.00 [0.98, 1.00]
Homza, f87.1	318	81	26	204	7	0.76 [0.66, 0.84]	0.97 [0.93, 0.99]
Homza, f87.2	225	52	38	133	2	0.58 [0.47, 0.68]	0.98 [0.95, 1.00]
Homza, f87.3	191	54	23	64	50	0.70 [0.59, 0.80]	0.56 [0.47, 0.65]
Homza, f87.4	139	26	16	96	1	0.62 [0.46, 0.76]	0.99 [0.94, 1.00]
Homza, f87.5	268	38	53	170	7	0.42 [0.32, 0.53]	0.96 [0.92, 0.98]
Thakur, f88.1	677	29	55	592	1	0.34 [0.24, 0.46]	1.00 [0.99, 1.00]
FINDdx, f91.2	218	71	8	137	2	0.90 [0.81, 0.96]	0.99 [0.95, 1.00]
FINDdx, f91.1	218	71	8	137	2	0.90 [0.81, 0.96]	0.99 [0.95, 1.00]
FINDdx, f92.1	723	88	24	611	0	0.79 [0.70, 0.86]	1.00 [0.99, 1.00]
FINDdx, f93.1	665	176	18	471	0	0.91 [0.86, 0.94]	1.00 [0.99, 1.00]
FINDdx, f94.1	462	61	8	390	3	0.88 [0.78, 0.95]	0.99 [0.98, 1.00]
Homza, f99.1	494	125	39	321	9	0.76 [0.69, 0.82]	0.97 [0.95, 0.99]
Bianco, f102.1	907	269	29	561	48	0.90 [0.86, 0.93]	0.92 [0.90, 0.94]
Chiu, f107.1	349	64	11	260	14	0.85 [0.75, 0.92]	0.95 [0.92, 0.97]
Chiu, f107.2	349	62	13	264	10	0.83 [0.72, 0.90]	0.96 [0.93, 0.98]
Chiu, f107.3	22994	32	6	22938	18	0.84 [0.69, 0.94]	1.00 [1.00, 1.00]
Caramello, f108.2	149	84	13	46	6	0.87 [0.78, 0.93]	0.88 [0.77, 0.96]
Caramello, f108.1	175	84	29	61	1	0.74 [0.65, 0.82]	0.98 [0.91, 1.00]
Christensen, f109.1	278	52	8	217	1	0.87 [0.75, 0.94]	1.00 [0.98, 1.00]
Bornemann, f112.1	1391	52	39	1291	9	0.57 [0.46, 0.68]	0.99 [0.99, 1.00]
Harris, f114.1	885	251	54	573	7	0.82 [0.78, 0.86]	0.99 [0.98, 1.00]
Eleftheriou, f120.1	744	42	9	693	0	0.82 [0.69, 0.92]	1.00 [1.00, 1.00]
Smith, f122.1	2887	180	55	2645	7	0.77 [0.71, 0.82]	1.00 [1.00, 1.00]
Leixner, f125.1	392	65	29	297	1	0.69 [0.59, 0.78]	1.00 [0.98, 1.00]
Fernandez−Montero, f126.1	2543	35	14	2486	8	0.71 [0.57, 0.83]	1.00 [0.99, 1.00]
García−Fiñana, f131.1	5504	28	42	5431	3	0.40 [0.28, 0.52]	1.00 [1.00, 1.00]
Shah, f133.1	2110	272	62	1769	7	0.81 [0.77, 0.86]	1.00 [0.99, 1.00]
Frediani, f139.5	297	57	20	218	2	0.74 [0.63, 0.83]	0.99 [0.97, 1.00]
Di Domenico, f140.2	433	20	16	397	0	0.56 [0.38, 0.72]	1.00 [0.99, 1.00]
Di Domenico, f140.1	433	36	0	382	15	1.00 [0.90, 1.00]	0.96 [0.94, 0.98]

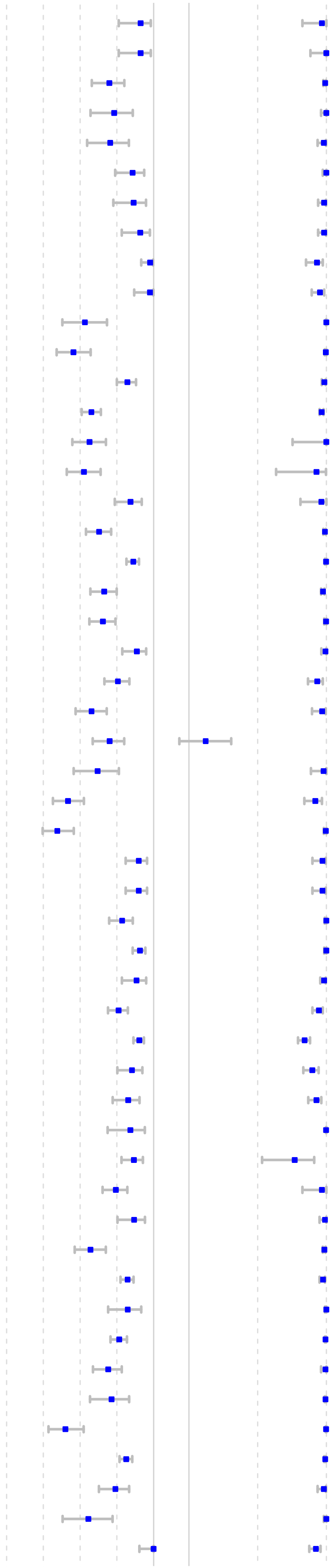

Author, Study ID	Sample size	TP	FN	TN	FP	Sensitivity [95%CI]	Specificity [95%CI]
Di Domenico, f140.2	433	20	16	397	0	0.56 [0.38, 0.72]	1.00 [0.99, 1.00]
Di Domenico, f140.1	433	36	0	382	15	1.00 [0.90, 1.00]	0.96 [0.94, 0.98]
Dankova, f142.1	1227	14	23	1190	0	0.38 [0.22, 0.55]	1.00 [1.00, 1.00]
Menchinelli, f145.1	2898	137	63	2617	81	0.69 [0.62, 0.75]	0.97 [0.96, 0.98]
Wertenauer, f150.2	2215	192	146	1875	2	0.57 [0.51, 0.62]	1.00 [1.00, 1.00]
Wertenauer, f150.1	2215	204	134	1872	5	0.60 [0.55, 0.66]	1.00 [0.99, 1.00]
Agarwal, f153.1	467	26	3	436	2	0.90 [0.73, 0.98]	1.00 [0.98, 1.00]
Bachmann, f154.1	167	75	33	57	2	0.69 [0.60, 0.78]	0.97 [0.88, 1.00]
Bachmann, f154.4	167	75	15	75	2	0.83 [0.74, 0.90]	0.97 [0.91, 1.00]
Bachmann, f154.3	169	89	20	60	0	0.82 [0.73, 0.88]	1.00 [0.94, 1.00]
Bachmann, f154.6	169	84	8	72	5	0.91 [0.84, 0.96]	0.94 [0.86, 0.98]
Bachmann, f154.2	170	81	29	59	1	0.74 [0.64, 0.82]	0.98 [0.91, 1.00]
Bachmann, f154.5	170	79	13	75	3	0.86 [0.77, 0.92]	0.96 [0.89, 0.99]
Tinker, f156.1	1540	8	32	1500	0	0.20 [0.09, 0.36]	1.00 [1.00, 1.00]
Merino−Amador, f160.1	450	179	13	256	2	0.93 [0.89, 0.96]	0.99 [0.97, 1.00]
Kolwijck, f168.1	433	39	6	388	0	0.87 [0.73, 0.95]	1.00 [0.99, 1.00]
Ifko, f169.1	125	20	3	90	12	0.87 [0.66, 0.97]	0.88 [0.80, 0.94]
FINDdx, f175.1	279	58	5	215	1	0.92 [0.82, 0.97]	1.00 [0.97, 1.00]
FINDdx, f176.1	120	33	21	66	0	0.61 [0.47, 0.74]	1.00 [0.95, 1.00]
FINDdx, f177.2	108	39	15	53	1	0.72 [0.58, 0.84]	0.98 [0.90, 1.00]
FINDdx, f177.1	391	50	42	296	3	0.54 [0.44, 0.65]	0.99 [0.97, 1.00]
FINDdx, f178.1	454	94	12	348	0	0.89 [0.81, 0.94]	1.00 [0.99, 1.00]
FINDdx, f179.1	227	72	46	109	0	0.61 [0.52, 0.70]	1.00 [0.97, 1.00]
FINDdx, f180.2	997	102	28	865	2	0.78 [0.70, 0.85]	1.00 [0.99, 1.00]
FINDdx, f181.1	333	59	50	218	6	0.54 [0.44, 0.64]	0.97 [0.94, 0.99]
FINDdx, f181.2	335	71	29	234	1	0.71 [0.61, 0.80]	1.00 [0.98, 1.00]
FINDdx, f182.2	214	66	12	135	1	0.85 [0.75, 0.92]	0.99 [0.96, 1.00]
FINDdx, f182.1	214	66	12	135	1	0.85 [0.75, 0.92]	0.99 [0.96, 1.00]
Kim, f186.2	200	94	6	100	0	0.94 [0.87, 0.98]	1.00 [0.96, 1.00]
Korenkov, f187.1	1849	89	101	1657	2	0.47 [0.40, 0.54]	1.00 [1.00, 1.00]
Mayanskiy, f188.1	277	164	18	72	23	0.90 [0.85, 0.94]	0.76 [0.66, 0.84]
Thirion−Romero, f195.1	1064	258	216	581	9	0.54 [0.50, 0.59]	0.98 [0.97, 0.99]
Cento, f196.1	960	297	50	596	17	0.86 [0.81, 0.89]	0.97 [0.96, 0.98]

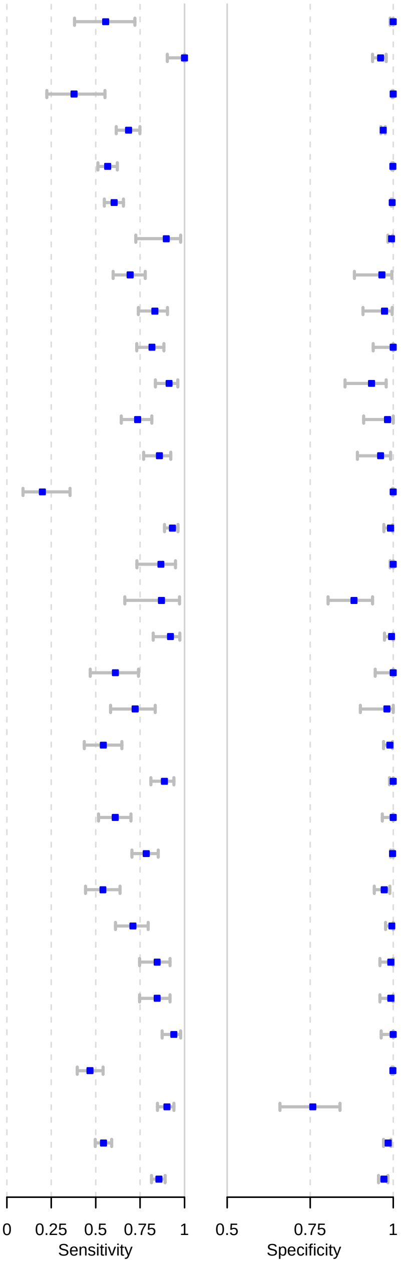

Fig B - Forest plots for non-IFU conforming studies

Author, Study ID	Sample size	TP	FN	TN	FP	Sensitivity [95%CI]	Specificity [95%CI]
Abdulrahman, a01.1	4183	602	131	3420	30	0.82 [0.79, 0.85]	0.99 [0.99, 0.99]
Alemany, a02.2	487	93	24	365	5	0.80 [0.71, 0.86]	0.99 [0.97, 1.00]
Alemany, a02.1	919	779	55	85	0	0.93 [0.92, 0.95]	1.00 [0.96, 1.00]
Cerutti, a08.1	185	75	29	81	0	0.72 [0.62, 0.80]	1.00 [0.96, 1.00]
Krüttgen, a16.1	150	53	22	72	3	0.71 [0.59, 0.81]	0.96 [0.89, 0.99]
Krüger, a17.1	1263	36	11	1207	9	0.77 [0.62, 0.88]	0.99 [0.99, 1.00]
Lambert−Niclot, a18.1	138	47	47	44	0	0.50 [0.40, 0.60]	1.00 [0.92, 1.00]
Lindner, a21.1	289	29	10	248	2	0.74 [0.58, 0.87]	0.99 [0.97, 1.00]
Liotti, a22.1	359	49	55	251	4	0.47 [0.37, 0.57]	0.98 [0.96, 1.00]
Pekosz, a28.1	251	27	1	220	3	0.96 [0.82, 1.00]	0.99 [0.96, 1.00]
Porte, a31.1	127	77	5	45	0	0.94 [0.86, 0.98]	1.00 [0.92, 1.00]
Porte, a32.1	64	30	2	31	1	0.94 [0.79, 0.99]	0.97 [0.84, 1.00]
Porte, a32.2	64	29	3	31	1	0.91 [0.75, 0.98]	0.97 [0.84, 1.00]
Schildgen, a33.2	73	21	21	24	7	0.50 [0.34, 0.66]	0.77 [0.59, 0.90]
Schildgen, a33.1	73	14	28	27	4	0.33 [0.20, 0.50]	0.87 [0.70, 0.96]
Schildgen, a33.3	73	37	5	6	25	0.88 [0.74, 0.96]	0.19 [0.07, 0.38]
Scohy, a34.1	148	32	74	42	0	0.30 [0.22, 0.40]	1.00 [0.92, 1.00]
Weitzel, a41.1	109	49	30	30	0	0.62 [0.50, 0.73]	1.00 [0.88, 1.00]
Weitzel, a41.3	109	13	65	31	0	0.17 [0.09, 0.27]	1.00 [0.89, 1.00]
Weitzel, a41.4	111	68	12	31	0	0.85 [0.75, 0.92]	1.00 [0.89, 1.00]
Young, a43.1	251	29	9	212	1	0.76 [0.60, 0.89]	1.00 [0.97, 1.00]
Mertens, a48.1	328	76	56	195	1	0.58 [0.49, 0.66]	1.00 [0.97, 1.00]
Takeda, a50.1	162	50	12	100	0	0.81 [0.69, 0.90]	1.00 [0.96, 1.00]
Toptan, a55.2	70	16	16	38	0	0.50 [0.32, 0.68]	1.00 [0.91, 1.00]
Toptan, a55.1	67	45	13	9	0	0.78 [0.65, 0.88]	1.00 [0.66, 1.00]
Agullo, a56.2	659	59	73	527	0	0.45 [0.36, 0.54]	1.00 [0.99, 1.00]
Agullo, a56.3	610	28	93	489	0	0.23 [0.16, 0.32]	1.00 [0.99, 1.00]
Chaimao, a57.1	454	59	1	389	5	0.98 [0.91, 1.00]	0.99 [0.97, 1.00]
Halfon, f18.1	200	72	28	99	1	0.72 [0.62, 0.80]	0.99 [0.95, 1.00]
Osterman, f20.1	549	115	74	352	8	0.61 [0.54, 0.68]	0.98 [0.96, 0.99]
Osterman, f20.2	642	165	91	377	9	0.64 [0.58, 0.70]	0.98 [0.96, 0.99]
Kannian, f26.1	37	15	12	10	0	0.56 [0.35, 0.74]	1.00 [0.69, 1.00]
Ngo Nsoga, f28.1	402	136	32	232	2	0.81 [0.74, 0.87]	0.99 [0.97, 1.00]
Favresse, f31.3	188	74	22	89	3	0.77 [0.67, 0.85]	0.97 [0.91, 0.99]
Favresse, f31.1	188	64	32	91	1	0.67 [0.56, 0.76]	0.99 [0.94, 1.00]
Favresse, f31.2	188	65	31	92	0	0.68 [0.57, 0.77]	1.00 [0.96, 1.00]
Favresse, f31.4	188	67	29	92	0	0.70 [0.60, 0.79]	1.00 [0.96, 1.00]
Kohmer, f32.1	100	29	45	25	1	0.39 [0.28, 0.51]	0.96 [0.80, 1.00]
Kohmer, f32.4	100	37	37	26	0	0.50 [0.38, 0.62]	1.00 [0.87, 1.00]
Kohmer, f32.3	100	18	56	26	0	0.24 [0.15, 0.36]	1.00 [0.87, 1.00]
Kohmer, f32.2	100	32	42	26	0	0.43 [0.32, 0.55]	1.00 [0.87, 1.00]
Baro, f33.2	286	52	49	182	3	0.52 [0.41, 0.62]	0.98 [0.95, 1.00]
Baro, f33.4	286	46	55	165	20	0.46 [0.36, 0.56]	0.89 [0.84, 0.93]
Baro, f33.1	286	39	62	184	1	0.39 [0.29, 0.49]	1.00 [0.97, 1.00]
Baro, f33.3	286	44	57	178	7	0.44 [0.34, 0.54]	0.96 [0.92, 0.98]
Baro, f33.5	286	29	72	181	4	0.29 [0.20, 0.39]	0.98 [0.95, 0.99]

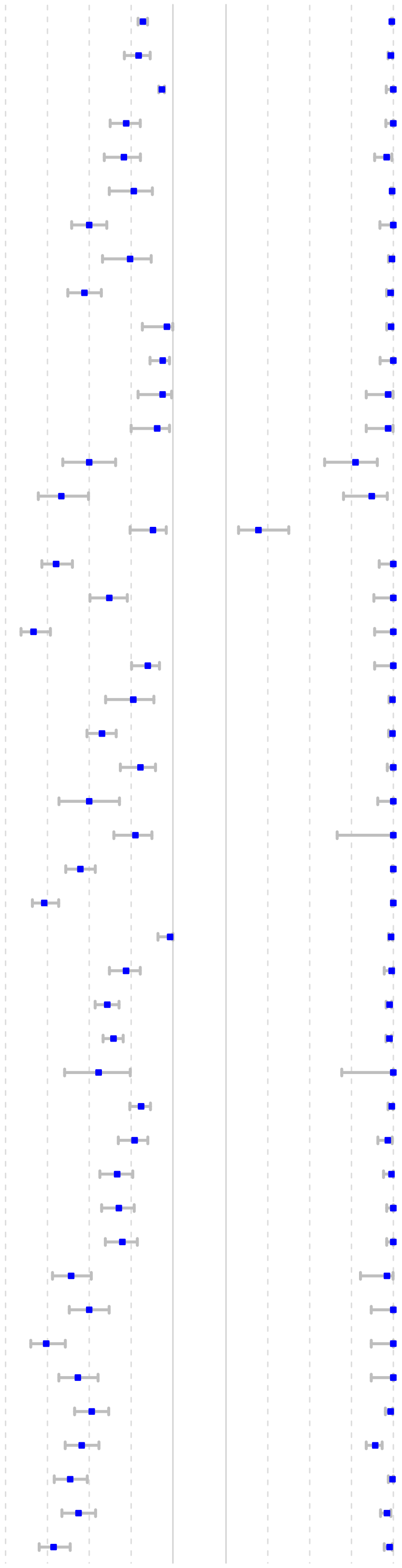

Author, Study ID	Sample size	TP	FN	TN	FP	Sensitivity [95%CI]	Specificity [95%CI]
Caruana, f34.4	532	47	67	417	1	0.41 [0.32, 0.51]	1.00 [0.99, 1.00]
Caruana, f34.3	532	55	59	416	2	0.48 [0.39, 0.58]	1.00 [0.98, 1.00]
Caruana, f34.2	532	47	67	416	2	0.41 [0.32, 0.51]	1.00 [0.98, 1.00]
Caruana, f34.1	532	47	67	417	1	0.41 [0.32, 0.51]	1.00 [0.99, 1.00]
Jääskeläinen, f50.3	190	126	26	38	0	0.83 [0.76, 0.88]	1.00 [0.91, 1.00]
Jääskeläinen, f50.1	188	119	29	40	0	0.80 [0.73, 0.86]	1.00 [0.91, 1.00]
Jääskeläinen, f50.2	198	128	30	40	0	0.81 [0.74, 0.87]	1.00 [0.91, 1.00]
Pérez−García, f52.1	320	91	79	150	0	0.54 [0.46, 0.61]	1.00 [0.98, 1.00]
Pérez−García, f52.2	320	102	68	150	0	0.60 [0.52, 0.67]	1.00 [0.98, 1.00]
Salvagno, f54.1	321	108	41	171	1	0.72 [0.65, 0.80]	0.99 [0.97, 1.00]
Schuit, f64.1	2678	149	84	2436	9	0.64 [0.57, 0.70]	1.00 [0.99, 1.00]
Bouassa, f67.1	150	90	10	50	0	0.90 [0.82, 0.95]	1.00 [0.93, 1.00]
Pickering, f73.6	200	69	31	98	2	0.69 [0.59, 0.78]	0.98 [0.93, 1.00]
Pickering, f73.5	200	74	26	100	0	0.74 [0.64, 0.82]	1.00 [0.96, 1.00]
Pickering, f73.1	200	89	11	99	1	0.89 [0.81, 0.94]	0.99 [0.95, 1.00]
Pickering, f73.4	200	77	23	98	2	0.77 [0.68, 0.85]	0.98 [0.93, 1.00]
Pickering, f73.3	200	65	35	100	0	0.65 [0.55, 0.74]	1.00 [0.96, 1.00]
Koeleman, f103.2	80	25	15	35	5	0.62 [0.46, 0.77]	0.88 [0.73, 0.96]
Koeleman, f103.3	80	22	18	39	1	0.55 [0.38, 0.71]	0.98 [0.87, 1.00]
Koeleman, f103.6	900	220	80	599	1	0.73 [0.68, 0.78]	1.00 [0.99, 1.00]
Koeleman, f103.1	80	29	11	40	0	0.72 [0.56, 0.85]	1.00 [0.91, 1.00]
Pérez...García, f111.1	356	102	68	186	0	0.60 [0.52, 0.67]	1.00 [0.98, 1.00]
Pérez...García, f111.2	356	113	57	181	5	0.66 [0.59, 0.74]	0.97 [0.94, 0.99]
Blairon, f113.2	199	90	60	49	0	0.60 [0.52, 0.68]	1.00 [0.93, 1.00]
Blairon, f113.3	198	91	58	49	0	0.61 [0.53, 0.69]	1.00 [0.93, 1.00]
Blairon, f113.1	199	89	61	42	7	0.59 [0.51, 0.67]	0.86 [0.73, 0.94]
Nordgren, f117.2	332	124	32	131	45	0.80 [0.72, 0.86]	0.74 [0.67, 0.81]
Nordgren, f117.1	286	112	44	130	0	0.72 [0.64, 0.79]	1.00 [0.97, 1.00]
Lee, f136.1	680	109	271	300	0	0.29 [0.24, 0.34]	1.00 [0.99, 1.00]
Seynaeve, f137.2	100	31	19	50	0	0.62 [0.47, 0.75]	1.00 [0.93, 1.00]
Seynaeve, f137.1	100	44	6	50	0	0.88 [0.76, 0.96]	1.00 [0.93, 1.00]
Lunca, f138.1	47	23	17	7	0	0.58 [0.41, 0.73]	1.00 [0.59, 1.00]
Van Honacker, f143.1	97	52	6	18	21	0.90 [0.79, 0.96]	0.46 [0.30, 0.63]
Van Honacker, f143.3	98	48	10	37	3	0.83 [0.71, 0.91]	0.92 [0.80, 0.98]
Van Honacker, f143.4	97	45	12	40	0	0.79 [0.66, 0.89]	1.00 [0.91, 1.00]
Van Honacker, f143.5	98	48	10	40	0	0.83 [0.71, 0.91]	1.00 [0.91, 1.00]
Van Honacker, f143.6	4195	200	169	3814	12	0.54 [0.49, 0.59]	1.00 [1.00, 1.00]
Van Honacker, f143.2	98	39	19	40	0	0.67 [0.54, 0.79]	1.00 [0.91, 1.00]
Karon, f148.4	347	153	44	146	4	0.78 [0.71, 0.83]	0.97 [0.93, 0.99]
Karon, f148.3	347	174	23	150	0	0.88 [0.83, 0.92]	1.00 [0.98, 1.00]
Karon, f148.1	347	131	66	150	0	0.66 [0.59, 0.73]	1.00 [0.98, 1.00]
Karon, f148.2	347	164	33	150	0	0.83 [0.77, 0.88]	1.00 [0.98, 1.00]
Kim, f155.1	165	58	7	96	4	0.89 [0.79, 0.96]	0.96 [0.90, 0.99]
Osterman, f157.1	409	25	81	303	0	0.24 [0.16, 0.33]	1.00 [0.99, 1.00]
Kanaujia, f162.1	484	136	53	293	2	0.72 [0.65, 0.78]	0.99 [0.98, 1.00]
Baccani, f165.3	81	9	15	57	0	0.38 [0.19, 0.59]	1.00 [0.94, 1.00]
Baccani, f165.2	93	10	18	65	0	0.36 [0.19, 0.56]	1.00 [0.94, 1.00]
Kahn, f167.1	3110	57	39	2983	31	0.59 [0.49, 0.69]	0.99 [0.98, 0.99]
Johnson, f170.1	100	47	3	50	0	0.94 [0.84, 0.99]	1.00 [0.93, 1.00]
Jegerlehner, f172.1	1462	92	49	1319	2	0.65 [0.57, 0.73]	1.00 [1.00, 1.00]
Onsongo, f174.1	997	109	43	824	21	0.72 [0.64, 0.79]	0.98 [0.96, 0.98]
FINDdx, f180.1	519	91	9	407	12	0.91 [0.84, 0.96]	0.97 [0.95, 0.98]
Kim, f186.1	130	27	3	98	2	0.90 [0.74, 0.98]	0.98 [0.93, 1.00]
Fourati, f190.6	634	178	119	337	0	0.60 [0.54, 0.66]	1.00 [0.99, 1.00]
Fourati, f190.1	634	105	192	337	0	0.35 [0.30, 0.41]	1.00 [0.99, 1.00]
Fourati, f190.5	634	97	200	332	5	0.33 [0.27, 0.38]	0.98 [0.97, 1.00]
Fourati, f190.3	634	163	134	337	0	0.55 [0.49, 0.61]	1.00 [0.99, 1.00]
Fourati, f190.2	634	180	117	314	23	0.61 [0.55, 0.66]	0.93 [0.90, 0.96]
Fourati, f190.4	634	183	114	337	0	0.62 [0.56, 0.67]	1.00 [0.99, 1.00]
Orsi, f191.2	110	52	8	50	0	0.87 [0.75, 0.94]	1.00 [0.93, 1.00]
Orsi, f191.1	110	56	4	50	0	0.93 [0.84, 0.98]	1.00 [0.93, 1.00]

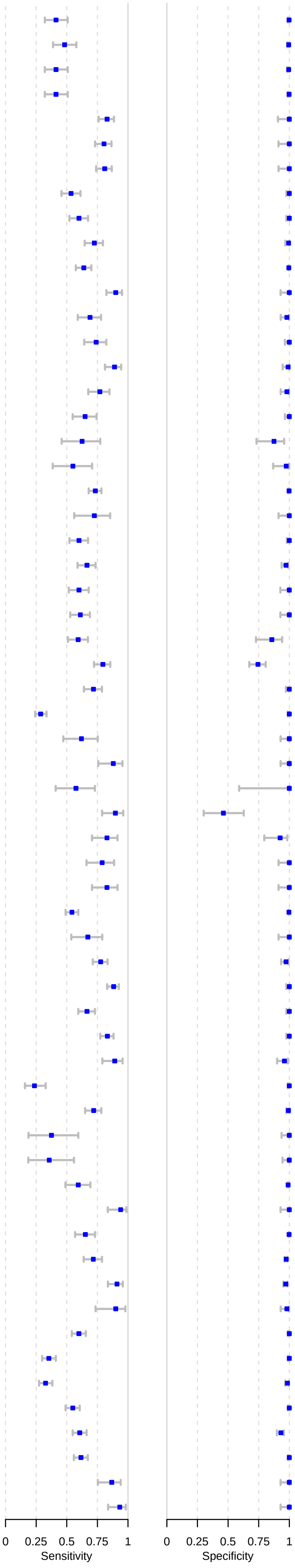

Supplement: S5 Fig — CI, confidence interval; FN, false negative; FP, false positive; IFU, instructions for use; TN, true negative; TP, true positive. (PDF) [file pmed.1004011.s006.pdf]
