# Supplementary material for: Accuracy of rapid point-of-care antigen-based diagnostics for SARS-CoV-2: An updated systematic review and meta-analysis with meta-regression analyzing influencing factors
Source: PLoS Med. 2022 May 26;19(5):e1004011. doi: 10.1371/journal.pmed.1004011 (PMC9187092; doi:10.1371/journal.pmed.1004011)

S7 Fig. Forest plots for subgroup analysis by symptomatic vs. asymptomatic.

Caption: CI = confidence interval

Figure A - Forest plots of asymptomatic patients

Author, Study ID	Sample size	Sensitivity [95%CI]
Alemany, a02.ov	117	0.80 [0.71, 0.86]
Bulilete, a07.1	53	0.57 [0.42, 0.70]
Fenollar. F., a11.1	22	0.46 [0.24, 0.68]
Gupta, a13.1	13	0.69 [0.39, 0.91]
Krüger, a17.1	7	0.57 [0.18, 0.90]
Linares, a20.1	10	0.50 [0.19, 0.81]
Merino−Amador, a25.1	31	1.00 [0.89, 1.00]
Scohy, a34.1	14	0.29 [0.08, 0.58]
Shrestha, a36.1	47	0.85 [0.72, 0.94]
Torres, a37.1	79	0.48 [0.37, 0.60]
Krüger, a52.ov	14	0.86 [0.57, 0.98]
Agullo, a56.2	35	0.23 [0.10, 0.40]
Agullo, a56.3	32	0.09 [0.02, 0.25]
Parada−Ricart, a58.1	1	0.00 [0.00, 0.98]
FINDdx, a62.2	8	0.25 [0.03, 0.65]
FINDdx, a63.2	1	0.00 [0.00, 0.98]
Turcato, f09.1	54	0.50 [0.36, 0.64]
Takeuchi, f12.1	33	0.76 [0.58, 0.89]
Pollock, f17.1	57	0.70 [0.57, 0.82]
Pollock, f17.1	107	0.65 [0.56, 0.74]
Halfon, f18.1	35	0.57 [0.39, 0.74]
James, f23.1	128	0.52 [0.43, 0.60]
Torres, f29.ov	25	0.60 [0.39, 0.79]
Baro, f33.1	101	0.39 [0.29, 0.49]
Baro, f33.2	101	0.52 [0.41, 0.62]
Baro, f33.3	101	0.44 [0.34, 0.54]
Baro, f33.4	101	0.46 [0.36, 0.56]
Baro, f33.5	101	0.29 [0.20, 0.39]
Pena, f36.1	73	0.70 [0.58, 0.80]
FINDdx, f42.1	8	0.62 [0.24, 0.92]
FINDdx, f42.2	8	0.88 [0.47, 1.00]
Okoye, f51.1	45	0.53 [0.38, 0.68]
Krüger, f58.1	9	0.78 [0.40, 0.97]
Pollock, f59.1	128	0.50 [0.41, 0.59]
Pollock, f59.1	37	0.51 [0.34, 0.68]

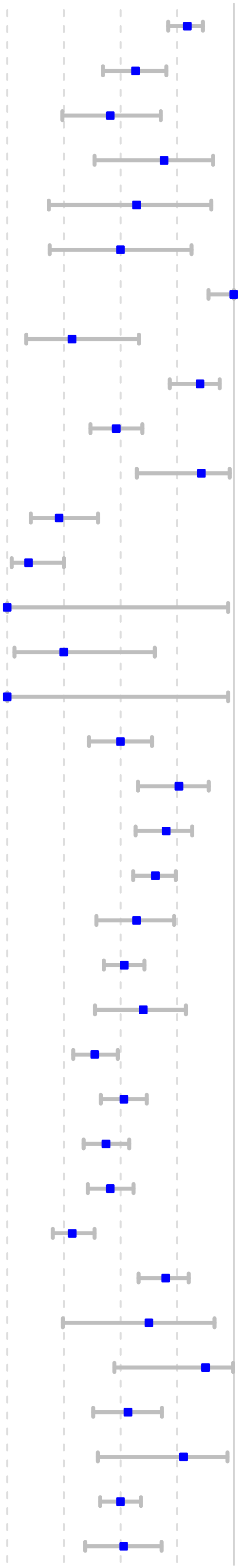

Stokes, f65.1	268	0.86 [0.81, 0.90]
Del Vecchio, f66.1	10	0.90 [0.56, 1.00]
L...Huillier, f72.1	30	0.43 [0.26, 0.63]
Osmanodja, f79.1	1	0.00 [0.00, 0.98]
Thakur, f88.1	84	0.34 [0.24, 0.46]
FINDdx, f92.1	15	0.47 [0.21, 0.73]
FINDdx, f93.1	15	0.73 [0.45, 0.92]
Brihn, f98.1	81	0.60 [0.49, 0.71]
Homza, f99.1	28	0.46 [0.28, 0.66]
Bianco, f102.1	101	0.92 [0.85, 0.96]
Chiu, f107.3	38	0.84 [0.69, 0.94]
Smith, f122.1	42	0.79 [0.63, 0.90]
Abdul−Mumin, f123.1	18	0.72 [0.47, 0.90]
Fernandez−Montero, f126.1	49	0.71 [0.57, 0.83]
Fernández, f128.1	9	0.78 [0.40, 0.97]
García−Fiñana, f131.1	70	0.40 [0.28, 0.52]
Shah, f133.1	48	0.69 [0.54, 0.81]
Di Domenico, f140.1	36	1.00 [0.90, 1.00]
Di Domenico, f140.2	36	0.56 [0.38, 0.72]
Menchinelli, f145.1	66	0.27 [0.17, 0.40]
Leli, f151.1	54	0.48 [0.34, 0.62]
Kurihara, f152.1	47	0.64 [0.48, 0.77]
Kim, f155.1	4	1.00 [0.40, 1.00]
Tinker, f156.1	40	0.20 [0.09, 0.36]
Merino−Amador, f160.1	25	0.92 [0.74, 0.99]
Allan−Blitz, f161.1	958	0.44 [0.41, 0.47]
Kanaujia, f162.1	41	0.56 [0.40, 0.72]
Kahn, f167.1	42	0.48 [0.32, 0.64]
Ifko, f169.1	16	0.56 [0.30, 0.80]
Jegerlehner, f172.1	25	0.44 [0.24, 0.65]
FINDdx, f180.1	10	1.00 [0.69, 1.00]
Kim, f186.1	4	0.75 [0.19, 0.99]
Thirion−Romero, f195.1	57	0.26 [0.16, 0.40]

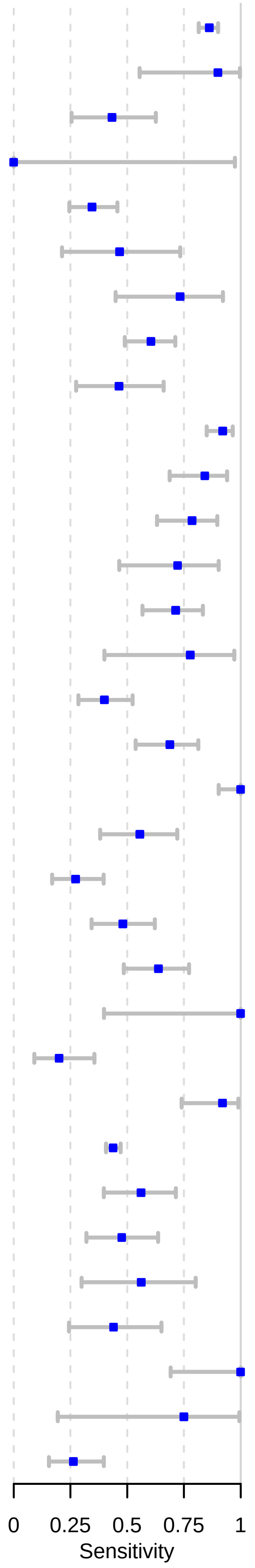

Figure B - Forest plots of symptomatic patients

Author, Study ID	Sample size	Sensitivity [95%CI]
Abdulrahman, a01.1	733	0.82 [0.79, 0.85]
Alemany, a02.ov	419	0.93 [0.90, 0.95]
Albert, a03.1	54	0.80 [0.66, 0.89]
Beck, a04.1	61	0.77 [0.64, 0.87]
Bulilete, a07.1	87	0.80 [0.71, 0.88]
Cerutti, a08.1	104	0.72 [0.62, 0.80]
Fenollar. F., a11.1	182	0.79 [0.72, 0.85]
Gupta, a13.1	64	0.84 [0.73, 0.92]
Krüger, a17.1	40	0.80 [0.64, 0.91]
Linares, a20.1	50	0.78 [0.64, 0.88]
Lindner, a21.1	39	0.74 [0.58, 0.87]
Lindner, a21.2	39	0.80 [0.64, 0.91]
Merino−Amador, a25.1	328	0.90 [0.86, 0.93]
Pekosz, a28.1	28	0.96 [0.82, 1.00]
Porte, a31.1	82	0.94 [0.86, 0.98]
Scohy, a34.1	77	0.32 [0.22, 0.44]
Schwob, a35.1	112	0.93 [0.86, 0.97]
Schwob, a35.2	122	0.86 [0.79, 0.92]
Schwob, a35.3	138	0.84 [0.77, 0.90]
Weitzel, a41.1	79	0.62 [0.50, 0.73]
Weitzel, a41.3	78	0.17 [0.09, 0.27]
Weitzel, a41.4	80	0.85 [0.75, 0.92]
Young, a43.1	38	0.76 [0.60, 0.89]
Herrera, a46.1	459	0.77 [0.72, 0.80]
Mertens, a48.1	132	0.58 [0.49, 0.66]
Krüger, a52.ov	92	0.87 [0.78, 0.93]
Lindner, a53.1	39	0.72 [0.55, 0.85]
Lindner, a53.2	39	0.80 [0.64, 0.91]
Agullo, a56.2	97	0.53 [0.42, 0.63]
Agullo, a56.3	89	0.28 [0.19, 0.39]
Parada−Ricart, a58.1	25	0.76 [0.55, 0.91]
Chaimao, a57.1	56	0.98 [0.90, 1.00]
FINDdx, a61.1	102	0.89 [0.81, 0.94]
FINDdx, a62.2	17	0.65 [0.38, 0.86]
FINDdx, a63.2	38	0.71 [0.54, 0.85]
Turcato, f09.1	169	0.90 [0.84, 0.94]
Takeuchi, f12.1	72	0.92 [0.83, 0.97]
Filgueiras, f14.1	54	0.69 [0.54, 0.80]

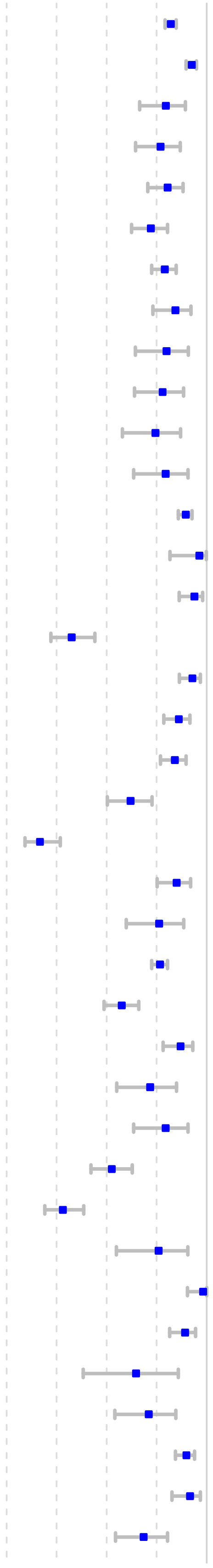

Lindner, f15.1	40	0.85 [0.70, 0.94]
Lindner, f15.2	40	0.82 [0.67, 0.93]
Pollock, f17.1	128	0.91 [0.84, 0.95]
Pollock, f17.1	100	0.92 [0.85, 0.96]
Pollock, f17.1	28	0.86 [0.67, 0.96]
Halfon, f18.1	51	0.78 [0.65, 0.89]
Möckel, f19.1	89	0.75 [0.65, 0.84]
Möckel, f19.2	25	0.72 [0.51, 0.88]
James, f23.1	24	0.83 [0.63, 0.95]
Ngo Nsoga, f28.1	168	0.81 [0.74, 0.87]
Torres, f29.ov	91	0.80 [0.71, 0.88]
Akingba, f30.1	145	0.70 [0.62, 0.77]
Nikolai, f35.1	36	0.86 [0.70, 0.95]
Nikolai, f35.2	36	0.86 [0.70, 0.95]
Nikolai, f35.3	33	0.91 [0.76, 0.98]
Nikolai, f35.4	33	0.91 [0.76, 0.98]
FINDdx, f42.1	37	0.89 [0.75, 0.97]
FINDdx, f42.2	37	0.89 [0.75, 0.97]
Ristic, f44.1	43	0.58 [0.42, 0.73]
Marti, f46.2	32	0.94 [0.79, 0.99]
Villaverde, f55.1	77	0.46 [0.34, 0.57]
Krüger, f58.1	137	0.82 [0.75, 0.88]
Pollock, f59.1	60	0.75 [0.62, 0.85]
Pollock, f59.1	9	0.78 [0.40, 0.97]
Takeuchi, f60.1	51	0.72 [0.58, 0.84]
Shidlovskaya, f61.1	78	0.56 [0.45, 0.68]
Shidlovskaya, f61.2	78	0.53 [0.41, 0.64]
Del Vecchio, f66.1	51	0.65 [0.50, 0.78]
Bouassa, f67.1	100	0.90 [0.82, 0.95]
Kilic, f71.1	116	0.66 [0.57, 0.75]
L...Huillier, f72.1	89	0.73 [0.63, 0.82]
Osmanodja, f79.1	69	0.90 [0.80, 0.96]
Thell, f81.1	213	0.80 [0.74, 0.85]
FINDdx, f91.1	79	0.90 [0.81, 0.96]
FINDdx, f91.2	79	0.90 [0.81, 0.96]
FINDdx, f92.1	95	0.84 [0.75, 0.91]
FINDdx, f93.1	176	0.92 [0.86, 0.95]
Brihn, f98.1	68	0.72 [0.60, 0.82]
Homza, f99.1	135	0.82 [0.75, 0.88]
Bianco, f102.1	197	0.89 [0.84, 0.93]
Koeleman, f103.1	40	0.72 [0.56, 0.85]
Koeleman, f103.2	40	0.62 [0.46, 0.77]
Koeleman, f103.3	40	0.55 [0.38, 0.71]
Koeleman, f103.4	40	0.70 [0.54, 0.83]
Koeleman, f103.5	40	0.78 [0.62, 0.89]
Koeleman, f103.6	300	0.73 [0.68, 0.78]

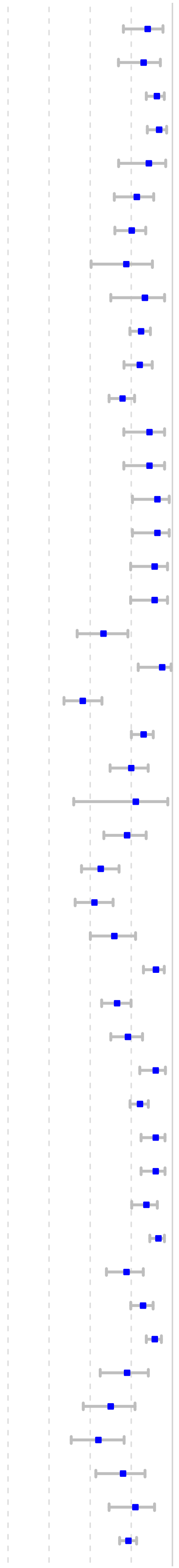

Shaikh, f105.1	39	0.85 [0.70, 0.94]
Chiu, f107.1	75	0.85 [0.75, 0.92]
Chiu, f107.2	75	0.83 [0.72, 0.90]
Christensen, f109.1	60	0.87 [0.75, 0.94]
Pérez...García, f111.1	128	0.63 [0.54, 0.72]
Pérez...García, f111.2	128	0.68 [0.59, 0.76]
Harris, f114.1	305	0.82 [0.78, 0.86]
Nordgren, f117.1	156	0.72 [0.64, 0.79]
Nordgren, f117.2	156	0.80 [0.72, 0.86]
Smith, f122.1	193	0.76 [0.70, 0.82]
Abdul−Mumin, f123.1	24	0.58 [0.37, 0.78]
Leixner, f125.1	94	0.69 [0.59, 0.78]
Fernández, f128.1	15	0.93 [0.68, 1.00]
Terpos, f130.1	114	0.90 [0.82, 0.94]
Shah, f133.1	281	0.79 [0.73, 0.83]
Lunca, f138.1	40	0.58 [0.41, 0.73]
Frediani, f139.5	77	0.74 [0.63, 0.83]
Carbonell−Sahuquillo, f141.1	34	0.71 [0.52, 0.85]
Menchinelli, f145.1	134	0.89 [0.82, 0.94]
Cassuto, f149.1	32	0.97 [0.84, 1.00]
Leli, f151.1	112	0.79 [0.70, 0.86]
Kurihara, f152.1	36	0.89 [0.74, 0.97]
Bachmann, f154.1	108	0.69 [0.60, 0.78]
Bachmann, f154.2	110	0.74 [0.64, 0.82]
Bachmann, f154.3	109	0.82 [0.73, 0.88]
Bachmann, f154.4	90	0.83 [0.74, 0.90]
Bachmann, f154.5	92	0.86 [0.77, 0.92]
Bachmann, f154.6	92	0.91 [0.84, 0.96]
Kim, f155.1	61	0.88 [0.78, 0.95]
Allan−Blitz, f161.1	2107	0.52 [0.50, 0.54]
Kanaujia, f162.1	148	0.76 [0.69, 0.83]
Kahn, f167.1	51	0.67 [0.52, 0.79]
Kolwijck, f168.1	45	0.87 [0.73, 0.95]
Ifko, f169.1	23	0.87 [0.66, 0.97]
Johnson, f170.1	50	0.94 [0.84, 0.99]
Jegerlehner, f172.1	116	0.70 [0.61, 0.78]
FINDdx, f176.1	54	0.61 [0.47, 0.74]
FINDdx, f177.1	92	0.54 [0.44, 0.65]
FINDdx, f177.2	54	0.72 [0.58, 0.84]
FINDdx, f179.1	118	0.61 [0.52, 0.70]
FINDdx, f180.1	89	0.91 [0.83, 0.96]
FINDdx, f181.2	100	0.71 [0.61, 0.80]
FINDdx, f182.1	78	0.85 [0.75, 0.92]
FINDdx, f182.2	78	0.85 [0.75, 0.92]
Kim, f186.1	26	0.92 [0.75, 0.99]
Kim, f186.2	100	0.94 [0.87, 0.98]
Fourati, f190.1	297	0.35 [0.30, 0.41]
Fourati, f190.2	297	0.61 [0.55, 0.66]
Fourati, f190.3	297	0.55 [0.49, 0.61]
Fourati, f190.4	297	0.62 [0.56, 0.67]
Fourati, f190.5	297	0.33 [0.27, 0.38]
Fourati, f190.6	297	0.60 [0.54, 0.66]
Orsi, f191.1	60	0.93 [0.84, 0.98]
Orsi, f191.2	60	0.87 [0.75, 0.94]
Suzuki, f194.1	47	0.85 [0.72, 0.94]
Suzuki, f194.2	47	0.89 [0.77, 0.96]
Thirion−Romero, f195.1	415	0.58 [0.53, 0.63]
Koskinen, f197.1	13	1.00 [0.75, 1.00]

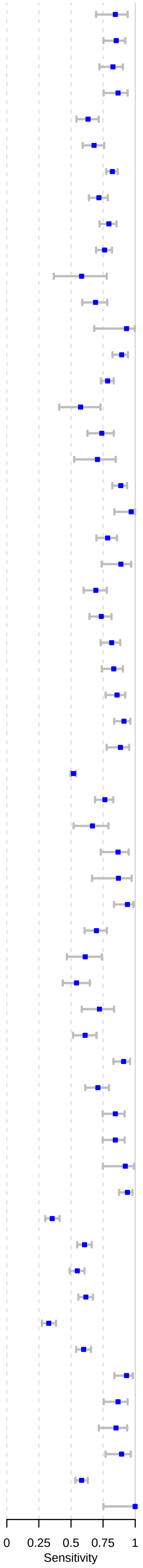

Figure C - Forest plots of patients with symptom onset greater than seven days

Author, Study ID	Sample size	Sensitivity [95%CI]
Gupta, a13.1	7	0.71 [0.29, 0.96]
Krüger, a17.1	4	1.00 [0.40, 1.00]
Linares, a20.1	13	0.54 [0.25, 0.81]
Lindner, a21.1	6	0.17 [0.00, 0.64]
Lindner, a21.2	6	0.33 [0.04, 0.78]
Porte, a31.1	5	0.80 [0.28, 1.00]
Krüger, a52.ov	13	0.62 [0.32, 0.86]
Lindner, a53.1	7	0.29 [0.04, 0.71]
Lindner, a53.2	7	0.71 [0.29, 0.96]
Chaimao, a57.1	12	1.00 [0.74, 1.00]
FINDdx, a62.2	2	0.50 [0.01, 0.99]
FINDdx, a63.2	6	0.17 [0.00, 0.64]
Filgueiras, f14.1	11	0.36 [0.11, 0.69]
Lindner, f15.1	5	0.40 [0.05, 0.85]
Lindner, f15.2	5	0.40 [0.05, 0.85]
Pollock, f17.1	15	0.67 [0.38, 0.88]
Pollock, f17.1	2	1.00 [0.16, 1.00]
Nikolai, f35.3	3	0.67 [0.09, 0.99]
Nikolai, f35.4	3	0.67 [0.09, 0.99]
FINDdx, f42.1	2	0.50 [0.01, 0.99]
FINDdx, f42.2	2	0.50 [0.01, 0.99]
Drain, f43.1	10	0.90 [0.56, 1.00]
Drain, f43.2	6	0.83 [0.36, 1.00]
Pérez−García, f52.1	55	0.49 [0.35, 0.63]
Pérez−García, f52.2	55	0.58 [0.44, 0.71]
Krüger, f58.1	15	0.47 [0.21, 0.73]
Pollock, f59.1	9	0.22 [0.03, 0.60]
Pollock, f59.1	2	0.50 [0.01, 0.99]
Shidlovskaya, f61.1	34	0.47 [0.30, 0.65]
Shidlovskaya, f61.2	34	0.41 [0.25, 0.59]
Del Vecchio, f66.1	12	0.33 [0.10, 0.65]
Thell, f81.1	24	0.62 [0.41, 0.81]
FINDdx, f92.1	6	0.33 [0.04, 0.78]
FINDdx, f93.1	12	0.75 [0.43, 0.94]
Pérez...García, f111.1	55	0.31 [0.19, 0.45]
Pérez...García, f111.2	55	0.38 [0.25, 0.52]
Kim, f155.1	20	0.75 [0.51, 0.91]
FINDdx, f180.1	11	0.54 [0.23, 0.83]
Fourati, f190.1	89	0.16 [0.09, 0.25]
Fourati, f190.2	89	0.37 [0.27, 0.48]
Fourati, f190.3	89	0.35 [0.25, 0.46]
Fourati, f190.4	89	0.42 [0.31, 0.52]
Fourati, f190.5	89	0.15 [0.08, 0.24]
Fourati, f190.6	89	0.43 [0.32, 0.54]
Orsi, f191.1	15	0.87 [0.60, 0.98]
Orsi, f191.2	15	0.80 [0.52, 0.96]
Thirion−Romero, f195.1	139	0.37 [0.29, 0.45]

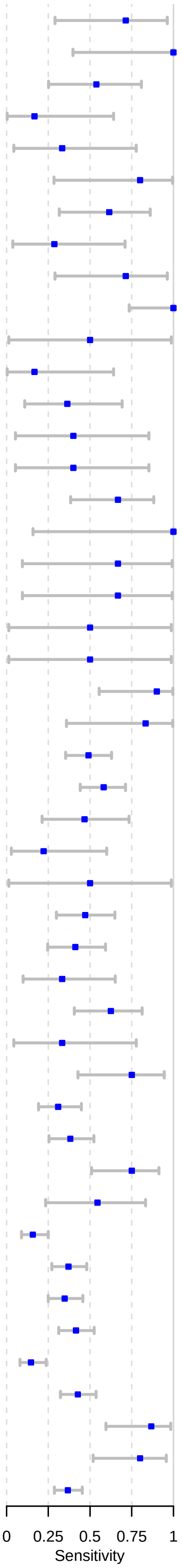

Figure D - Forest plots of patients with symptom onset less than seven days

Author, Study ID	Sample size	Sensitivity [95%CI]
Gupta, a13.1	57	0.86 [0.74, 0.94]
Krüger, a17.1	34	0.79 [0.62, 0.91]
Linares, a20.1	37	0.86 [0.71, 0.96]
Lindner, a21.1	33	0.85 [0.68, 0.95]
Lindner, a21.2	33	0.88 [0.72, 0.97]
Pekosz, a28.1	28	0.96 [0.82, 1.00]
Porte, a31.1	76	0.95 [0.87, 0.98]
Young, a43.1	38	0.76 [0.60, 0.89]
Krüger, a52.ov	76	0.91 [0.82, 0.96]
Lindner, a53.1	32	0.81 [0.64, 0.93]
Lindner, a53.2	32	0.81 [0.64, 0.93]
Chaimao, a57.1	44	0.98 [0.88, 1.00]
FINDdx, a62.2	13	0.77 [0.46, 0.95]
FINDdx, a63.2	32	0.81 [0.64, 0.93]
Filgueiras, f14.1	43	0.77 [0.61, 0.88]
Lindner, f15.1	35	0.89 [0.73, 0.97]
Lindner, f15.2	35	0.91 [0.77, 0.98]
Pollock, f17.1	85	0.96 [0.90, 0.99]
Pollock, f17.1	26	0.85 [0.65, 0.96]
Nikolai, f35.1	36	0.86 [0.70, 0.95]
Nikolai, f35.2	36	0.86 [0.70, 0.95]
Nikolai, f35.3	30	0.93 [0.78, 0.99]
Nikolai, f35.4	30	0.93 [0.78, 0.99]
FINDdx, f42.1	34	0.94 [0.80, 0.99]
FINDdx, f42.2	34	0.91 [0.76, 0.98]
Drain, f43.1	73	0.99 [0.93, 1.00]
Drain, f43.2	34	1.00 [0.90, 1.00]
Pérez−García, f52.1	46	0.85 [0.71, 0.94]
Pérez−García, f52.2	46	0.91 [0.79, 0.98]
Villaverde, f55.1	77	0.46 [0.34, 0.57]
Krüger, f58.1	118	0.86 [0.79, 0.92]
Pollock, f59.1	51	0.84 [0.71, 0.93]
Pollock, f59.1	7	0.86 [0.42, 1.00]
Shidlovskaya, f61.1	44	0.64 [0.48, 0.78]
Shidlovskaya, f61.2	44	0.61 [0.46, 0.76]
Del Vecchio, f66.1	39	0.74 [0.58, 0.87]
Osmanodja, f79.1	69	0.90 [0.80, 0.96]
Thell, f81.1	189	0.82 [0.76, 0.88]

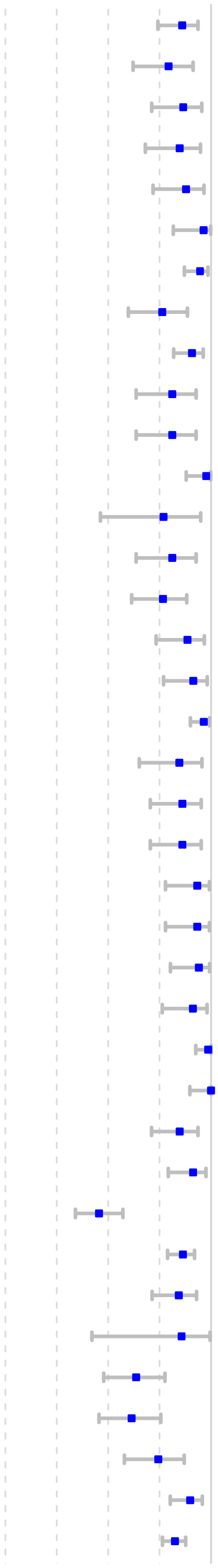

FINDdx, f92.1	87	0.88 [0.80, 0.94]
FINDdx, f93.1	162	0.93 [0.88, 0.97]
Pérez...García, f111.1	73	0.88 [0.78, 0.94]
Pérez...García, f111.2	73	0.90 [0.81, 0.96]
Terpos, f130.1	114	0.90 [0.82, 0.94]
Shah, f133.1	243	0.82 [0.76, 0.86]
Frediani, f139.5	77	0.74 [0.63, 0.83]
Carbonell−Sahuquillo, f141.1	34	0.71 [0.52, 0.85]
Bachmann, f154.1	108	0.69 [0.60, 0.78]
Bachmann, f154.2	110	0.74 [0.64, 0.82]
Bachmann, f154.3	109	0.82 [0.73, 0.88]
Bachmann, f154.4	90	0.83 [0.74, 0.90]
Bachmann, f154.5	92	0.86 [0.77, 0.92]
Bachmann, f154.6	92	0.91 [0.84, 0.96]
Kim, f155.1	41	0.95 [0.84, 0.99]
FINDdx, f175.1	51	0.92 [0.81, 0.98]
FINDdx, f176.1	50	0.62 [0.47, 0.75]
FINDdx, f177.1	85	0.54 [0.43, 0.65]
FINDdx, f177.2	45	0.78 [0.63, 0.89]
FINDdx, f178.1	101	0.90 [0.82, 0.95]
FINDdx, f179.1	111	0.61 [0.52, 0.70]
FINDdx, f180.1	78	0.96 [0.89, 0.99]
FINDdx, f180.2	29	0.76 [0.56, 0.90]
FINDdx, f181.1	96	0.58 [0.48, 0.68]
FINDdx, f181.2	70	0.81 [0.70, 0.90]
FINDdx, f182.1	66	0.82 [0.70, 0.90]
FINDdx, f182.2	66	0.82 [0.70, 0.90]
Fourati, f190.1	200	0.45 [0.38, 0.52]
Fourati, f190.2	200	0.71 [0.64, 0.77]
Fourati, f190.3	200	0.66 [0.58, 0.72]
Fourati, f190.4	200	0.71 [0.64, 0.77]
Fourati, f190.5	200	0.41 [0.34, 0.48]
Fourati, f190.6	200	0.69 [0.62, 0.75]
Orsi, f191.1	45	0.96 [0.85, 1.00]
Orsi, f191.2	45	0.89 [0.76, 0.96]
Thirion−Romero, f195.1	276	0.70 [0.64, 0.75]

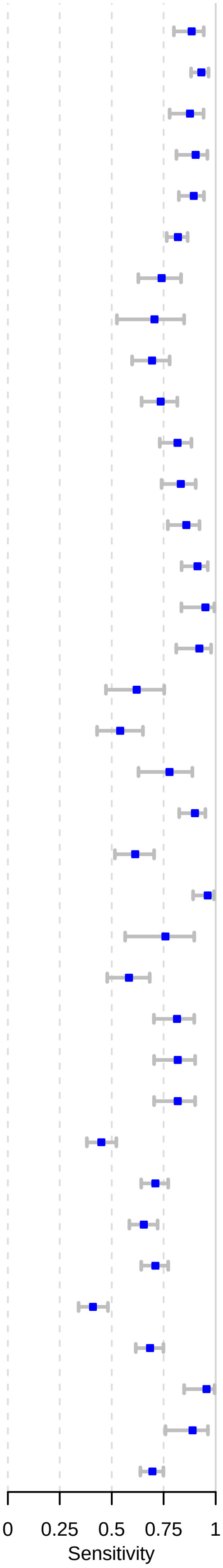

Supplement: S7 Fig — CI, confidence interval. (PDF) [file pmed.1004011.s008.pdf]
