# Supplementary material for: Accuracy of rapid point-of-care antigen-based diagnostics for SARS-CoV-2: An updated systematic review and meta-analysis with meta-regression analyzing influencing factors
Source: PLoS Med. 2022 May 26;19(5):e1004011. doi: 10.1371/journal.pmed.1004011 (PMC9187092; doi:10.1371/journal.pmed.1004011)

S8 Fig. Forest plot for subgroup analysis by mean Ct-values for TP and FN samples

Caption: CI = confidence interval

Subgroup	N Datasets	Total Sample Size	Pooled Mean Ct−value (95% CI)
True Positives	69	5083	22.2 (21.5, 22.8)
False Negatives	76	2390	30.2 (29.6, 30.9)

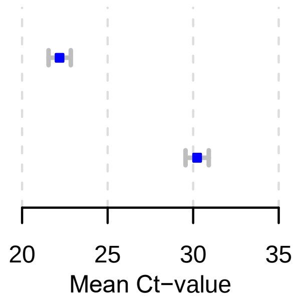

Supplement: S8 Fig — CI, confidence interval; Ct, cycle threshold; FN, false negative; TP, true positive. (PDF) [file pmed.1004011.s009.pdf]
