# Supplementary material for: Accuracy of rapid point-of-care antigen-based diagnostics for SARS-CoV-2: An updated systematic review and meta-analysis with meta-regression analyzing influencing factors
Source: PLoS Med. 2022 May 26;19(5):e1004011. doi: 10.1371/journal.pmed.1004011 (PMC9187092; doi:10.1371/journal.pmed.1004011)

S9 Fig. Forest plots for subgroup analysis by mean Ct-values for TP and FN samples

Caption: CI = confidence interval

Figure A – Forest plots of mean Ct-value for true positive samples

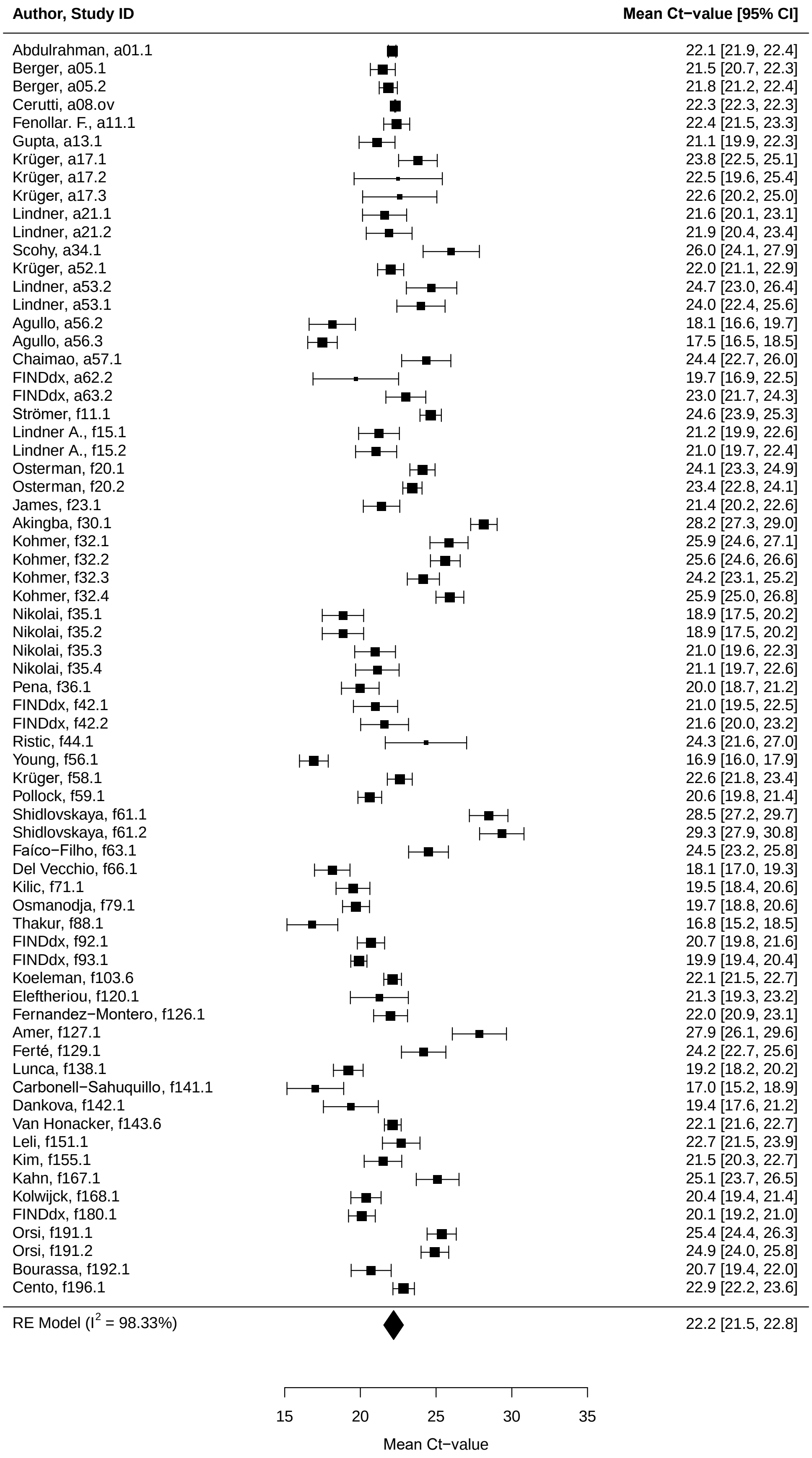

Figure B – Forest plots of mean Ct-value for false negative samples

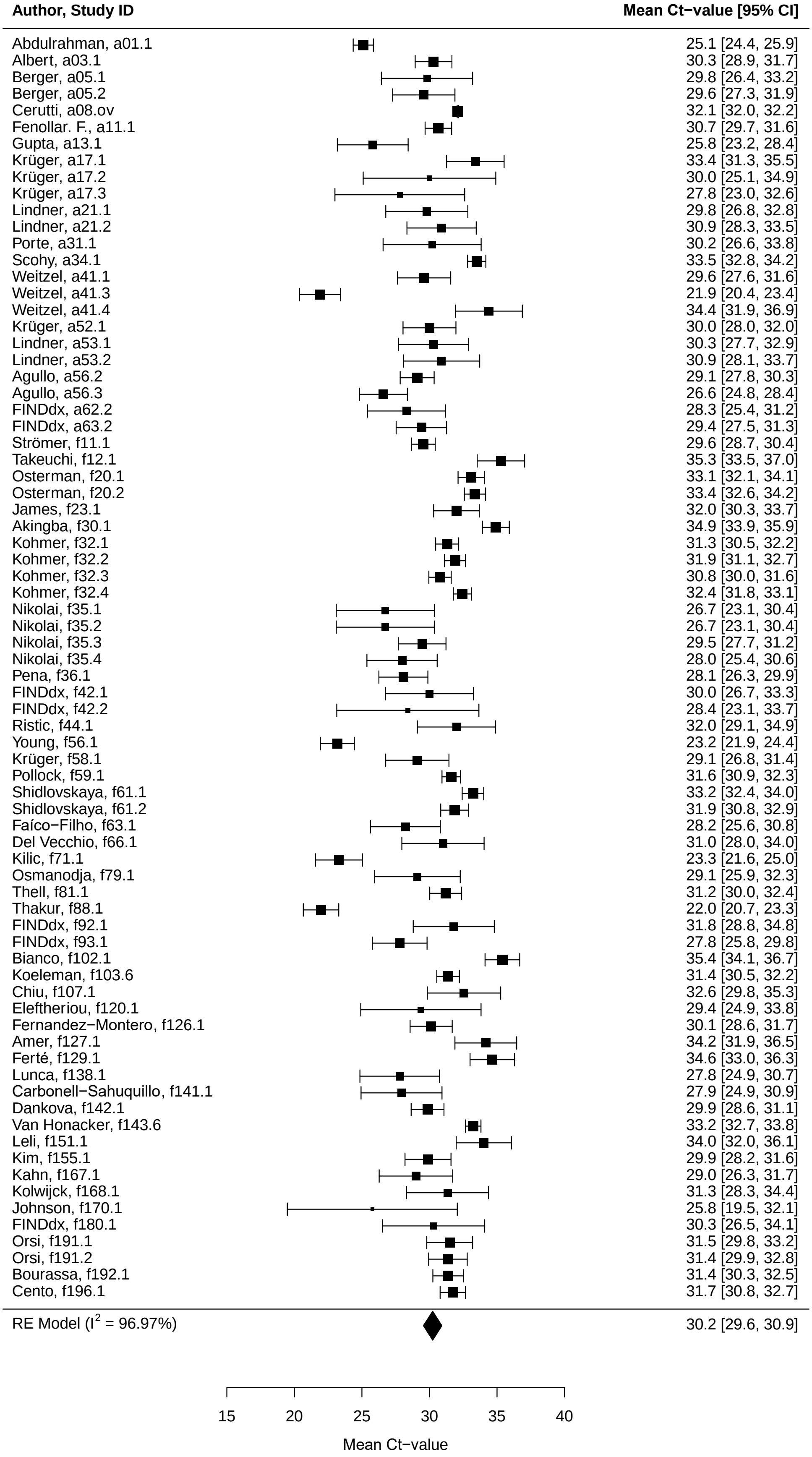

Supplement: S9 Fig — CI, confidence interval; Ct, cycle threshold; FN, false negative; TP, true positive. (PDF) [file pmed.1004011.s010.pdf]
