# Supplementary material for: Accuracy of rapid point-of-care antigen-based diagnostics for SARS-CoV-2: An updated systematic review and meta-analysis with meta-regression analyzing influencing factors
Source: PLoS Med. 2022 May 26;19(5):e1004011. doi: 10.1371/journal.pmed.1004011 (PMC9187092; doi:10.1371/journal.pmed.1004011)

S10 Fig. HSROC curve Standard Q nasal and LumiraDx Ag-RDT

Caption: HSROC = Hierarchical summary receiver-operating characteristic

Fig A – HSROC curve Standard Q nasal

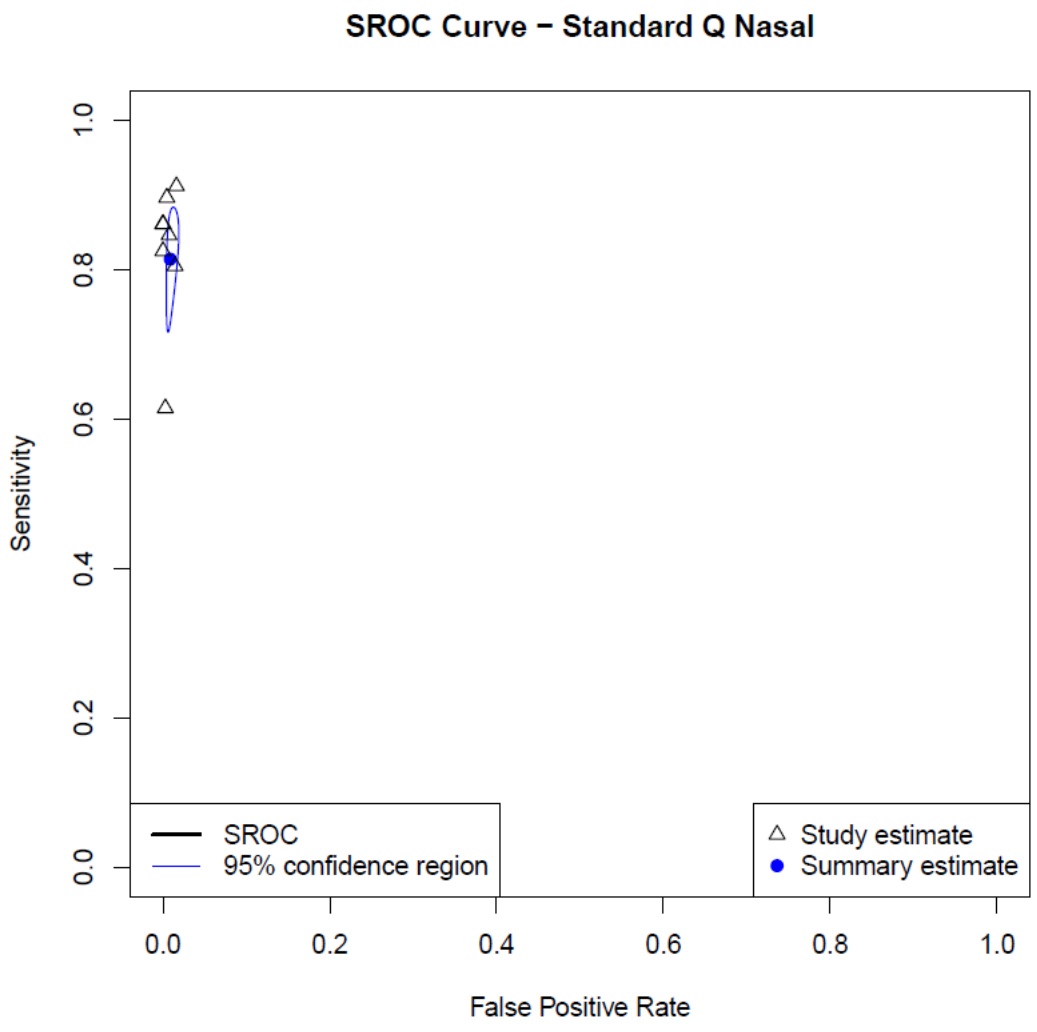

Fig B – HSROC curve LumiraDx

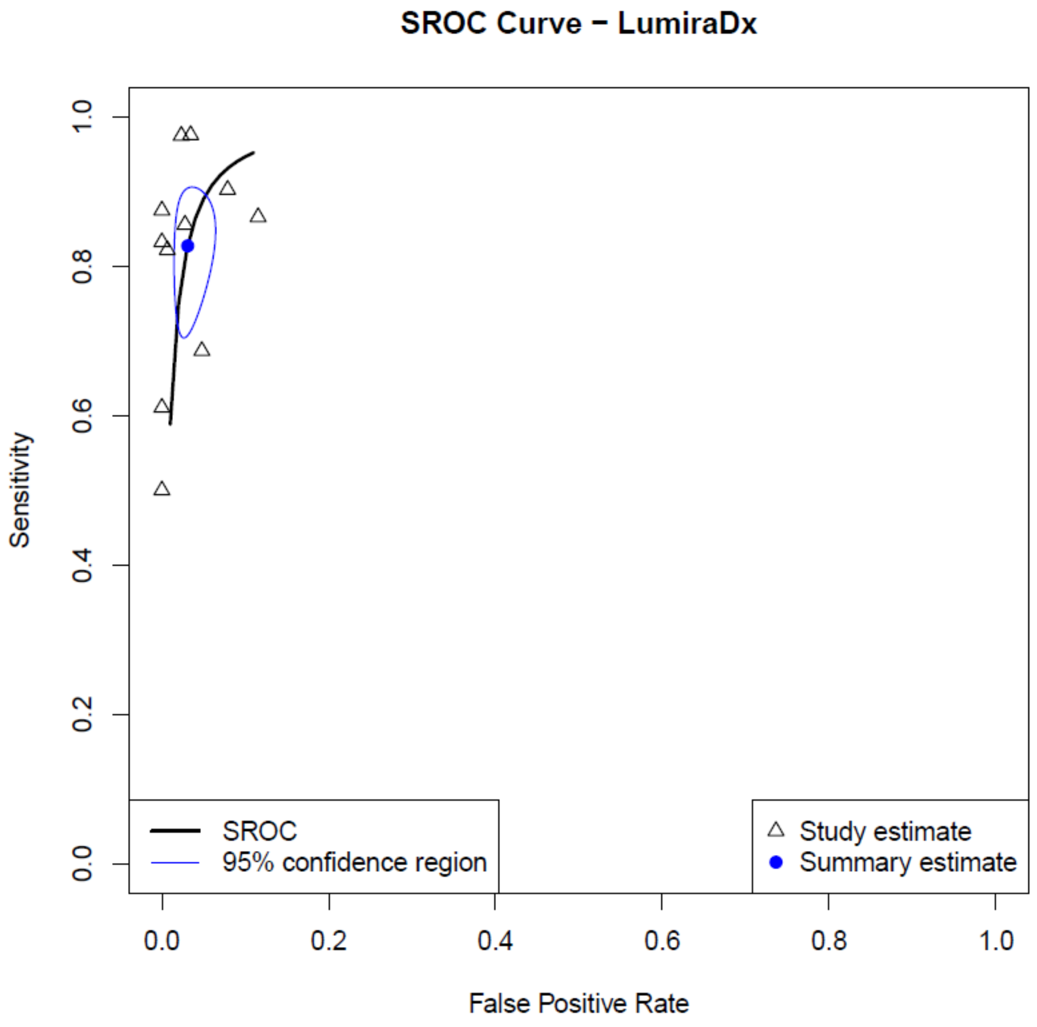

Supplement: S10 Fig — Ag-RDT, antigen rapid diagnostic test; HSROC, Hierarchical summary receiver-operating characteristic. (PDF) [file pmed.1004011.s011.pdf]
