# Supplementary material for: Accuracy of rapid point-of-care antigen-based diagnostics for SARS-CoV-2: An updated systematic review and meta-analysis with meta-regression analyzing influencing factors
Source: PLoS Med. 2022 May 26;19(5):e1004011. doi: 10.1371/journal.pmed.1004011 (PMC9187092; doi:10.1371/journal.pmed.1004011)

S11 Fig. Forest plot for univariate analysis for Nadal and SureScreen V

Caption: CI = confidence interval

Test assessed	Sensitivity N datasets	Sensitivity Total Sample Size	Pooled Sensitivity (95% CI)	Specificity N datasets	Specificity Total Sample Size	Pooled Specificity (95% CI)
Nadal	5	1026	56.8% (29.6−84)	4	902	91.1% (80.2−100)
SureScreen V	5	555	58% (38.3−77.6)			

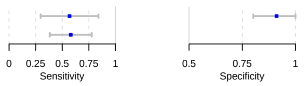

Supplement: S11 Fig — CI, confidence interval. (PDF) [file pmed.1004011.s012.pdf]
