# Supplementary material for: Accuracy of rapid point-of-care antigen-based diagnostics for SARS-CoV-2: An updated systematic review and meta-analysis with meta-regression analyzing influencing factors
Source: PLoS Med. 2022 May 26;19(5):e1004011. doi: 10.1371/journal.pmed.1004011 (PMC9187092; doi:10.1371/journal.pmed.1004011)

S12 Fig. a Funnel plot for all studies

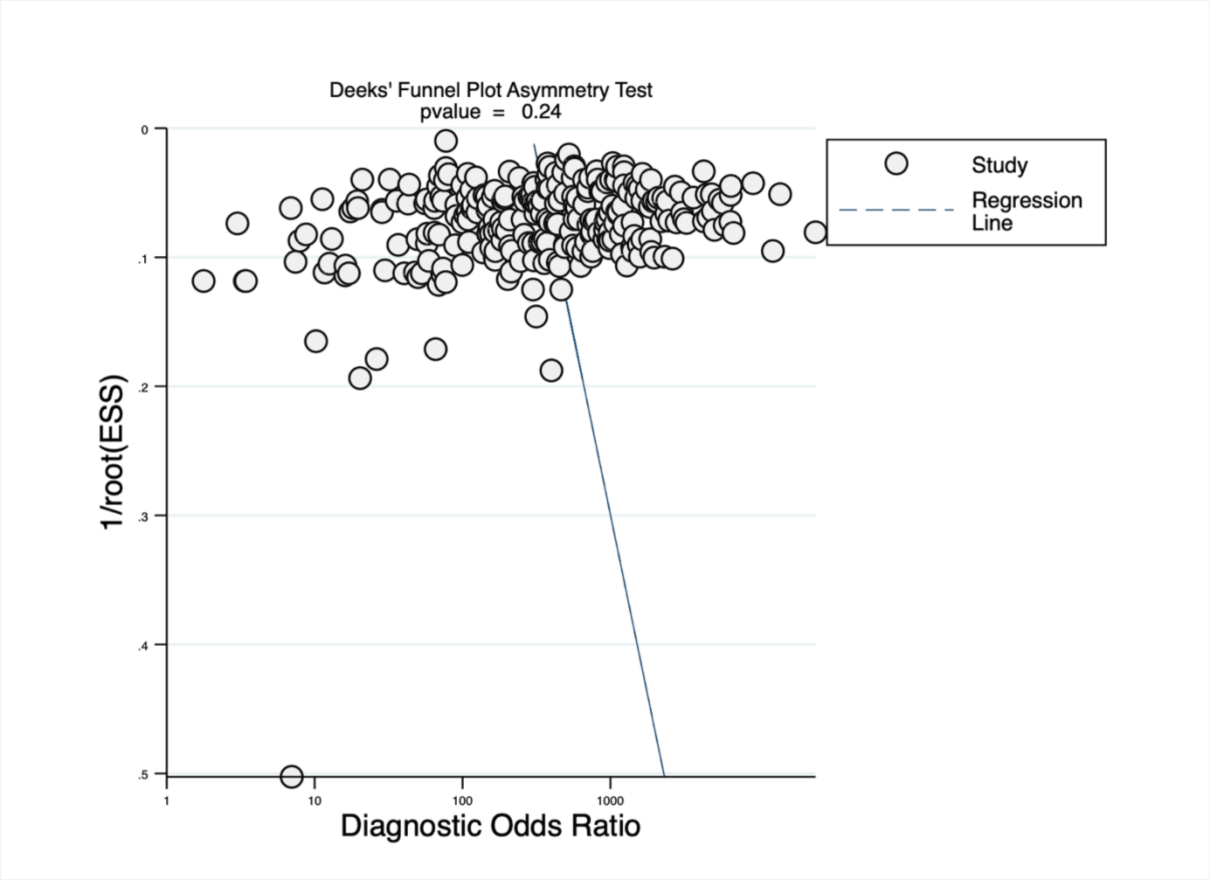

S12 Fig. b Funnel plot for LumiraDx studies

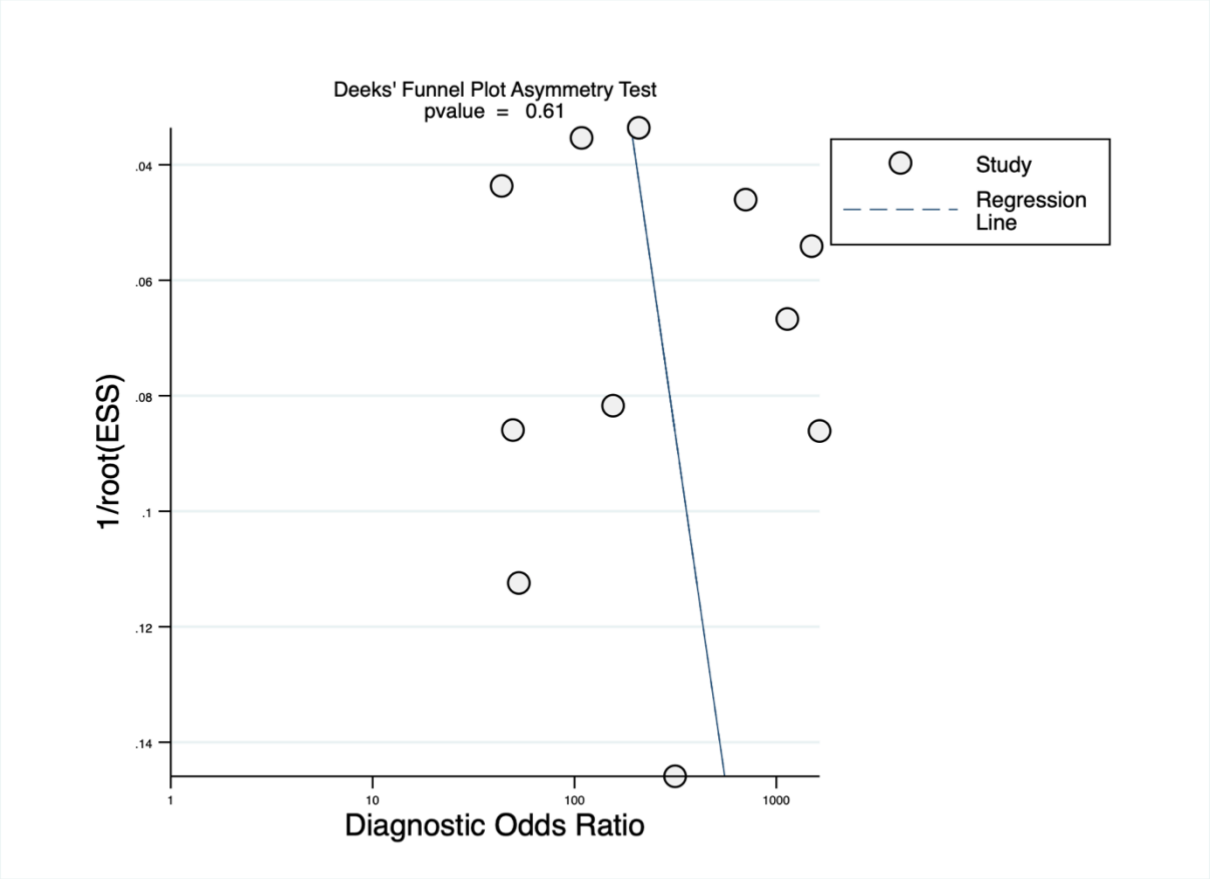

S12 Fig. c Funnel plot for Panbio studies

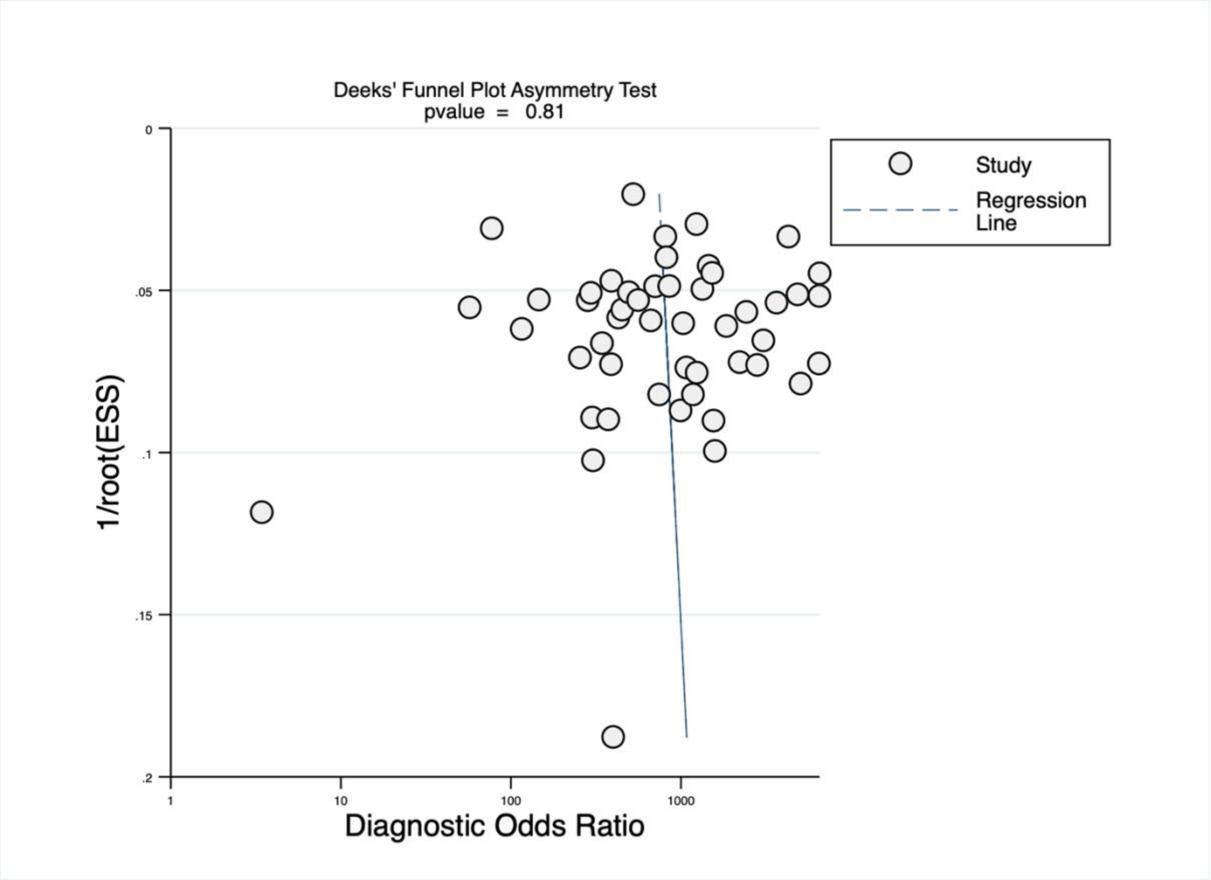

S12 Fig. d Funnel plot for Standard Q studies

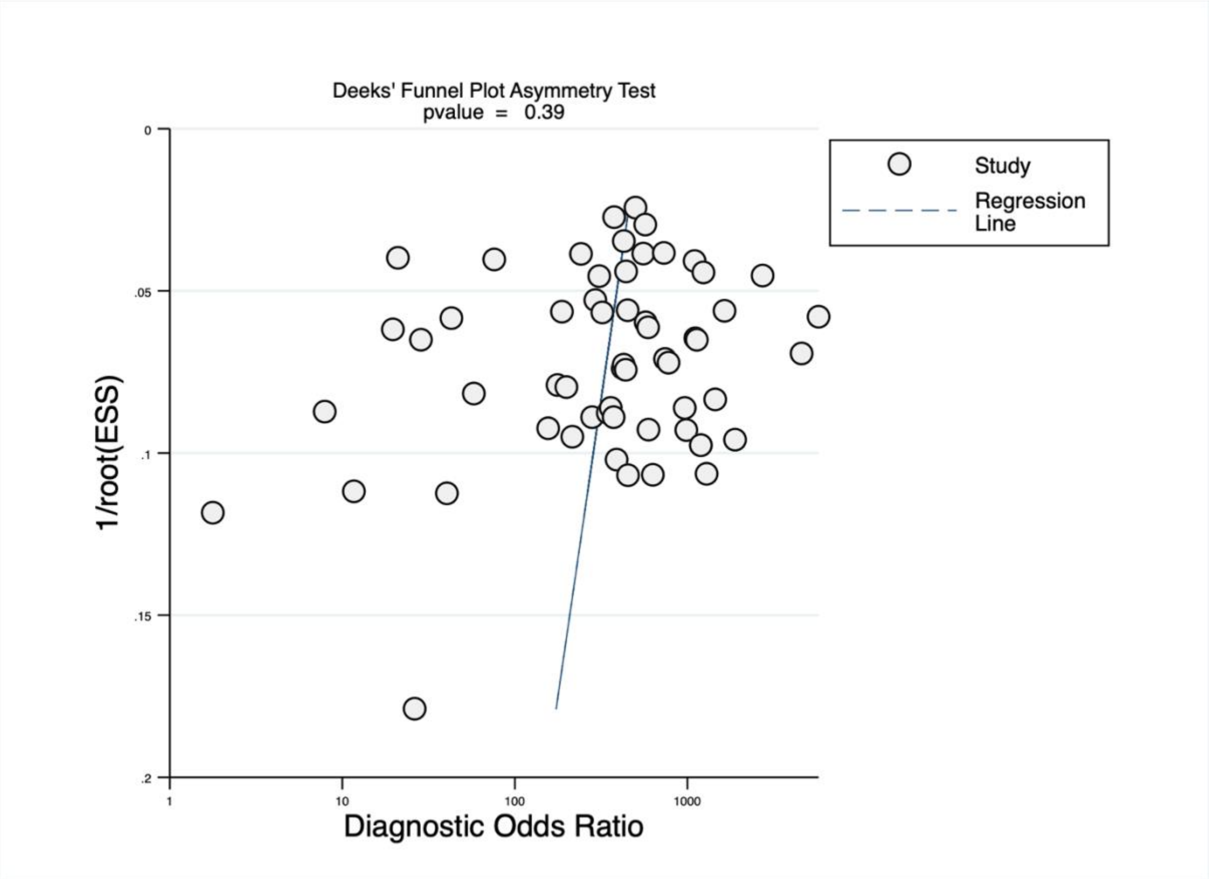

Supplement: S12 Fig — (PDF) [file pmed.1004011.s013.pdf]
