# Supplementary material for: Accuracy of rapid point-of-care antigen-based diagnostics for SARS-CoV-2: An updated systematic review and meta-analysis with meta-regression analyzing influencing factors
Source: PLoS Med. 2022 May 26;19(5):e1004011. doi: 10.1371/journal.pmed.1004011 (PMC9187092; doi:10.1371/journal.pmed.1004011)
Supplement: S2 Text — (DOCX) [file pmed.1004011.s020.docx]

S2 Text. Search strategy.

## Review questions

To assess the accuracy and ease-of-use of marketable antigen point of care diagnostics for SARS-CoV-2 compared to RT-PCR based on manufacturer independent evaluations.

Restriction: start Dezember 2019

## Definitions

P

| SARS-CoV-2 |
| --- |

I

| Antigen nachweisenden Schnelltests Ag RDT |
| --- |

## Strategie

| 1 | P |
| --- | --- |
| 2 | I |
| 3 | 1 AND 2 |

## Searched Databases

- PubMed
- Web of Science Core Collection
- BioRxiv
- MedRxiv

## PubMed

P

| (**"Severe Acute Respiratory Syndrome Coronavirus 2"[Supplementary Concept] OR**  **"COVID-19" [Supplementary Concept] OR**  **"Betacoronavirus"[Mesh] OR**  **"Coronavirus"[Mesh] OR**  covid*[tw] OR  "coronavirus*"[tw] OR  "corona virus*"[tw] OR  ncov*[tw] OR  "n cov*"[tw] OR  sarscov*[tw] OR  "sars cov*"[tw] OR  "2019nCoV*"[tw] OR  "2019 nCoV*"[tw] OR  "sars2*"[tw] OR  "sars 2*"[tw]) |
| --- |

I

| **"Point-of-Care Testing"[Mesh] OR**  Antigen[tw] OR  “Lateral flow”[tw] OR  RDT[tw] OR  (("Point of Care*"[tw] OR  "Bedside*"[tw] OR  Rapid*[tw])  AND  Test*[tw]) |
| --- |

**P**

("Severe Acute Respiratory Syndrome Coronavirus 2"[Supplementary Concept] OR "COVID-19"[Supplementary Concept] OR "Betacoronavirus"[MeSH Terms] OR "Coronavirus"[MeSH Terms] OR "covid*"[Text Word] OR "coronavirus*"[Text Word] OR "corona virus*"[Text Word] OR "ncov*"[Text Word] OR "n cov*"[Text Word] OR "sarscov*"[Text Word] OR "sars cov*"[Text Word] OR "2019ncov*"[Text Word] OR "2019 ncov*"[Text Word] OR "sars2*"[Text Word] OR "sars 2*"[Text Word])

**I**

("Point-of-Care Testing"[MeSH Terms] OR "antigen"[Text Word] OR "lateral flow"[Text Word] OR "RDT"[Text Word] OR (("point of care*"[Text Word] OR "bedside*"[Text Word] OR "rapid*"[Text Word]) AND "test*"[Text Word]))

2019/12/01:2021/08/31[Date - Publication]

## Web of Science Core Collection

P

| "covid*" OR  "coronavirus*" OR  "corona virus*" OR  "ncov*" OR  "n cov*" OR  "sarscov*" OR  "sars cov*" OR  "2019nCoV*" OR  "2019 nCoV*" OR  "sars2*" OR  "sars 2*" |
| --- |

I

| "antigen" OR  "Lateral flow" OR  "RDT" OR  (("Point of Care*" OR  "Bedside*" OR  "Rapid*")  AND  Test*)) |
| --- |

P

TS=("covid*" OR "coronavirus*" OR "corona virus*" OR "ncov*" OR "n cov*" OR "sarscov*" OR "sars cov*" OR "2019nCoV*" OR "2019 nCoV*" OR "sars2*" OR "sars 2*")

I

TS=("antigen" OR "Lateral flow" OR "RDT" OR (("Point of Care*" OR "Bedside*" OR "Rapid*")AND Test*))

Filter (year to date 2021/08/31)

## Bio_MedRxiv

https://europepmc.org/

P

| Covid* OR  Coronavirus* OR  "corona virus*" OR  Ncov* OR  "n cov*" OR  Sarscov* OR  "Sars cov*" OR  2019nCoV* OR  "2019 nCoV*" OR  sars2* OR  "sars 2*" |
| --- |

I

| "Antigen*" OR  "Lateral flow*" OR  "RDT" OR  "Rapid test*" OR  "Bedside*" OR  "Point of Care*" |
| --- |

**P AND I**

(covid* OR Coronavirus* OR "corona virus*" OR ncov* OR "n cov*" OR sarscov* OR "sars cov*" OR 2019nCov* OR "2019 nCov*" OR sars2* OR "sars 2*")

AND

(Antigen OR "Lateral flow" OR RDT OR "Rapid test*" OR Bedside* OR "Point of Care*")

AND

(PUBLISHER:MedRxiv OR PUBLISHER:BioRxiv)

AND

FIRST_PDATE:[2019-12-01 TO 2021-08-31]
