# Supplementary material for: Accuracy of rapid point-of-care antigen-based diagnostics for SARS-CoV-2: An updated systematic review and meta-analysis with meta-regression analyzing influencing factors
Source: PLoS Med. 2022 May 26;19(5):e1004011. doi: 10.1371/journal.pmed.1004011 (PMC9187092; doi:10.1371/journal.pmed.1004011)
Supplement: S3 Text — (DOCX) [file pmed.1004011.s021.docx]

S3 Text. QUDAS-2 assessment interpretation guide.

**Domain 1 Patient Selection:**

Risk of Bias: Could the selection of patients have introduced bias?

• Signaling question 1: Was a consecutive or random sample of patients or specimens enrolled?

We scored ‘yes’ if the study enrolled a consecutive or random sample of eligible patients; ‘no’ if the study selected patients by convenience, and ‘unclear’ if the study did not report the manner of patient selection or unable to tell.

• Signaling question 2: Was a case-control design avoided?

We scored ‘no’ if the study selected samples with a known rt-PCR results. We scored ‘yes’ if the status of the samples was unknown. We scored ‘unclear’ if we could not tell.

• Signaling question 3: Did the study avoid inappropriate exclusions?

We scored ’yes’ to studies which included all participants regardless of symptoms or duration of symptoms. We scored ’no’ if studies excluded participants on the basis of symptoms or duration of symptoms. We scored ’unclear’ if we could not tell.

We considered any studies that included patients based on a previous positive rt-PCR results as monitoring studies and thus judged the applicability of the study population to be of ‘high concern’ (see below).

- Risk of Bias was scored ‘low concern’ if studies score ‘yes’ on all the question, ‘unclear concern’ if questions are answered with ‘yes’ and ‘unclear’, ‘intermediate concern’ if one question is answered with ‘no’, ‘high concern’ if two or more questions are answered with ‘no’.

Applicability: Are there concerns that the included patients and setting do not match the review question?

We were interested in how Ag-RDT performs in patients whose specimens were evaluated as they would be in routine practice. We scored ’low concern’ if the study was conducted in a routine practice setting. We scored ‘high concern’ if Ag-RDT were evaluated for end of quarantine evaluation or monitoring. We scored ‘unclear’ if we could not tell.

**Domain 2: Index Test**

Risk of Bias: Could the conduct or interpretation of the index test have introduced bias?

• Signaling question 1: Were the index test results interpreted with knowledge of the results of the reference standard?

We answered ’yes’ if the study interpreted the result of Ag-RDT blinded to the result of the reference standard or for studies in which Ag-RDT was performed on fresh specimens, since reference standard results would be unavailable at the time of test interpretation. We answered ’no’ if the study did not interpret the result of Ag-RDT blinded to the result of the reference standard. We answered ’unclear’ if stored specimens were tested or we could not tell if the index test results were interpreted without knowledge of the reference standard results.

• Signaling question 2: If a threshold was used, was it prespecified?

We answered ’yes’ if the threshold was prespecified or if the tests was performed by IFU. We scored ’no’ if the threshold was not prespecified, and ’unclear’ if we could not determine if the threshold was prespecified or not.

- Risk of Bias was scored ‘low concern’ if studies score ‘yes’ on all the question, ‘unclear concern’ if questions are answered with ‘yes’ and ‘unclear’, ‘intermediate concern’ if one question is answered with ‘no’, ‘high concern’ if two or more questions are answered with ‘no’.

Applicability: Are there concerns that the index test, its conduct, or its interpretation differ from the review question? If index test methods vary from those specified in the review question, concerns about applicability may exist. We judged ’high concern’ if the test procedure was inconsistent with the manufacturer recommendations, ’low concern’ if the test procedure was consistent with the manufacturer recommendations, and ’unclear concern’ if we could not tell.

**Domain 3: Reference Standard**

Risk of Bias: Could the reference standard, its conduct, or its interpretation have introduced bias?

• Signaling question 1: Is the reference standard likely to correctly classify the target condition?

Viral culture is considered the gold standard for SARS-CoV-2 detection. Since viral culture is available in research settings only, NAAT is the considered routine standard for SARS-CoV-2 testing. However, the accuracy of this reference standard is not 100%, especially late in the disease, where it varies widely across the different non-respiratory samples and may detect non-viable virus Still, given that viral loads measured in NAATs correlate well with Antigen, we scored ‘yes’ for all studies using a NAAT as reference standard.

• Signaling question 2: Were the reference standard results interpreted without knowledge of the results of the index test?

We scored ‘yes’ if the test was performed ahead of the Ag-RDT or blinding was specifically reported. We scored ‘unclear’ if we could not tell.

- Risk of Bias is scored ‘low concern’ if studies score ‘yes’ on all the question, ‘unclear concern’ if questions are answered with ‘yes’ and ‘unclear’, ‘intermediate concern’ if one question is answered with ‘no’, ‘high concern’ if two or more questions are answered with ‘no’.

Applicability: Are there concerns that the target condition as defined by the reference standard does not match the question?

We judged applicability to be of ‘low concern’ for all studies.

**Domain 4: Flow and Timing**

Risk of Bias: Could the patient flow have introduced bias?

• Signaling question 1: Was there an appropriate interval between the index test and reference standard?

We expected specimens for Ag-RDT and the reference standards to be obtained at the same time and answered ’yes’ for all studies that meet these criteria. We answered ‘unclear’ if we could not tell.

• Signaling question 2: Did all patients receive the same reference standard?

We answered this question ‘yes’ for all studies that used the same rt-PCR for all samples and ‘no’ if the samples were analyzed by different types of rt-PCR. We scored ‘unclear’ if we could not tell the used rt-PCR.

• Signaling question 3: Were all patients included in the analysis?

We determined the answer to this question by comparing the stated population size with the number of samples included in the two-by-two tables. We answered ’yes’ if the whole population was included in the analysis or any excluded samples were reasoned for. We answered ‘no’ if samples were excluded without a given reason. We answered ‘unclear’ if we could not tell.

- Risk of Bias is scored ‘low concern’ if studies score ‘yes’ on all the question, ‘unclear concern’ if questions are answered with ‘yes’ and ‘unclear’, ‘intermediate concern’ if one question is answered with ‘no’, ‘high concern’ if two or more questions are answered with ‘no’.
