# Supplementary material for: Accuracy of rapid point-of-care antigen-based diagnostics for SARS-CoV-2: An updated systematic review and meta-analysis with meta-regression analyzing influencing factors
Source: PLoS Med. 2022 May 26;19(5):e1004011. doi: 10.1371/journal.pmed.1004011 (PMC9187092; doi:10.1371/journal.pmed.1004011)
Supplement: S5 Text — (DOCX) [file pmed.1004011.s023.docx]

**S5 Text – Studies excluded (number of studies: 263)**

Ag-RDT not commercially available (47)

1. Azzi L, Baj A, Alberio T, Lualdi M, Veronesi G, Carcano G, et al. Rapid Salivary Test suitable for a mass screening program to detect SARS-CoV-2: A diagnostic accuracy study. Journal of Infection, 2020; 81(3):E75-E78. DOI:10.1016/j.jinf.2020.06.042.

2. Barauna VG, Singh MN, Barbosa LL, Marcarini WD, Vassallo PF, Mill JG, et al. Ultrarapid On-Site Detection of SARS-CoV-2 Infection Using Simple ATR-FTIR Spectroscopy and an Analysis Algorithm: High Sensitivity and Specificity. Analytical Chemistry, 2021; 93(5):2950–2958. DOI:10.1021/acs.analchem.0c04608.

3. Barlev-Gross M, Weiss S, Ben-Shmuel A, Sittner A, Eden K, Mazuz N, et al. Spike vs nucleocapsid SARS-CoV-2 antigen detection: application in nasopharyngeal swab specimens. Analytical and Bioanalytical Chemistry, 2021; 413:3501–3510. DOI:10.1101/2021.03.08.21253148.

4. Barlev-Gross M, Weiss S, Paran N, Yahalom-Ronen Y, Israeli O, Nemet I, et al. Sensitive immunodetection of SARS-CoV-2 variants-of-concern 501Y.V2 and 501Y.V1. The Journal of Infectious Diseases, 2021; 224(4):616-619. DOI:10.1093/infdis/jiab278.

5. Cardozo KHM, Lebkuchen A, Okai GG, Schuch RA, Viana LG, Olive AN, et al. Establishing a mass spectrometry-based system for rapid detection of SARS-CoV-2 in large clinical sample cohorts. Nature Communications, 2020; 11(1):6201. DOI:10.1038/s41467-020-19925-0.

6. Cazares LH, Chaerkady R, Samuel Weng SH, Boo CC, Cimbro R, Hsu HE, et al. Development of a Parallel Reaction Monitoring Mass Spectrometry Assay for the Detection of SARS-CoV-2 Spike Glycoprotein and Nucleoprotein. Analytical Chemistry, 2020; 92(20):13813-13821. DOI:10.1021/acs.analchem.0c02288.

7. Chen H, Li Z, Feng S, Wang A, Richard-Greenblatt M, Hutson E, et al. Femtomolar SARS-CoV-2 Antigen Detection Using the Microbubbling Digital Assay with Smartphone Readout Enables Antigen Burden Quantitation and Dynamics Tracking. medRxiv [Preprint]; published March 26, 2021. DOI:10.1101/2021.03.17.21253847.

8. Chivte P, LaCasse Z, Seethi VD, Bharti P, Bland J, Kadkol S, et al. MALDI-ToF Protein Profiling as Potential Rapid Diagnostic Platform for COVID-19. Journal of Mass Spectrometry & Advances in the Clinical Lab, 2021; 21:31-41. DOI:10.1101/2021.05.26.21257798.

9. Conzelmann C, Gilg A, Groß R, Schütz D, Preising N, Ständker L, et al. An enzyme-based immunodetection assay to quantify SARS-CoV-2 infection. Antiviral Research, 2020; 181:104882. DOI:10.1016/j.antiviral.2020.104882.

10. de Lima LF, Ferreira AL, Torres MDT, de Araujo WR, de la Fuente-Nunez C. Minute-scale detection of SARS-CoV-2 using a low-cost biosensor composed of pencil graphite electrodes. Proceedings of the National Academy of Sciences of the United States of America, 2021; 118(30):e2106724118. DOI:10.1073/pnas.2106724118.

11. Di Domenico M, De Rosa A, Boccellino M. Detection of SARS-COV-2 Proteins Using an ELISA Test. Diagnostics, 2021; 11(4). DOI:10.3390/diagnostics11040698.

12. Diao B, Wen K, Zhang J, Chen J, Han C, Chen Y, et al. Accuracy of a nucleocapsid protein antigen rapid test in the diagnosis of SARS-CoV-2 infection. Clinical Microbiology and Infection, 2020; 27(2):289.e1-289.e4. DOI:10.1016/j.cmi.2020.09.057.

13. Ducrest PJ. Development and Evaluation of a new Swiss Made SARS-CoV-2 antigen-detecting rapid test. medRxiv [Preprint]; published March 26, 2021. DOI:10.1101/2021.03.25.21252280.

14. Eissa S, Alhadrami HA, Al-Mozaini M, Hassan AM, Zourob M. Voltammetric-based immunosensor for the detection of SARS-CoV-2 nucleocapsid antigen. Microchimica Acta, 2021; 188(6):199. DOI:10.1007/s00604-021-04867-1.

15. Funabashi R, Miyakawa K, Yamaoka Y, Yoshimura S, Yamane S, Jeremiah SS, et al. Development of highly sensitive and rapid antigen detection assay for diagnosis of COVID-19 utilizing optical waveguide immunosensor. Journal of Molecular Cell Biology, 2021; Online ahead of print. DOI:10.1093/jmcb/mjab037.

16. Garza K, Silva AAR, Rosa J, Keating M, Povilaitis S, Spradlin M, et al. Rapid Screening of COVID-19 Disease Directly from Clinical Nasopharyngeal Swabs using the MasSpec Pen Technology. Analytical Chemistry, 2021; 93:12582-12593. DOI:10.1101/2021.05.14.21257006.

17. Grant BD, Anderson CE, Williford JR, Alonzo LF, Glukhova VA, Boyle DS, et al. SARS-CoV-2 Coronavirus Nucleocapsid Antigen-Detecting Half-Strip Lateral Flow Assay Toward the Development of Point of Care Tests Using Commercially Available Reagents. Analytical Chemistry, 2020; 92(16):11305-11309. DOI:10.1021/acs.analchem.0c01975.

18. Gupta A, Anand A, Jain N, Goswami S, Ananthraj A, Patil S, et al. A novel G-quadruplex aptamer-based spike trimeric antigen test for the detection of SARS-CoV-2. Molecular Therapy - Nucleic Acids, 2021; 26:321-332. DOI:10.1016/j.omtn.2021.06.014.

19. Hristov D, Rijal H, Gomez-Marquez J, Hamad-Schifferli K. Developing a Paper-Based Antigen Assay to Differentiate between Coronaviruses and SARS-CoV-2 Spike Variants. Analytical Chemistry, 2021; 93(22):7825-7832. DOI:10.1021/acs.analchem.0c05438.

20. Huang L, Ding L, Zhou J, Chen S, Chen F, Zhao C, et al. One-step rapid quantification of SARS-CoV-2 virus particles via low-cost nanoplasmonic sensors in generic microplate reader and point-of-care device. Biosensors and Bioelectronics, 2021; 171:112685. DOI:10.1016/j.bios.2020.112685.

21. Kim S, Yee E, Miller EA, Hao Y, Tay DMY, Sung KJ, et al. Developing a SARS-CoV-2 Antigen Test Using Engineered Affinity Proteins. ACS Applied Materials & Interfaces, 2021; 13(33):38990–39002. DOI:10.1021/acsami.1c08174.

22. Kyosei Y, Namba M, Yamura S, Takeuchi R, Aoki N, Nakaishi K, et al. Proposal of De Novo Antigen Test for COVID-19: Ultrasensitive Detection of Spike Proteins of SARS-CoV-2. Diagnostics, 2020; 10(8):594. DOI:10.3390/diagnostics10080594.

23. Kyosei Y, Namba M, Yamura S, Watabe S, Yoshimura T, Sasaki T, et al. Improved detection sensitivity of an antigen test for SARS-CoV-2 nucleocapsid proteins with thio-NAD cycling. Biological and Pharmaceutical Bulletin, 2021; 44(9):1332-1336. DOI:10.1248/bpb.b21-00387.

24. Lee JH, Choi M, Jung Y, Lee SK, Lee CS, Kim J, et al. A novel rapid detection for SARS-CoV-2 spike 1 antigens using human angiotensin converting enzyme 2 (ACE2). Biosensors and Bioelectronics, 2021; 171:112715. DOI:10.1016/j.bios.2020.112715.

25. Lee L, Liu F, Chen Y, Roma G. Quantitative and Ultrasensitive In-situ Immunoassay Technology for SARS-CoV-2 Detection in Saliva. Research Square [Preprint]; published January 18, 2021. DOI:10.21203/rs.3.rs-138025/v1.

26. Li Y, Peng Z, Holl NJ, Hassan MR, Pappas JM, Wei C, et al. MXene-Graphene Field-Effect Transistor Sensing of Influenza Virus and SARS-CoV-2. ACS Omega, 2021; 6(10):6643-6653. DOI:10.1021/acsomega.0c05421.

27. Liu D, Ju C, Han C, Shi R, Chen X, Duan D, et al. Nanozyme chemiluminescence paper test for rapid and sensitive detection of SARS-CoV-2 antigen. Biosensors & Bioelectronics, 2020; 173:112817. DOI:10.1101/2020.06.05.131748.

28. Mahari S, Roberts A, Shahdeo D, Gandhi S. eCovSens-Ultrasensitive Novel In-House Built Printed Circuit Board Based Electrochemical Device for Rapid Detection of nCovid-19. bioRxiv [Preprint]; published May 11, 2020. DOI:10.1101/2020.04.24.059204.

29. Nash B, Badea A, Reddy A, Bosch M, Salcedo N, Gomez AR, et al. Validating and modeling the impact of high-frequency rapid antigen screening on COVID-19 spread and outcomes. medRxiv [Preprint]; published May 13, 2021. DOI:10.1101/2020.09.01.20184713.

30. Pollock N, Savage T, Wardell H, Lee R, Mathew A, Stengelin M, et al. Correlation of SARS-CoV-2 Nucleocapsid Antigen and RNA Concentrations in Nasopharyngeal Samples from Children and Adults Using an Ultrasensitive and Quantitative Antigen Assay. Journal of Clinical Microbiology, 2020; 59:e03077-20. DOI:10.1128/JCM.03077-20.

31. Renuse S, Vanderboom P, Maus A, Kemp J, Gurtner K, Madugundu A, et al. Development of mass spectrometry-based targeted assay for direct detection of novel SARS-CoV-2 coronavirus from clinical specimens. medRxiv [Preprint]; published August 06, 2020. DOI:10.1101/2020.08.05.20168948.

32. Rusanen J, Kareinen L, Szirovicza L, Uğurlu H, Levanov L, Jääskeläinen A, et al. A Generic, Scalable, and Rapid Time-Resolved Förster Resonance Energy Transfer-Based Assay for Antigen Detection-SARS-CoV-2 as a Proof of Concept. mBio, 2021; 12(3):e00902-21. DOI:10.1128/mBio.00902-21.

33. Seo G, Lee G, Kim MJ, Baek SH, Choi M, Ku KB, et al. Rapid Detection of COVID-19 Causative Virus (SARS-CoV-2) in Human Nasopharyngeal Swab Specimens Using Field-Effect Transistor-Based Biosensor. American Chemical Society, 2020; 14(4):5135-5142. DOI:10.1021/acsnano.0c02823.

34. Shao W, Shurin MR, Wheeler SE, He X, Star A. Rapid Detection of SARS-CoV-2 Antigens Using High-Purity Semiconducting Single-Walled Carbon Nanotube-Based Field-Effect Transistors. ACS Applied Materials & Interfaces, 2021; 13(8):10321–10327. DOI:10.1021/acsami.0c22589.

35. Singh NK, Ray P, Carlin AF, Morgan SC, Magallanes C, Laurent LC, et al. Dataset on optimization and development of a point-of-care glucometer-based SARS-CoV-2 detection assay using aptamers. Data Brief, 2021; 38:107278. DOI:10.1016/j.dib.2021.107278.

36. Singh P, Chakraborty R, Marwal R, Radhakrishan VS, Bhaskar AK, Vashisht H, et al. A rapid and sensitive method to detect SARS-CoV-2 virus using targeted-mass spectrometry. Journal of Proteins and Proteomics, 2020:1-7. DOI:10.1007/s42485-020-00044-9.

37. Stambaugh A, Parks JW, Stott MA, Meena GG, Hawkins AR, Schmidt H. Optofluidic multiplex detection of single SARS-CoV-2 and influenza A antigens using a novel bright fluorescent probe assay. Proceedings of the National Academy of Sciences of the United States of America, 2021; 118(20). DOI:10.1073/pnas.2103480118.

38. Torrente-Rodríguez RM, Lukas H, Tu J, Min J, Yang Y, Xu C, et al. SARS-CoV-2 RapidPlex: A Graphene-based Multiplexed Telemedicine Platform for Rapid and Low-Cost COVID-19 Diagnosis and Monitoring. Matter, 2020; 3(6):1981–1998. DOI:10.1016/j.matt.2020.09.027.

39. Vadlamani BS, Uppal T, Verma S, Misra M. Functionalized TiO2 nanotube-based Electrochemical Biosensor for Rapid Detection of SARS-CoV-2. Sensors, 2020; 20(20):5871. DOI:10.1101/2020.09.07.20190173.

40. Wang C, Cheng X, Liu L, Zhang X, Yang X, Zheng S, et al. Ultrasensitive and Simultaneous Detection of Two Specific SARS-CoV-2 Antigens in Human Specimens Using Direct/Enrichment Dual-Mode Fluorescence Lateral Flow Immunoassay. ACS Applied Materials & Interfaces, 2021; 13(34):40342-40353. DOI:10.1021/acsami.1c11461.

41. Wang C, Wang C, Qiu J, Gao J, Liu H, Zhang Y, et al. Ultrasensitive, high-throughput, and rapid simultaneous detection of SARS-CoV-2 antigens and IgG/IgM antibodies within 10 min through an immunoassay biochip. Microchimica Acta, 2021; 188(8):262. DOI:10.1007/s00604-021-04896-w.

42. Wang H, Hogan CA, Verghese M, Solis D, Sibai M, Huang C, et al. Ultra-sensitive Severe Acute Respiratory Syndrome Coronavirus 2 (SARS-CoV-2) Antigen Detection for the Diagnosis of Coronavirus Disease 2019 (COVID-19) in Upper Respiratory Samples. Clinical Infectious Diseases, 2021; Online ahead of print. DOI:10.1093/cid/ciab063.

43. Yakoh A, Pimpitak U, Rengpipat S, Hirankarn N, Chailapakul O, Chaiyo S. Paper-based electrochemical biosensor for diagnosing COVID-19: Detection of SARS-CoV-2 antibodies and antigen. Biosensors & Bioelectronics, 2021; 176:112912. DOI:10.1016/j.bios.2020.112912.

44. Zakashansky J, Imamura A, Salgado D, Romero Mercieca H, Aguas RFL, Lao A, et al. Detection of the SARS-CoV-2 spike protein in saliva with Shrinky-Dink electrodes. Analytical Methods, 2020; 13(7):874-883. DOI:10.1101/2020.11.14.20231811.

45. Zhang CY, Zhou L, Du K, Zhang Y, Wang J, Chen LJ, et al. Foundation and Clinical Evaluation of a New Method for Detecting SARS-CoV-2 Antigen by Fluorescent Microsphere Immunochromatography. Frontiers in Cellular and Infection Microbiology, 2020; 10:553837. DOI:10.3389/fcimb.2020.553837.

46. Su B, Yin J, Lin X, Zhang T, Yao X, Xu Y, et al. Quantification of SARS-CoV-2 antigen levels in the blood of patients with COVID-19. Science China Life Science, 2021; 64(7):1193-1196. DOI:10.1007/s11427-020-1830-8.

47. Bernard M, Cosentino G, Pieri L, Zachary P, Buser M, Kbaier L, et al. Retrospective analysis of the performance of the SARS-CoV-2 rapid antigen detection test compared to the reference RT-PCR test. Annales De Biologie Clinique, 2021; 79(2):168-175. DOI:10.1684/abc.2021.1641.

Analytical study (13)

1. Bekliz M, Adea K, Essaidi-Laziosi M, Sacks JA, Escadafal C, Kaiser L, et al. SARS-CoV-2 rapid diagnostic tests for emerging variants. Lancet Microbe, 2021; 2(8):e351. DOI:10.1016/s2666-5247(21)00147-6.

2. Bourassa L, Perchetti G, Phung Q, Lin M, Mills M, Roychoudhury P, et al. A SARS-CoV-2 Nucleocapsid Variant that Affects Antigen Test Performance. Journal of Clinical Virology, 2021; 141:104900. DOI:10.1101/2021.05.05.21256527.

3. Corman V, Haage V, Bleicker T, Schmidt ML, Muehlemann B, Zuchowski M, et al. Comparison of seven commercial SARS-CoV-2 rapid Point-of-Care Antigen tests. The Lancet Microbe, 2020; 2(7):E311-E319. DOI:10.1016/S2666-5247(21)00056-2.

4. Cubas Atienzar A, Kontogianni K, Edwards T, Wooding D, Buist K, Thompson C, et al. Limit of detection in different matrices of nineteen commercially available rapid antigen tests for the detection of SARS-CoV-2. Science Reports, 2021; 11:18313. DOI:10.1038/s41598-021-97489-9.

5. Fiedler M, Holtkamp C, Dittmer U, Anastasiou OE. Performance of the LIAISON(R) SARS-CoV-2 Antigen Assay vs. SARS-CoV-2-RT-PCR. Pathogens, 2021; 10(6):658. DOI:10.3390/pathogens10060658.

6. Haage V, Ferreira de Oliveira-Filho E, Moreira-Soto A, Kühne A, Fischer C, Sacks JA, et al. Impaired performance of SARS-CoV-2 antigen-detecting rapid diagnostic tests at elevated and low temperatures. Journal of Clinical Virology, 2021; 138:104796. DOI:10.1016/j.jcv.2021.104796.

7. Jungnick S, Hobmaier B, Mautner L, Hoyos M, Haase M, Baiker A, et al. Detection of the new SARS-CoV-2 variants of concern B.1.1.7 and B.1.351 in five SARS-CoV-2 rapid antigen tests (RATs), Germany, March 2021. Euro Surveillance, 2021; 26(16):2100413. DOI:10.2807/1560-7917.Es.2021.26.16.2100413.

8. Kontogianni K, Cubas-Atienzar AI, Wooding D, Buist K, Thompson CR, Williams CT, et al. Lateral flow antigen tests can sensitively detect live cultured virus of the SARS-CoV-2 B1.1.7 lineage. Journal of Infection, 2021; 83(1):e1-e4. DOI:10.1016/j.jinf.2021.05.033.

9. Mak GC, Lau SS, Wong KK, Chow NL, Lau CS, Lam ET, et al. Analytical sensitivity and clinical sensitivity of the three rapid antigen detection kits for detection of SARS-CoV-2 virus. Journal of Clinical Virology, 2020; 133:104684. DOI:10.1016/j.jcv.2020.104684.

10. Mak GCK, Cheng PKC, Lau SSY, Wong KKY, Lau CS, Lam ETK, et al. Evaluation of rapid antigen test for detection of SARS-CoV-2 virus. Journal of Clinical Virology, 2020; 129:104500. DOI:10.1016/j.jcv.2020.104500.

11. Mak GCK, Lau SSY, Wong KKY, Chow NLS, Lau CS, Lam ETK, et al. Evaluation of rapid antigen detection kit from the WHO Emergency Use List for detecting SARS-CoV-2. Journal of Clinical Virology, 2021; 134:104712. DOI:10.1016/j.jcv.2020.104712.

12. Perchetti GA, Huang ML, Mills MG, Jerome KR, Greninger AL. Analytical Sensitivity of the Abbott BinaxNOW COVID-19 Ag CARD. Journal of Clinical Microbiology, 2020; 59(3):e02880-20. DOI:10.1128/jcm.02880-20.

13. Rodgers M, Batra R, Snell L, Daghfal D, Roth R, Huang S, et al. Detection of SARS-CoV-2 variants by Abbott molecular, antigen, and serological tests. medRxiv [Preprint]; published April 26, 2021. DOI:10.1101/2021.04.24.21256045.

Did not assess an Ag-RDT (25)

1. Adamoski D, de Oliveira JC, Bonatto AC, Wassem R, Nogueira MB, Raboni SM, et al. Large-scale screening of asymptomatic for SARS-CoV-2 variants of concern and rapid P.1 takeover, Curitiba, Brazil. Emerging Infectious Diseases, 2021; 12. DOI:10.1101/2021.06.18.21258649.

2. Apostolou T, Kyritsi M, Vontas A, Loizou K, Hadjilouka A, Speletas M, et al. Development and performance characteristics evaluation of a new Bioelectric Recognition Assay (BERA) method for rapid Sars-CoV-2 detection in clinical samples. Journal of Virological Methods, 2021; 293:114166. DOI:10.1016/j.jviromet.2021.114166.

3. Arnaout R, Lee RA, Lee GR, Callahan C, Cheng A, Yen CF, et al. The Limit of Detection Matters: The Case for Benchmarking Severe Acute Respiratory Syndrome Coronavirus 2 Testing. Clinical Infectious Diseases, 2021; ciaa1382. DOI:10.1093/cid/ciaa1382.

4. Ceci A, Muñoz-Ballester C, Tegge AN, Brown KL, Umans RA, Michel FM, et al. Development and implementation of a scalable and versatile test for COVID-19 diagnostics in rural communities. Nature Communications, 2021; 12(1):4400. DOI:10.1038/s41467-021-24552-4.

5. Charpentier C, Pellissier G, Ichou H, Ferré VM, Larfi I, Phung BC, et al. Contribution of rapid lateral flow assays from capillary blood specimens to the diagnosis of COVID-19 in symptomatic healthcare workers: a pilot study in a university hospital, Paris, France. Diagnostic Microbiology and Infectious Disease, 2021; 101(2):115430. DOI:10.1016/j.diagmicrobio.2021.115430.

6. Cheah PK, Ongkili DF, Zaharuddin FS, Hashim MI, Ho CV, Lee HG, et al. Discrepancy in Screening Performances of Different Rapid Test Kits for SARS-CoV-2; a Letter to Editor. Archives of Academic Emergency Medicine, 2021; 9(1):e9. DOI:10.22037/aaem.v9i1.1045.

7. Craney A, Petrik D, Suhku A, Qiu Y, Racine-Brzostek S, Rennert H, et al. Performance Evaluation of the MatMaCorp COVID-19 2SF Assay for the Detection of SARS-CoV-2 from Nasopharyngeal Swabs. Microbiology Spectrum, 2021; 9(1):e0008321. DOI:10.1128/Spectrum.00083-21.

8. Doron S, Ingalls R, Beauchamp A, Boehm J, Boucher H, Chow L, et al. Weekly SARS-CoV-2 screening of asymptomatic students and staff to guide and evaluate strategies for safer in-person learning. medRxiv [Preprint]; published March 22, 2021. DOI:10.1101/2021.03.20.21253976.

9. Ebanks D, Faustini S, Shields A, Parry H, Moss P, Plant T, et al. Cross reactivity of serological response to SARS-CoV-2 vaccination with viral variants of concern detected by lateral flow immunoassays. Journal of Infection, 2021; 83(4):E18-E20. DOI:10.1016/j.jinf.2021.07.020.

10. Ekelund O, Ekblom K, Somajo S, Pattison-Granberg J, Olsson K, Petersson A. High-throughput immunoassays for SARS-CoV-2-considerable differences in performance when comparing three methods. Infectious Diseases, 2021; 53(10):805-810. DOI:10.1080/23744235.2021.1931434.

11. Grossi E, Agnoli B, Baldini M, Illari S, Bonini R, Scagnelli G. Universal Sars-Cov-2 Screening in Pregnant Women: Experience from the Italian Epidemic Outbreak. Acta Biomed, 2021; 92(S2):e2021001. DOI:10.23750/abm.v92iS2.11320.

12. Hicks SM, Pohl K, Neeman T, McNamara HA, Parsons KM, He JS, et al. A Dual-Antigen Enzyme-Linked Immunosorbent Assay Allows the Assessment of Severe Acute Respiratory Syndrome Coronavirus 2 Antibody Seroprevalence in a Low-Transmission Setting. Journal of Infectious Diseases, 2021; 223(1):10-14. DOI:10.1093/infdis/jiaa623.

13. Houston H, Deas G, Naik S, Shah K, Patel S, Greca Dottori M, et al. Utility of the FebriDx point-of-care assay in supporting a triage algorithm for medical admissions with possible COVID-19: an observational cohort study. BMJ Open, 2021; 11(8):e049179. DOI:10.1136/bmjopen-2021-049179.

14. Jain S, Jonasson JO, Pauphilet J, Flower B, Moshe M, Fontana G, et al. A new combination testing methodology to identify accurate and economical point-of-care testing strategies. medRxiv [Preprint]; published June 16, 2021. DOI:10.1101/2021.06.15.21257351.

15. Kiyasu Y, Akashi Y, Sugiyama A, Takeuchi Y, Notake S, Naito A, et al. A prospective evaluation of the analytical performance of GENECUBE HQ SARS-CoV-2 and GENECUBE FLU A/B. Molecular Diagnosis & Therapy, 2021; 25:495-504. DOI:10.1101/2021.02.24.21252337.

16. Lagi F, Trevisan S, Piccica M, Graziani L, Basile G, Mencarini J, et al. Use of the FebriDx point-of-care test for the exclusion of SARS-CoV-2 diagnosis in a population with acute respiratory infection during the second (COVID-19) wave in Italy. International Journal of Infectious Diseases, 2021; 108:231-236. DOI:10.1016/j.ijid.2021.04.065.

17. Latiano A, Tavano F, Panza A, Palmieri O, Niro GA, Andriulli N, et al. False Positive Results Of IgM/IgG antibodies against antigen of the SARS-CoV-2 in sera stored before the 2020 Endemia in Italy. International Journal of Infectious Diseases, 2020; 104:159-163. DOI:10.1016/j.ijid.2020.12.067.

18. Li J, Hu X, Wang X, Yang J, Zhang L, Deng Q, et al. A novel One-pot rapid diagnostic technology for COVID-19. Analytica Chimica Acta, 2021; 1154:338310. DOI:10.1016/j.aca.2021.338310.

19. Marsic T, Ali Z, Tehseen M, Mahas A, Hamdan S, Mahfouz M. Vigilant: An Engineered VirD2-Cas9 Complex for Lateral Flow Assay-Based Detection of SARS-CoV2. Nano Letters, 2021; 21(8):3596–3603. DOI:10.1021/acs.nanolett.1c00612.

20. Ong DSY, Koeleman JGM, Vaessen N, Breijer S, Paltansing S, de Man P. Rapid screening method for the detection of SARS-CoV-2 variants of concern: Rapid screening of SARS-CoV-2 variants of concern. Journal of Clinical Virology, 2021; 141:104903. DOI:10.1016/j.jcv.2021.104903.

21. Otake S, Miyamoto S, Mori A, Iwamoto T, Kasai M. False-positive results in SARS-CoV-2 antigen test with rhinovirus-A infection. Pediatrics International, 2021; 63:1135-1137. DOI:10.1111/ped.14582.

22. Schleicher T, Spenlinhauer T, Amadei M, Dasch N, Gordon J, Macleod G, et al. Development of a Multiplexed Synthetic Control for Rapid Detection of SARS-CoV-2 and Other Respiratory Pathogens Using a Nucleic Acid Syndromic Testing Panel. Journal of Molecular Diagnostics, 2020; 22(11):S37-S37.

23. Scotta MC, de David CN, Varela FH, Sartor ITS, Polese-Bonatto M, Fernandes IR, et al. Low performance of a SARS-CoV-2 point-of-care lateral flow immunoassay in symptomatic children during the pandemic. Journal of Pediatrics, 2021; Online ahead of print. DOI:10.1016/j.jped.2021.04.010.

24. Wood BR, Kochan K, Bedolla DE, Salazar-Quiroz N, Grimley S, Perez-Guaita D, et al. Infrared based saliva screening test for COVID-19. Angewandte Chemie International Edition, 2021; 60(31):17102–17107. DOI:10.1002/anie.202104453.

25. Zou M, Su F, Zhang R, Jiang X, Xiao H, Yan X, et al. Rapid Point-of-Care Testing for SARS-CoV-2 Virus Nucleic Acid Detection by an Isothermal and Nonenzymatic Signal Amplification System Coupled with a Lateral Flow Immunoassay Strip. Sensors and Actuators B: Chemical, 2021; 342:129899. DOI:10.1016/j.snb.2021.129899.

Duplication of data (4)

1. Ikeda M, Imai K, Tabata S, Miyoshi K, Mizuno T, Murahara N, et al. Clinical evaluation of self-collected saliva by RT-qPCR, direct RT-qPCR, RT-LAMP, and a rapid antigen test to diagnose COVID-19. Journal of Clinical Microbiology, 2020; 59(8):e01438-20. DOI:10.1101/2020.06.06.20124123.

2. Lindner A, Krüger L, Nikolai O, Klein JAF, Rössig H, Schnitzler P, et al. SARS-CoV-2 variant of concern B.1.1.7: diagnostic accuracy of three antigen-detecting rapid tests. medRxiv [Preprint]; published June 15, 2021. DOI:10.1101/2021.06.15.21258502.

3. Masiá M, Fernández-González M, Sánchez M, Carvajal M, García JA, Gonzalo N, et al. Nasopharyngeal Panbio COVID-19 antigen performed at point-of-care has a high sensitivity in symptomatic and asymptomatic patients with higher risk for transmission and older age. medRxiv [Preprint]; published November 17, 2020. DOI:10.1101/2020.11.16.20230003.

4. Klein JAF, Krüger LJ, Tobian F, Gaeddert M, Lainati F, Schnitzler P, et al. Head-to-head performance comparison of self-collected nasal versus professional-collected nasopharyngeal swab for a WHO-listed SARS-CoV-2 antigen-detecting rapid diagnostic test. Medical Microbiology and Immunology, 2021; 210(4):181-186. DOI:10.1007/s00430-021-00710-9.

Guidelines, review and modelling (50)

1. Peeling RW, Olliaro P. Rolling out COVID-19 antigen rapid diagnostic tests: the time is now. Lancet Infectious Diseases, 2021; 21(8):1052-1053. DOI:10.1016/s1473-3099(21)00152-3.

2. Cui Z, Chang H, Wang H, Lim B, Hsu CC, Yu Y, et al. Development of a rapid test kit for SARS-CoV-2: an example of product design. Bio-Design and Manufacturing, 2020:1-4. DOI:10.1007/s42242-020-00075-7.

3. Hayer J, Kasapic D, Zemmrich C. Real-world clinical performance of commercial SARS-CoV-2 rapid antigen tests in suspected COVID-19: A systematic meta-analysis of available data as per November 20, 2020. International Journal of Infectious Diseases, 2020; 108:592-602. DOI:10.1101/2020.12.22.20248614.

4. Ndwandwe D, Mathebula L, Kamadjeu R, Wiysonge CS. Cochrane corner: rapid point-of-care antigen and molecular-based tests for the diagnosis of COVID-19 infection. The Pan African Medical Journal, 2020; 37(Suppl 1):10. DOI:10.11604/pamj.supp.2020.37.10.25982.

5. Pavelka M, Van-Zandvoort K, Abbott S, Sherratt K, Majdan M, Jarčuška P, et al. The impact of population-wide rapid antigen testing on SARS-CoV-2 prevalence in Slovakia. medRxiv [Preprint]; published March 02, 2021. DOI:10.1101/2020.12.02.20240648.

6. Ebrahimi M, Harmooshi NN, Rahim F. Diagnostic Utility of Antigen Detection Rapid Diagnostic Tests for Covid- 19: A Systematic Review and Meta-Analysis. medRxiv [Preprint]; published April 05, 2021. DOI:10.1101/2021.04.02.21254714.

7. Everitt ML, Tillery A, David MG, Singh N, Borison A, White IM. A critical review of point-of-care diagnostic technologies to combat viral pandemics. Analytica Chimica Acta, 2021; 1146:184-199. DOI:10.1016/j.aca.2020.10.009.

8. Huergo MAC, Thanh NTK. Current advances in the detection of COVID-19 and evaluation of the humoral response. Analyst, 2021; 146:382-402. DOI:10.1039/d0an01686a.

9. Laghrib F, Saqrane S, El Bouabi Y, Farahi A, Bakasse M, Lahrich S, et al. Current progress on COVID-19 related to biosensing technologies: New opportunity for detection and monitoring of viruses. Microchemical Journal, 2021; 160:105606. DOI:10.1016/j.microc.2020.105606.

10. Maddali H, Miles CE, Kohn J, O'Carroll DM. Optical Biosensors for Virus Detection: Prospects for SARS-CoV-2/COVID-19. Chembiochem, 2021; 22(7):1176-1189. DOI:10.1002/cbic.202000744.

11. Marchán-López Á, García BA. Diagnostic performance of antigen testing for SARS-CoV-2. The Journal of Pediatrics, 2021; 233:283. DOI:10.1016/j.jpeds.2021.02.052.

12. McDermott JH, Stoddard D, Ellingford JM, Gokhale D, Reynard C, Black G, et al. Utilizing point-of-care diagnostics to minimize nosocomial infection in the 2019 novel coronavirus (SARS-CoV-2) pandemic. QJM: An International Journal of Medicine, 2020; 113(12):851-853. DOI:10.1093/qjmed/hcaa185.

13. Moreira V, Mascarenhas P, Machado V, Botelho J, Mendes JJ, Taveira N, et al. Diagnosis of SARS-Cov-2 infection using specimens other than naso- and oropharyngeal swabs: a systematic review and meta-analysis. Diagnostics, 2021; 11:363. DOI:10.1101/2021.01.19.21250094.

14. Nimmo C, Agbetile J, Bhowmik A, Capocci S, Rajakulasingam RK. Implementing rapid diagnostics for COVID-19. The Lancet Respiratory Medicine, 2021; 9(1):e7. DOI:10.1016/s2213-2600(20)30526-9.

15. Raimann FJ, Piekarski F, Adam EH, Zacharowski K, Neef V. Safety considerations for the use of Point-Of-Care diagnostics during SARS-CoV-2 pandemic. Journal of Clinical Laboratory Analysis, 2021; 35(1):e23631. DOI:10.1002/jcla.23631.

16. Razmy AM, Junaideen SM. Issues of Random Sampling with Rapid Antigen Tests for COVID-19 Diagnosis: A Special Reference to Kalmunai RDHS Division. medRxiv [Preprint]; published January 20, 2021. DOI:10.1101/2021.01.11.21249636.

17. Salcedo N, Harmon A, Herrera BB. Pooling of Samples for SARS-CoV-2 Detection Using a Rapid Antigen Test. Frontiers in Tropical Diseases, 2021; 2:16. DOI:10.3389/fitd.2021.707865.

18. Sanderlin JS, Golding JD, Wilcox T, Mason DH, McKelvey KS, Pearson DE, et al. Occupancy modeling and resampling overcomes low test sensitivity to produce accurate SARS-CoV-2 prevalence estimates. BMC Public Health, 2021; 21(1):577. DOI:10.1186/s12889-021-10609-y.

19. Scheier T, Schibli A, Eich G, Rüegg C, Kube F, Schmid A, et al. Universal Admission Screening for SARS-CoV-2 Infections among Hospitalized Patients, Switzerland, 2020. Emerging Infectious Diseases, 2021; 27(2). DOI:10.3201/eid2702.202318.

20. Stovitz SD. In suspected SARS-CoV-2, rapid antigen detection tests had 67% to 73% sensitivity and 98% to 100% specificity. Annals of Internal Medicine, 2021; 174:JC56. DOI:10.7326/acpj202105180-056.

21. van Beek J, Igloi Z, Boelsums T, Fanoy E, Gotz H, Molenkamp R, et al. From more testing to smart testing: data-guided SARS-CoV-2 testing choices. medRxiv [Preprint]; published October 14, 2020. DOI:10.1101/2020.10.13.20211524.

22. Weiss G, Bellmann-Weiler R. Rapid antigen testing and non-infectious shedding of SARS-Cov2. Infection, 2021; 49(9):789-790. DOI:10.1007/s15010-020-01570-w.

23. Ehrenberg A, Moehle E, Brook C, Doudna Cate A, Witkowsky L, Sachdeva R, et al. Launching a saliva-based SARS-CoV-2 surveillance testing program on a university campus. PLoS ONE, 2021; 16:e0251296. DOI:10.1371/journal.pone.0251296.

24. Boďová K, Kollár R. Spatial scales, patterns, and positivity trends of SARS-CoV-2 pandemics in mass rapid antigen testing in Slovakia. PLoS ONE, 2020; 16:e0256669. DOI:10.1101/2020.12.23.20248808.

25. Canadian Public Health Laboratory Network. Interim guidance on the use of the Abbott Panbio™ COVID-19 Antigen Rapid Test. Canada Communicable Disease Report, 2021; 47(1):17-22. DOI:10.14745/ccdr.v47i01a04.

26. Crozier A, Rajan S, Buchan I, McKee M. Put to the test: use of rapid testing technologies for covid-19. British Medical Journal, 2021; 372:n208. DOI:10.1136/bmj.n208.

27. D'Agostino McGowan L, Lee E, Grantz K, Kucirka L, Gurley E, Lessler J. Testing out of quarantine. medRxiv [Preprint]; published February 01, 2021. DOI:10.1101/2021.01.29.21250764.

28. Editorial. Rapid and frequent testing. Nature Biomedical Engineering, 2020; 4(12):1121-1122. DOI:10.1038/s41551-020-00670-0.

29. Fitzpatrick MC, Pandey A, Wells CR, Sah P, Galvani AP. Buyer beware: inflated claims of sensitivity for rapid COVID-19 tests. Lancet, 2021; 397(10268):24-25. DOI:10.1016/s0140-6736(20)32635-0.

30. Frnda J, Durica M. On Pilot Massive COVID-19 Testing by Antigen Tests in Europe. Case Study: Slovakia. Infectious Disease Reports, 2021; 13(1):45-57. DOI:10.3390/idr13010007.

31. Ghaffari A, Meurant R, Ardakani A. COVID-19 Point-of-Care Diagnostics That Satisfy Global Target Product Profiles. Diagnostics, 2021; 11(1):115. DOI:10.3390/diagnostics11010115.

32. Ruhan A, Wang H, Wang W, Tan W. Summary of the Detection Kits for SARS-CoV-2 Approved by the National Medical Products Administration of China and Their Application for Diagnosis of COVID-19. Virologica Sinica, 2020; 35(6):699-712. DOI:10.1007/s12250-020-00331-1.

33. Parvu V, Gary D, Mann J, Lin Y-C, Mills D, Cooper L, et al. Clinical and experimental factors that affect the reported performance characteristics of rapid testing for SARS-CoV-2. Frontiers in Microbiology, 2021; 12:2611. DOI:doi.org/10.3389/fmicb.2021.714242.

34. Azzi L. Saliva is the Key Element for Severe Acute Respiratory Syndrome Coronavirus 2 (SARS-CoV-2) Mass Screening. Clinical Infectious Diseases, 2021; 73(3):e566-e568. DOI:10.1093/cid/ciaa1440.

35. Deeks JJ, Raffle AE. Lateral flow tests cannot rule out SARS-CoV-2 infection (vol 371, m4787, 2020). British Medical Journal, 2021; 373:m4787. DOI:10.1136/bmj.n1624.

36. Diel R, Nienhaus A. Point-of-care COVID-19 antigen testing in German emergency rooms - a cost-benefit analysis. Pulmonology, 2021. DOI:10.1016/j.pulmoe.2021.06.009.

37. Estelle CD, Perl TM. To Test or Not to Test: COVID-19 Prevention Strategies to Keep Large Gatherings Safe. Annals of Internal Medicine, 2021; Online ahead of print. DOI:10.7326/m21-2976.

38. Fernández-Villa T, Vazquez-Casares A, Rivero-Rodriguez A, Carvajal-Ureña A, Martín V. Rapid antigen test for SARS-CoV-2 and primary health care. Journal of Infection, 2021; 83:237-279. DOI:10.1016/j.jinf.2021.05.001.

39. Ferretti L, Wymant C, Nurtay A, Zhao L, Hinch R, Bonsall D, et al. Modelling the effectiveness and social costs of daily lateral flow antigen tests versus quarantine in preventing onward transmission of COVID-19 from traced contacts. medRxiv [Preprint]; published August 08, 2021. DOI:10.1101/2021.08.06.21261725.

40. Hledík M, Polechová J, Beiglböck M, Herdina AN, Strassl R, Posch M. Analysis of the specificity of a COVID-19 antigen test in the Slovak mass testing program. PLoS ONE, 2021; 16(7):e0255267. DOI:10.1371/journal.pone.0255267.

41. Lekpa FK, Njonnou SRS, Balti E, Luma HN, Choukem SP. Negative antigen RDT and RT-PCR results do not rule out COVID-19 if clinical suspicion is strong. Lancet Infectious Diseases, 2021; 21(9):1209. DOI:10.1016/s1473-3099(21)00271-1.

42. Llibre JM, Videla S, Clotet B, Revollo B. Screening for SARS-CoV-2 Antigen Before a Live Indoor Music Concert: An Observational Study. Annals of Internal Medicine, 2021; Online ahead of print. DOI:10.7326/m21-2278.

43. Love J, Wimmer M, Toth DJA, Chandran A, Makhija D, Cooper C, et al. Comparison of antigen- and RT-PCR-based testing strategies for detection of Sars-Cov-2 in two high-exposure settings. PLoS ONE, 2021; 16(9):e0253407. DOI:10.1101/2021.06.03.21258248.

44. Pan D, Sze S, Abraham S, Williams CM, Tang JW, Barer MR, et al. Rapid tests for quantification of infectiousness are urgently required in patients with COVID-19. Lancet Microbe, 2021; 2(7):E286-E287. DOI:10.1016/s2666-5247(21)00089-6.

45. Peacock FW, Dzieciatkowski T, Chirico F, Szarpak L. Self-testing with antigen tests as a method for reduction SARS-CoV-2. The American Journal of Emergency Medicine, 2021; Online ahead of print. DOI:10.1016/j.ajem.2021.05.010.

46. Riccò M, Ranzieri S, Marchesi F. Rapid antigen tests for large-scale diagnostic campaigns: A case study from North-Eastern Italy. Journal of Infection, 2021; 82(5):e39-e40. DOI:10.1016/j.jinf.2021.01.011.

47. Ricks S, Kendall E, Dowdy D, Sacks J, Schumacher S, Arinaminpathy N. Quantifying the potential value of antigen-detection rapid diagnostic tests for COVID-19: a modelling analysis. BMC Medicine, 2020; 19:75. DOI:10.1101/2020.11.20.20235317.

48. Wan Z, Zhao Y, Lu R, Dong Y, Zhang C. Rapid antigen detection alone may not be sufficient for early diagnosis and/or mass screening of COVID-19. Journal of Medical Virology, 2021; Online ahead of print. DOI:10.1002/jmv.27236.

49. Willeit P, Bernar B, Zurl C, Al-Rawi M, Berghold A, Bernhard D, et al. Sensitivity and specificity of the antigen-based anterior nasal self-testing programme for detecting SARS-CoV-2 infection in schools, Austria, March 2021. Eurosurveillance, 2021; 26(34):2100797. DOI:10.2807/1560-7917.Es.2021.26.34.2100797.

50. Zettler M, Gajra A, Feinberg B. COVID-19 Rapid Antigen Test False Positives and False Negatives Reported to the FDA Manufacturer and User Facility Device Experience Database. Value in Health, 2021; 24:S218-S218. DOI:10.1016/j.jval.2021.04.1094.

Monitoring (41)

1. Abdelrazik AM, Elshafie SM, Abdelaziz HM. Potential Use of Antigen-Based Rapid Test for SARS- CoV-2 in Respiratory Specimens in LowResource Settings in Egypt for Symptomatic Patients and High-Risk Contacts. Laboratory Medicine, 2021; 52(2):E46-E49. DOI:10.1093/labmed/lmaa104.

2. Aoki K, Nagasawa T, Ishii Y, Yagi S, Okuma S, Kashiwagi K, et al. Clinical validation of quantitative SARS-CoV-2 antigen assays to estimate SARS-CoV-2 viral loads in nasopharyngeal swabs. Journal of Infection and Chemotherapy, 2020; 27(4):613-6. DOI:10.1016/j.jiac.2020.11.021.

3. Basile L, Guadalupe-Fernandez V, Guijarro MV, Mateo AM, Navas PC, Pena JM, et al. Diagnostic Performance of Ag-RDTs and NAAT for SARS-CoV2 Identification in Symptomatic Patients in Catalonia. Viruses-Basel, 2021; 13(5):908. DOI:10.3390/v13050908.

4. Basso D, Aita A, Padoan A, Cosma C, Navaglia F, Moz S, et al. Salivary SARS-CoV-2 antigen rapid detection: a prospective cohort study. Clinica Chimica Acta, 2020; 517:54-59. DOI:10.1016/j.cca.2021.02.014.

5. Bonde J, Ejegod D, Pedersen H, Smith B, Cortes D, Leding C, et al. Clinical validation of point-of-care SARS-COV-2 BD Veritor antigen test by a single throat swab for rapid COVID-19 status on hospital patients predominantly without overt COVID symptoms. medRxiv [Preprint]; published April 17, 2021. DOI:10.1101/2021.04.12.21255299.

6. Boum Y, Fai KN, Nicolay B, Mboringong AB, Bebell LM, Ndifon M, et al. Performance and operational feasibility of antigen and antibody rapid diagnostic tests for COVID-19 in symptomatic and asymptomatic patients in Cameroon: a clinical, prospective, diagnostic accuracy study. Lancet Infectious Diseases, 2021; 21:1089-1096. DOI:10.1016/s1473-3099(21)00132-8.

7. Courtellemont L, Guinard J, Guillaume C, Giaché S, Rzepecki V, Seve A, et al. High performance of a novel antigen detection test on nasopharyngeal specimens for diagnosing SARS-CoV-2 infection. Journal of Medical Virology, 2021; 93(5):3152-3157. DOI:10.1002/jmv.26896.

8. Eshghifar N, Busheri A, Shrestha R, Beqaj S. Evaluation of Analytical Performance of Seven Rapid Antigen Detection Kits for Detection of SARS-CoV-2 Virus. International Journal of General Medicine, 2021; 14:435-440. DOI:10.2147/ijgm.S297762.

9. Gitaka J, Muthamia E, Mbugua S, Mungai M, Bandawe G, Qadri F, et al. Assessment of performance and implementation characteristics of rapid point of care SARS-CoV-2 antigen testing in Kenya. medRxiv [Preprint]; published June 09, 2021. DOI:10.1101/2021.06.03.21258290.

10. Hagbom M, Carmona-Vicente N, Sharma S, Olsson H, Jämtberg M, Nilsdotter-Augustinsson Å, et al. Evaluation of SARS-CoV-2 rapid antigen diagnostic tests for saliva samples. medRxiv [Preprint]; published May 16, 2021. DOI:10.1101/2021.05.14.21257100.

11. Hingrat QL, Visseaux B, Laouenan C, Tubiana S, Bouadma L, Yazdanpanah Y, et al. Detection of SARS-CoV-2 N-antigen in blood during acute COVID-19 provides a sensitive new marker and new testing alternatives. Clinical Microbiology and Infection, 2020; 27(5):789.e1-789.e5. DOI:10.1016/j.cmi.2020.11.025.

12. Hirotsu Y, Sugiura H, Maejima M, Hayakawa M, Mochizuki H, Tsutsui T, et al. Comparison of Roche and Lumipulse quantitative SARS-CoV-2 antigen test performance using automated systems for the diagnosis of COVID-19. International Journal of Infectious Diseases, 2021; 108:263-269. DOI:10.1016/j.ijid.2021.05.067.

13. Kiro VV, Gupta A, Singh P, Sharad N, Khurana S, Prakash S, et al. Evaluation of COVID-19 Antigen Fluorescence Immunoassay Test for Rapid Detection of SARS-CoV-2. Journal of Global Infectious Diseases, 2021; 13(2):91-93. DOI:10.4103/jgid.jgid_316_20.

14. Kobayashi R, Murai R, Asanuma K, Fujiya Y, Takahashi S. Evaluating a novel, highly sensitive, and quantitative reagent for detecting SARS-CoV-2 antigen. Journal of Infection and Chemotherapy, 2021; 27(6):800-807. DOI:10.1016/j.jiac.2021.01.007.

15. Kritikos A, Caruana G, Brouillet R, Miroz J-P, Samia A-M, Geraldine S, et al. Sensitivity of rapid antigen testing and RT-PCR performed on nasopharyngeal swabs versus saliva samples in COVID-19 hospitalized patients: results of a prospective comparative trial (RESTART). Microorganisms, 2021; 9(9):1910. DOI:10.1101/2021.04.09.21255105.

16. Kwon J, Ko E, Cho S-Y, Lee Y-H, Jun S, Lee K, et al. Bean extract-based gargle for efficient diagnosing COVID-19 at early-stage using rapid antigen tests : a clinical, prospective, diagnostic study. medRxiv [Preprint]; published August 19, 2021. DOI:10.1101/2021.08.13.21261463.

17. Lanser L, Bellmann-Weiler R, Öttl KW, Huber L, Griesmacher A, Theurl I, et al. Evaluating the clinical utility and sensitivity of SARS-CoV-2 antigen testing in relation to RT-PCR Ct values. Infection, 2020; 49(3):555-557. DOI:10.1007/s15010-020-01542-0.

18. Lv Y, Ma Y, Si Y, Zhu X, Zhang L, Feng H, et al. Rapid SARS-CoV-2 antigen detection potentiates early diagnosis of COVID-19 disease. Bioscience Trends, 2021; 15(2):93-99. DOI:10.5582/bst.2021.01090.

19. Menchinelli G, Bordi L, Marzialiotti F, Palucci I, Capobianchi M, Sberna G, et al. Lumipulse G SARS-CoV-2 Ag Assay Evaluation for SARS-CoV-2 Antigen Detection Using 594 Nasopharyngeal Swab Samples from Different Testing Groups. Clinical Chemistry and Laboratory Medicine, 2021; 59(8):1468-1476. DOI:10.1515/cclm-2021-0182.

20. Miyakawa K, Funabashi R, Yamaoka Y, Jeremiah SS, Katada J, Wada A, et al. SARS-CoV-2 antigen rapid diagnostic test enhanced with silver amplification technology. medRxiv [Preprint]; published January 31, 2021. DOI:10.1101/2021.01.27.21250659.

21. Nagura-Ikeda M, Imai K, Tabata S, Miyoshi K, Murahara N, Mizuno T, et al. Clinical Evaluation of Self-Collected Saliva by Quantitative Reverse Transcription-PCR (RT-qPCR), Direct RT-qPCR, Reverse Transcription-Loop-Mediated Isothermal Amplification, and a Rapid Antigen Test To Diagnose COVID-19. Journal of Clinical Microbiology, 2020; 58(9). DOI:10.1128/jcm.01438-20.

22. Oh SM, Jeong H, Chang E, Choe PG, Kang CK, Park WB, et al. Clinical Application of the Standard Q COVID-19 Ag Test for the Detection of SARS-CoV-2 Infection. Journal of Korean Medical Science, 2021; 36(14):e101. DOI:10.3346/jkms.2021.36.e101.

23. Olearo F, Nörz D, Heinrich F, Sutter JP, Rödel K, Schultze A, et al. Handling and accuracy of four rapid antigen tests for the diagnosis of SARS-CoV-2 compared to RT-qPCR. Journal of Clinical Virology, 2020; 137:104782. DOI:10.1016/j.jcv.2021.104782.

24. Peña-Rodrígez M, Viera-Segura O, García-Chagollán M, Zepeda-Nuño JS, Muñoz-Valle JF, Mora-Mora J, et al. Performance evaluation of a lateral flow assays for nasopharyngeal antigen detection for SARS-CoV-2 diagnosis. Journal of Clinical Laboratory Analysis, 2021; 35:e23745. DOI:10.1002/jcla.23745.

25. Regev-Yochay G, Kriger O, Beni S, Rubin C, Mina M, Mechnik B, et al. Real World Performance of SARS-CoV-2 Antigen Rapid Diagnostic Tests in Various Clinical Settings. medRxiv [Preprint]; published March 05, 2021. DOI:10.1101/2021.03.02.21252400.

26. Ren A, Sohaei D, Zacharioudakis I, Sigal G, Stengelin M, Matthew A, et al. Ultrasensitive assay for saliva-based SARS-CoV-2 antigen detection. medRxiv [Preprint]; published February 19, 2021. DOI:10.1101/2021.02.17.21251863.

27. Rodrigues J, Gouveia C, Santos MA, Costa O, Côrte-Real R, Brito MJ. Comparison of nasopharyngeal samples for SARS-CoV-2 detection in a paediatric cohort. Journal of Paediatrics and Child Health, 2021; 57(7):1078-1081. DOI:10.1111/jpc.15405.

28. Saeed U, Uppal SR, Piracha ZZ, Rasheed A, Aftab Z, Zaheer H, et al. Evaluation of SARS-CoV-2 antigen-based rapid diagnostic kits in Pakistan: formulation of COVID-19 national testing strategy. Virology Journal, 2021; 18(1):34. DOI:10.1186/s12985-021-01505-3.

29. Salvagno GL, Nocini R, Gianfilippi G, Fiorio G, Pighi L, De Nitto S, et al. Performance of Fujirebio Espline SARS-CoV-2 rapid antigen test for identifying potentially infectious individuals. Diagnosis, 2021; Online ahead of print. DOI:10.1515/dx-2021-0107.

30. Smith R, Gibson L, Martinez P, Ke R, Mirza A, Conte M, et al. Longitudinal assessment of diagnostic test performance over the course of acute SARS-CoV-2 infection. The Journal of Infectious Diseases, 2021; 224(6):976–982. DOI:10.1093/infdis/jiab337.

31. Stokes W, Berenger BM, Singh T, Adeghe I, Schneider A, Portnoy D, et al. Acceptable performance of the Abbott ID NOW among symptomatic individuals with confirmed COVID-19. Journal of Medical Microbiology, 2021; 70(7):001372. DOI:10.1099/jmm.0.001372.

32. Thommes L, Burkert FR, Öttl KW, Goldin D, Loacker L, Lanser L, et al. Comparative evaluation of four SARS-CoV-2 antigen tests in hospitalized patients. International Journal of Infectious Diseases, 2021; 105:144–146. DOI:10.1016/j.ijid.2021.02.052.

33. Veyrenche N, Bollore K, Pisoni A, Bedin AS, Mondain AM, Ducos J, et al. Diagnosis value of SARS-CoV-2 antigen/antibody combined testing using rapid diagnostic tests at hospital admission. Journal of Medical Virology, 2020; 39(5):3069-3076. DOI:10.1002/jmv.26855.

34. Yamamoto K, Nagashima M, Yoshida I, Sadamasu K, Kurokawa M, Nagashima M, et al. Does the SARS-CoV-2 rapid antigen test result correlate with the viral culture result? Journal of Infection and Chemotherapy, 2021; 27(8):1273-1275. DOI:10.1016/j.jiac.2021.05.006.

35. Yokota I, Sakurazawa T, Sugita J, Iwasaki S, Yasuda K, Yamashita N, et al. Performance of Qualitative and Quantitative Antigen Tests for SARS-CoV-2 Using Saliva. Infectious Disease Reports, 2021; 13(3):742-747. DOI:10.3390/idr13030069.

36. Yokota I, Shane PY, Okada K, Unoki Y, Yang Y, Iwasaki S, et al. A novel strategy for SARS-CoV-2 mass screening with quantitative antigen testing of saliva: a diagnostic accuracy study. Lancet Microbe, 2021; 2(8):E397-E404. DOI:10.1016/s2666-5247(21)00092-6.

37. Zacharias M, Stangl V, Thüringer A, Loibner M, Wurm P, Wolfgruber S, et al. Rapid Antigen Test for Postmortem Evaluation of SARS-CoV-2 Carriage. Emerging Infectious Diseases, 2021; 27(6). DOI:10.3201/eid2706.210226.

38. Mak GCK, Lau SSY, Wong KKY, Chow NLS, Lau CS, Ng KHL, et al. Evaluation of automated antigen detection test for detection of SARS-CoV-2. Diagnostic Microbiology and Infectious Disease, 2021; 101(4):115490. DOI:10.1016/j.diagmicrobio.2021.115490.

39. Reza S, Corentin D, Te-Din H, Pierre B, Stéphanie E, Isaline W, et al. Rapid COVID-19 antigenic tests: Usefulness of a modified method for diagnosis. Journal of Medical Virology, 2021; 93(9):5655-5659. DOI:10.1002/jmv.27094.

40. Mueller T, Kompatscher J, La Guardia M. Diagnostic performance of the Elecsys SARS-CoV-2 antigen assay in the clinical routine of a tertiary care hospital: Preliminary results from a single-center evaluation. Journal of Clinical Laboratory Analysis, 2021; 35(8):e23906. DOI:10.1002/jcla.23906.

41. Nomoto H, Yamamoto K, Yamada G, Suzuki M, Kinoshita N, Takasaki J, et al. Time-course evaluation of the quantitative antigen test for severe acute respiratory syndrome coronavirus 2: The potential contribution to alleviating isolation of COVID-19 patients. Journal of Infection and Chemotherapy, 2021; 27(11):1669-1673. DOI:10.1016/j.jiac.2021.08.015.

No estimates for sensitivity and specificity (31)

1. Savini S, Monaco D, Turci C, Ursino S, Matera C, Marchini R, et al. Prevention of the spread of SARS COV-2 by Rapid Antigenic Tests on the passengers entering an Italian seaport. Annali di Igiene, 2021; 33(5):518-520. DOI:10.7416/ai.2021.2450.

2. Wong S, Romney M, Matic N, Haase K, Ranger M, Dhari R, et al. Feasibility and utility of rapid antigen testing for COVID-19 in a university residence: a cross sectional study. medRxiv [Preprint]; published May 26, 2021. DOI:10.1101/2021.05.24.21257732.

3. Lamb G, Heskin J, Randell P, Mughal N, Moore LS, Jones R, et al. Real-world evaluation of COVID-19 lateral flow device (LFD) mass-testing in healthcare workers at a London hospital; a prospective cohort analysis. Journal of Infection, 2021; 83(4):452-457. DOI:10.1016/j.jinf.2021.07.038.

4. Kumar A, Kunjukutty R, Thaha A, Srikumar S, Madhusoodanan H, David S, et al. Universal screening for SARS-CoV-2 in pregnant women using a combination of antigen and RT-PCR testing. Le Infezioni in Medicina, 2021; 29(2):294-296.

5. Bello-Chavolla OY, Antonio-Villa NE, Fernández-Chirino L, Guerra E, Fermín-Martínez C, Márquez-Salinas A, et al. Diagnostic performance and clinical implications of rapid SARS-CoV-2 antigen testing in Mexico using real-world nationwide COVID-19 registry data. PLoS ONE, 2021; 16:e0256447. DOI:10.1101/2021.01.02.21249141.

6. Dalal A, Sonika U, Kumar M, George R, Kumar A, Srivastava S, et al. COVID-19 Rapid Antigen Test: Role in Screening Prior to Gastrointestinal Endoscopy. Clinical Endoscopy, 2021; 54:522-525. DOI:10.5946/ce.2020.295.

7. Downs LO, Eyre DW, O'Donnell D, Jeffery K. Home-based SARS-CoV-2 lateral flow antigen testing in hospital workers. Journal of Infection, 2021; 82(2):282-327. DOI:10.1016/j.jinf.2021.01.008.

8. Hoehl S, Schenk B, Rudych O, Gottig S, Foppa I, Kohmer N, et al. High-Frequency Self-Testing by Schoolteachers for Sars-Cov-2 Using a Rapid Antigen Test Results of the Safe School Hesse study. Deutsches Arzteblatt International, 2021; 118(14):252-253. DOI:10.3238/arztebl.m2021.0187.

9. Marco A, Solé C, Abdo IJ, Turu E. Low sensitivity of rapid antigenic tests as a screening method in an outbreak of SARS-CoV-2 infection in prison. Enfermedades Infecciosas y Microbiología Clínica, 2021; online ahead of print. DOI:10.1016/j.eimc.2021.01.016.

10. Matsuda EM, de Campos IB, de Oliveira IP, Colpas DR, Carmo A, Brígido LFM. Field evaluation of COVID-19 antigen tests versus RNA based detection: Potential lower sensitivity compensated by immediate results, technical simplicity and low cost. Journal of Medical Virology, 2021; 93(7):4405-4410. DOI:10.1002/jmv.26985.

11. Moreno G, Braun K, Pray I, Segaloff H, Lim A, Poulson K, et al. SARS-CoV-2 transmission in intercollegiate athletics not fully mitigated with daily antigen testing. medRxiv [Preprint]; published March 06, 2021. DOI:10.1101/2021.03.03.21252838.

12. Yamayoshi S, Sakai-Tagawa Y, Koga M, Akasaka O, Nakachi I, Koh H, et al. Comparison of Rapid Antigen Tests for COVID-19. Viruses-Basel, 2020; 12(12):1420. DOI:10.3390/v12121420.

13. Yokota I, Shane P, Teshima T. Logistic advantage of two-step screening strategy for SARS-CoV-2 at airport quarantine. Travel Medicine and Infectious Disease, 2021; 43:102127. DOI:10.1101/2021.01.25.21250509.

14. Häuser F, Sprinzl MF, Dreis KJ, Renzaho A, Youhanen S, Kremer WM, et al. Evaluation of a laboratory-based high-throughput SARS-CoV-2 antigen assay for non-COVID-19 patient screening at hospital admission. Medical Microbiology and Immunology, 2021; 201(2-3):165-171. DOI:10.1007/s00430-021-00706-5.

15. Colavita F, Vairo F, Meschi S, Valli MB, Lalle E, Castilletti C, et al. COVID-19 Rapid Antigen Test as Screening Strategy at Points of Entry: Experience in Lazio Region, Central Italy, August-October 2020. Biomolecules, 2021; 11(3). DOI:10.3390/biom11030425.

16. Munne K, Bhanothu V, Mayekar A, Birje S, Bhor V, Patel V, et al. A retrospective analysis of COVID-19 diagnosis results obtained by rapid antigen tests and RT-PCR: Implications for disease management. Indian Journal of Medical Microbiology, 2021; Online ahead of print. DOI:10.1016/j.ijmmb.2021.05.006.

17. Murillo-Zamora E, Trujillo X, Huerta M, Ríos-Silva M, Mendoza-Cano O. Performance of Antigen-Based Testing as Frontline Diagnosis of Symptomatic COVID-19. Medicina (Kaunas), 2021; 57(8):852. DOI:10.3390/medicina57080852.

18. Langalia AK, Patel D, Prajapati D, Munia V, Shah D, Lalwani Y, et al. Large-Scale Rapid Antigen Testing of Migrants for Detection of SARS-CoV-2 Virus by Dental Interns at Various Checkpoints of Ahmedabad City, India: A Short Communication Report. Asia-Pacific Journal of Public Health, 2021; Online ahead of print. DOI:10.1177/10105395211012914.

19. Wachinger J, Olaru ID, Horner S, Schnitzler P, Heeg K, Denkinger C. The potential of SARS-CoV-2 antigen-detection tests in the screening of asymptomatic persons. Clinical Microbiology and Infection, 2021; Online ahead of print. DOI:10.1101/2021.06.07.21258465.

20. Barry G, McCarney C, Farrelly M, Breathnach R, Mooney C, More S. Rapid antigen testing for SARS-CoV-2 infection in a university setting in Ireland: learning from a 6-week pilot study. medRxiv [Preprint]; published August 07, 2021. DOI:10.1101/2021.08.05.21261660.

21. Fernández-Vázquez JP, Reguero S, Sánchez-Antolín G, Martín-Sánchez V. Population-based screening for acute SARS-CoV-2 infection using rapid antigen testing and the 5% pre-test probability. Is the specifity our problem? Enfermedades Infecciosas y Microbiología Clínica, 2021; Online ahead of print. DOI:10.1016/j.eimc.2021.04.007.

22. Hada V, Rath RS, Mohanty A, Sahai R, Kumar K, Kumar S, et al. Comparison of Positivity Rates of Rapid Antigen Testing and Real-Time Polymerase Chain Reaction for COVID-19 During the First and Second Waves of the Pandemic in Eastern Uttar Pradesh, India. Cureus, 2021; 13(7):e16206. DOI:10.7759/cureus.16206.

23. Kanji J, Proctor D, Stokes W, Berenger B, Silvius J, Tipples G, et al. Multi-centre post-implementation evaluation of SARS-CoV-2 antigen-based point of care tests used for asymptomatic screening of continuing care healthcare workers. Journal of Clinical Microbiology, 2021; Online ahead of print. DOI:10.1128/JCM.01411-21.

24. Kobayashi R, Murai R, Moriai M, Nirasawa S, Yonezawa H, Kondoh T, et al. Evaluation of false positives in the SARS-CoV-2 quantitative antigen test. Journal of Infection and Chemotherapy, 2021; 27(10):1477-1481. DOI:10.1016/j.jiac.2021.06.019

10.1016/j.jiac.2021.06.019. Epub 2021 Jun 25.

25. Kepka S, Ohana M, Séverac F, Muller J, Bayle E, Ruch Y, et al. Rapid Antigen Test Combined with Chest Computed Tomography to Rule Out COVID-19 in Patients Admitted to the Emergency Department. Journal of Clinical Medicine, 2021; 10(16). DOI:10.3390/jcm10163455.

26. Koller G, Morrell AP, Galao RP, Pickering S, MacMahon E, Johnson J, et al. More than the Eye Can See: Shedding New Light on SARS-CoV-2 Lateral Flow Device-Based Immunoassays. ACS Applied Materials & Interfaces, 2021; 13(22):25694-25700. DOI:10.1021/acsami.1c04283.

27. Kretschmer A, Kossow A, Grüne B, Schildgen O, Mathes T, Schildgen V. False positive rapid antigen tests for SARS-CoV-2 in the real-world and their economic burden. Journal of Infection, 2021; Online ahead of print. DOI:10.1016/j.jinf.2021.08.020.

28. Kotsiou OS, Pantazopoulos I, Papagiannis D, Fradelos EC, Kanellopoulos N, Siachpazidou D, et al. Repeated Antigen-Based Rapid Diagnostic Testing for Estimating the Coronavirus Disease 2019 Prevalence from the Perspective of the Workers' Vulnerability before and during the Lockdown. International Journal of Environmental Research and Public Health, 2021; 18(4). DOI:10.3390/ijerph18041638.

29. Leber W, Lammel O, Siebenhofer A, Redlberger-Fritz M, Panovska-Griffiths J, Czypionka T. Comparing the diagnostic accuracy of point-of-care lateral flow antigen testing for SARS-CoV-2 with RT-PCR in primary care (REAP-2). EClinicalMedicine, 2021:101011. DOI:10.1016/j.eclinm.2021.101011.

30. Mboma O, Rieke E, Ahmad-Nejad P, Wirth S, Aydin M. Diagnostic Performance of SARS-CoV-2 Rapid Antigen Test in a Large, German Cohort. Children (Basel), 2021; 8(8):682. DOI:10.3390/children8080682.

31. Blairon L, Wilmet A, Beukinga I, Tre-Hardy M. Implementation of rapid SARS-CoV-2 antigenic testing in a laboratory without access to molecular methods: Experiences of a general hospital. Journal of Clinical Virology, 2020; 129:104472. DOI:10.1016/j.jcv.2020.104472.

No point of care (14)

1. Gili A, Paggi R, Russo C, Cenci E, Pietrella D, Graziani A, et al. Evaluation of automated test Lumipulse G SARS-CoV-2 antigen assay for detection of SARS-CoV-2 nucleocapsid protein (NP) in nasopharyngeal swabs for community and population screening. International Journal of Infectious Diseases, 2021; 105:391-396. DOI:10.1016/j.ijid.2021.02.098.

2. Hirotsu Y, Maejima M, Shibusawa M, Amemiya K, Nagakubo Y, Hosaka K, et al. Prospective Study of 1,308 Nasopharyngeal Swabs from 1,033 Patients using the LUMIPULSE SARS-CoV-2 Antigen Test: Comparison with RT-qPCR. International Journal of Infectious Diseases, 2021; 105:7-14. DOI:10.1016/j.ijid.2021.02.005.

3. Koskinen J, Antikainen P, Hotakainen K, Haveri A, Ikonen N, Savolainen-Kopra C, et al. Clinical Validation of Automated and Rapid mariPOC SARS-CoV-2 Antigen Test. Scientific Reports, 2021; 11:20363. DOI:10.1101/2021.02.08.21250086.

4. Mayanskiy N, Brzhozovskaya E, Fedorova N, Lebedin Y. Parallel detection of SARS-CoV-2 RNA and nucleocapsid antigen in nasopharyngeal specimens from a COVID-19 patient screening cohort. International Journal of Infectious Diseases, 2021; 108:330-332. DOI:10.1016/j.ijid.2021.05.082.

5. Asai N, Sakanashi D, Ohashi W, Nakamura A, Kawamoto Y, Miyazaki N, et al. Efficacy and validity of automated quantitative chemiluminescent enzyme immunoassay for SARS-CoV-2 antigen test from saliva specimen in the diagnosis of COVID-19. Journal of Infection and Chemotherapy, 2021; 27(7):1039-1042. DOI:10.1016/j.jiac.2021.03.021.

6. Audigé A, Böni J, Schreiber PW, Scheier T, Buonomano R, Rudiger A, et al. Reduced Relative Sensitivity of the Elecsys SARS-CoV-2 Antigen Assay in Saliva Compared to Nasopharyngeal Swabs. Microorganisms, 2021; 9(8):1700. DOI:10.3390/microorganisms9081700.

7. Hartard C, Berger S, Josse T, Schvoerer E, Jeulin H. Performance evaluation of an automated SARS-CoV-2 Ag test for the diagnosis of COVID-19 infection on nasopharyngeal swabs. Clinical Chemistry and Laboratory Medicine, 2021. DOI:10.1515/cclm-2021-0569.

8. Iqbal B, Khan M, Shah N, Dawood MM, Jehanzeb V, Shafi M. Comparison of SARS-CoV-2 antigen electrochemiluminescence immunoassay to RT-PCR assay for laboratory diagnosis of COVID-19 in Peshawar. Diagnosis (Berl), 2021. DOI:10.1515/dx-2021-0078.

9. Lefever S, Indevuyst C, Cuypers L, Dewaele K, Yin N, Cotton F, et al. Comparison of the quantitative DiaSorin Liaison antigen test to RT-PCR for the diagnosis of COVID-19 in symptomatic and asymptomatic outpatients. Journal of Clinical Microbiology, 2021; 59(7):e0037421. DOI:10.1128/JCM.00374-21.

10. Levett PN, Cheung B, Kustra J, Pidduck T, Mak A, Tsang F, et al. Evaluation of a high volume antigen test for detection of SARS-CoV-2. Journal of Clinical Virology, 2021; 142:104938. DOI:10.1016/j.jcv.2021.104938.

11. Matsuzaki N, Orihara Y, Kodana M, Kitagawa Y, Matsuoka M, Kawamura R, et al. Evaluation of a chemiluminescent enzyme immunoassay-based high-throughput SARS-CoV-2 antigen assay for the diagnosis of COVID-19: The VITROS (R) SARS-CoV-2 Antigen Test. Journal of Medical Virology, 2021. DOI:10.1002/jmv.27153.

12. Nörz D, Olearo F, Perisic S, Bauer M, Riester E, Schneider T, et al. Multicenter evaluation of a fully automated high-throughput SARS-CoV-2 antigen immunoassay. Infectious Diseases and Therapy, 2021:1-9. DOI:10.1101/2021.04.09.21255047.

13. Salvagno GL, Gianfilippi G, Fiorio G, Pighi L, De Nitto S, Henry BM, et al. Clinical Assessment of the DiaSorin LIAISON SARS-CoV-2 Ag Chemiluminescence Immunoassay. Electronic Journal of the International Federation of Clinical Chemistry and Laboratory Medicine, 2021; 32(2):216-223.

14. Van der Moeren N, Zwart VF, Goderski G, Rijkers GT, van den Bijllaardt W, Veenemans J, et al. Performance of the Diasorin SARS-CoV-2 antigen detection assay on the LIAISON XL. Journal of Clinical Virology, 2021; 141:104909. DOI:10.1016/j.jcv.2021.104909.

Study population smaller 10 (3)

1. Gandolfo Cd, Morecchiato Fd, Pistello Mp, Rossolini GMp, Cusi MGp. Detection of SARS-CoV-2 N protein allelic variants by rapid high-throughput CLEIA antigen assay. Journal of Clinical Virology, 2021; 142:104942. DOI:10.1016/j.jcv.2021.104942.

2. Itoh K, Kawamitsu T, Osaka Y, Sato K, Suzuki Y, Kiriba C, et al. False positive results in severe acute respiratory coronavirus 2 (SARS-CoV-2) rapid antigen tests for inpatients. Journal of Infection and Chemotherapy, 2021; 27(7):1089-1091. DOI:10.1016/j.jiac.2021.03.011.

3. Kashiwagi K, Ishii Y, Aoki K, Yagi S, Maeda T, Miyazaki T, et al. Immunochromatographic test for the detection of SARS-CoV-2 in saliva. Journal of Infection and Chemotherapy, 2020; 27(2):384-386. DOI:10.1101/2020.05.20.20107631.

Use case not diagnosis (31)

1. Ahava M, Kurkela S, Kuivanen S, Lappalainen M, Jarva H, Jaaskelainen A. Detection of SARS-CoV-2 nucleocapsid antigen from serum can aid in timing of COVID-19 infection. medRxiv [Preprint]; published January 13, 2021. DOI:10.1101/2021.01.08.20248771.

2. Amer RM, Samir M, Gaber OA, El-Deeb NA, Abdelmoaty AA, Ahmed AA, et al. Diagnostic performance of rapid antigen test for COVID-19 and the effect of viral load, sampling time, subject's clinical and laboratory parameters on test accuracy. Journal of Infection and Public Health, 2021; 14(10):1446-1453. DOI:10.1016/j.jiph.2021.06.002.

3. Aoki K, Nagasawa T, Ishii Y, Yagi S, Kashiwagi K, Miyazaki T, et al. Evaluation of clinical utility of novel coronavirus antigen detection reagent, Espline SARS-CoV-2. Journal of Infection and Chemotherapy, 2021; 27(2):319-322. DOI:10.1016/j.jiac.2020.11.015.

4. Bruins M, dos Santos CO, Spoelman-Lunsche M, van den Bos-Kromhout M, Debast S. Evaluation of the Panbio rapid antigen test for COVID-19 diagnosis in symptomatic health care workers. medRxiv [Preprint]; published June 25, 2021. DOI:10.1101/2021.06.21.21259234.

5. Caputo V, Bax C, Colantoni L, Peconi C, Termine A, Fabrizio C, et al. Comparative analysis of antigen and molecular tests for the detection of Sars-CoV-2 and related variants: a study on 4266 samples. International Journal of Infectious Diseases, 2021; 108:187-189. DOI:10.1016/j.ijid.2021.04.048.

6. Demuth S, Damaschek S, Schildgen O, Schildgen V. Low sensitivity of SARS-CoV-2 rapid antigen self-tests under laboratory conditions. New Microbes and New Infections, 2021; 43:100916. DOI:10.1016/j.nmni.2021.100916.

7. Deng Q, Ye G, Pan Y, Xie W, Yang G, Li Z, et al. High Performance of SARS-Cov-2N Protein Antigen Chemiluminescence Immunoassay as Frontline Testing for Acute Phase COVID-19 Diagnosis: A Retrospective Cohort Study. Frontiers in Medicine, 2021; 8:676560. DOI:10.3389/fmed.2021.676560.

8. Ford L, Lee C, Pray IW, Cole D, Bigouette JP, Abedi GR, et al. Epidemiologic characteristics associated with SARS-CoV-2 antigen-based test results, rRT-PCR cycle threshold values, subgenomic RNA, and viral culture results from university testing. Clinical Infectious Diseases, 2021; 73:e1348-e1355. DOI:10.1093/cid/ciab303.

9. Ford L, Whaley M, Shah M, Salvatore P, Segaloff H, Delaney A, et al. Characteristics of children and antigen test performance at a SARS-CoV-2 community testing site. medRxiv [Preprint]; published July 07, 2021. DOI:10.1101/2021.07.06.21259792.

10. Frezza D, Fabbris C, Franz L, Vian E, Rigoli R, De Siati R, et al. A pilot feasibility study on SARS-CoV-2 detection method based on nasopharyngeal lavage fluid. Laryngoscope Investigative Otolaryngology, 2021; 6(4):646-649. DOI:10.1101/2021.06.14.21258619.

11. Harmon A, Chang C, Salcedo N, Sena B, Herrera BB, Bosch I, et al. Validation of an At-Home Direct Antigen Rapid Test for COVID-19. Jama Network Open, 2021; 4(8):e2126931. DOI:10.1001/jamanetworkopen.2021.26931.

12. Harmon K, de St Maurice AM, Brady AC, Swaminathan S, Aukerman DF, Rueda MA, et al. Surveillance testing for SARS-COV-2 infection in an asymptomatic athlete population: a prospective cohort study with 123 362 tests and 23 463 paired RT-PCR/antigen samples. BMJ Open Sport & Exercise Medicine, 2021; 7(2):e001137. DOI:10.1136/bmjsem-2021-001137.

13. Hirotsu Y, Maejima M, Shibusawa M, Nagakubo Y, Hosaka K, Amemiya K, et al. Comparison of automated SARS-CoV-2 antigen test for COVID-19 infection with quantitative RT-PCR using 313 nasopharyngeal swabs, including from seven serially followed patients. International Journal of Infectious Diseases, 2020; 99:397-402. DOI:10.1016/j.ijid.2020.08.029.

14. Ishii T, Sasaki M, Yamada K, Kato D, Osuka H, Aoki K, et al. Immunochromatography and chemiluminescent enzyme immunoassay for COVID-19 diagnosis. Journal of Infection and Chemotherapy, 2021; 27(6):915-918. DOI:10.1016/j.jiac.2021.02.025.

15. Jakobsen KK, Jensen JS, Todsen T, Tolsaard MG, Kirkby N, Lippert F, et al. Accuracy and cost description of rapid antigen test compared with reverse transcriptase-polymerase chain reaction for SARS-CoV-2 detection. Danish Medical Journal, 2021; 68(7):A03210217.

16. Kiyasu Y, Takeuchi Y, Akashi Y, Kato D, Kuwahara M, Muramatsu S, et al. Prospective analytical performance evaluation of the QuickNavi™-COVID19 Ag for asymptomatic individuals. Journal of Infection and Chemotherapy, 2021; 27(10):1489-1492. DOI:10.1016/j.jiac.2021.07.005.

17. Kronberg Jakobsen K, Schmidt Jensen J, Todsen T, Lippert F, Jean-Marie Martel C, Klokker M, et al. Detection of SARS-CoV-2 infection by rapid antigen test in comparison with RT-PCR in a public setting. medRxiv [Preprint]; published January 25, 2021. DOI:10.1101/2021.01.22.21250042.

18. Kweon OJ, Lim YK, Kim HR, Choi Y, Kim MC, Choi SH, et al. Evaluation of rapid SARS-CoV-2 antigen tests, AFIAS COVID-19 Ag and ichroma COVID-19 Ag, with serial nasopharyngeal specimens from COVID-19 patients. PLoS ONE, 2021; 16(4):e0249972. DOI:10.1371/journal.pone.0249972.

19. Landaas ET, Storm ML, Tollånes MC, Barlinn R, Kran AB, Bragstad K, et al. Diagnostic performance of a SARS-CoV-2 rapid antigen test in a large, Norwegian cohort. Journal of Clinical Virology, 2021; 137:104789. DOI:10.1016/j.jcv.2021.104789.

20. Le Hingrat Q, Visseaux B, Laouenan C, Tubiana S, Bouadma L, Yazdanpanah Y, et al. Detection of SARS-CoV-2 N-antigen in blood during acute COVID-19 provides a sensitive new marker and new testing alternatives. Clinical Microbiology and Infection, 2021; 27(5). DOI:10.1016/j.cmi.2020.11.025.

21. McAulay K, Kaleta EJ, Grys TE. Rapid Detection of SARS-CoV-2 Antigen from Serum in a Hospitalized Population. medRxiv [Preprint]; published December 22, 2020. DOI:10.1101/2020.12.21.20248140.

22. McKay SL, Tobolowsky FA, Moritz ED, Hatfield KM, Bhatnagar A, LaVoie SP, et al. Performance Evaluation of Serial SARS-CoV-2 Rapid Antigen Testing During a Nursing Home Outbreak. Annals of Internal Medicine, 2021; 174(7):945-951. DOI:10.7326/m21-0422.

23. Pray IW, Ford L, Cole D, Lee C, Bigouette JP, Abedi GR, et al. Performance of an Antigen-Based Test for Asymptomatic and Symptomatic SARS-CoV-2 Testing at Two University Campuses - Wisconsin, September-October 2020. Morbidity and Mortality Weekly Report, 2021; 69(5152):1642-1647. DOI:10.15585/mmwr.mm695152a3.

24. Prince-Guerra JL, Almendares O, Nolen LD, Gunn JKL, Dale AP, Buono SA, et al. Evaluation of Abbott BinaxNOW Rapid Antigen Test for SARS-CoV-2 Infection at Two Community-Based Testing Sites - Pima County, Arizona, November 3-17, 2020. Morbidity and Mortality Weekly Report, 2021; 70(3):100-105. DOI:10.15585/mmwr.mm7003e3.

25. Puyskens A, Krause E, Michel J, Nübling M, Scheiblauer H, Bourquain D, et al. Establishment of an evaluation panel for the decentralized technical evaluation of the sensitivity of 31 rapid detection tests for SARS-CoV-2 diagnostics. medRxiv [Preprint]; published May 13, 2021. DOI:10.1101/2021.05.11.21257021.

26. Rastawicki W, Gierczyński R, Juszczyk G, Mitura K, Henry BM. Evaluation of PCL rapid point of care antigen test for detection of SARS-CoV-2 in nasopharyngeal swabs. Journal of Medical Virology, 2021; 93(4):1920-1922. DOI:10.1002/jmv.26765.

27. Suliman S, Matias W, Fulcher I, Molano F, Collins S, Uceta E, et al. Evaluation of the Access Bio CareStartTM rapid SARS-CoV-2 antigen test in asymptomatic individuals tested at a community mass-testing program in Western Massachusetts. medRxiv [Preprint]; published June 20, 2021. DOI:10.1101/2021.06.17.21259109.

28. Uwamino Y, Nagata M, Aoki W, Nakagawa T, Inose R, Yokota H, et al. Accuracy of rapid antigen detection test for nasopharyngeal swab specimens and saliva samples in comparison with RT-PCR and viral culture for SARS-CoV-2 detection. Journal of Infection and Chemotherapy, 2021; 27(7):1058-1062. DOI:10.1016/j.jiac.2021.04.010.

29. Wagenhäuser I, Knies K, Rauschenberger V, Eisenmann M, McDonogh M, Petri N, et al. Clinical performance evaluation of SARS-CoV-2 rapid antigen testing in point of care usage in comparison to RT-qPCR. EBioMedicine, 2021; 69:103455. DOI:10.1016/j.ebiom.2021.103455.

30. Winkel BMF, Schram E, Gremmels H, Debast S, Schuurman R, Wensing AMJ, et al. Screening for SARS-CoV-2 infection in asymptomatic individuals using the Panbio™ COVID-19 Antigen Rapid Test (Abbott) compared to RT-qPCR. medRxiv [Preprint]; published December 04, 2020. DOI:10.1101/2020.12.03.20243311.

31. Yamamoto K, Suzuki M, Yamada G, Sudo T, Nomoto H, Kinoshita N, et al. Utility of the antigen test for coronavirus disease 2019: Factors influencing the prediction of the possibility of disease transmission. International Journal of Infectious Diseases, 2021; 104:65-72. DOI:10.1016/j.ijid.2020.12.079.

Excluded from FIND website – duplication of data (3)

1. Foundation for Innovative New Diagnostics. FIND Evaluation of Abbott Panbio COVID-19 Ag Rapid Test Device. External Report Version 21, 10 December, 2020.

2. Foundation for Innovative New Diagnostics. FIND Evaluation of Coris BioConcept COVID-19 Ag Respi-Strip. External Report Version 12, 10 December, 2020.

3. Foundation for Innovative New Diagnostics. FIND Evaluation of Shenzhen Bioeasy Biotechnology Co. Ltd. 2019-nCoV Ag Rapid Test Kit (Fluorescence). External Report Version 10, 11 February 2021, 2021.

Excluded from FIND website – monitoring (1)

1. Foundation for Innovative New Diagnostics. FIND Evaluation of SD Biosensor, Inc.; STANDARD™ F COVID-19 Ag FIA. External Report Site Specific Report Version 10, 27 April 2021, 2021.
