# Supplementary material for: Accuracy of rapid point-of-care antigen-based diagnostics for SARS-CoV-2: An updated systematic review and meta-analysis with meta-regression analyzing influencing factors
Source: PLoS Med. 2022 May 26;19(5):e1004011. doi: 10.1371/journal.pmed.1004011 (PMC9187092; doi:10.1371/journal.pmed.1004011)
Supplement: S6 Text — (DOCX) [file pmed.1004011.s024.docx]

**S6 – Studies potentially influenced by the test manufacturer (number of studies: 47)**

1. Albert E, Torres I, Bueno F, Huntley D, Molla E, Fernandez-Fuentes MA, et al. Field evaluation of a rapid antigen test (Panbio™ COVID-19 Ag Rapid Test Device) for COVID-19 diagnosis in primary healthcare centres. Clinical Microbiology and Infection, 2020; 27(3):472.e7–472.e10. DOI:10.1016/j.cmi.2020.11.004.

2. Baccani I, Morecchiato F, Chilleri C, Cervini C, Gori E, Matarrese D, et al. Evaluation of Three Immunoassays for the Rapid Detection of SARS-CoV-2 Antigens. Diagnostic Microbiology and Infectious Disease, 2021; 101(2):115434. DOI:10.1016/j.diagmicrobio.2021.115434.

3. Chiu R, Kojima N, Mosley G, Cheng KK, Pereira D, Brobeck M, et al. Evaluation of the INDICAID COVID-19 Rapid Antigen Test in symptomatic populations and asymptomatic community testing. Microbiology Spectrum, 2021; 9(1):e0034221. DOI:10.1128/Spectrum.00342-21.

4. Christensen K, Ren H, Chen S, Cooper C, Young S. Clinical evaluation of BD Veritor™ SARS-CoV-2 and Flu A+B Assay for point-of-care (POC) System. medRxiv [Preprint]; published May 05, 2021. DOI:10.1101/2021.05.04.21256323.

5. Drain PK, Ampajwala M, Chappel C, Gvozden AB, Hoppers M, Wang M, et al. A Rapid, High-Sensitivity SARS-CoV-2 Nucleocapsid Immunoassay to Aid Diagnosis of Acute COVID-19 at the Point of Care: A Clinical Performance Study. Infectious Diseases and Therapy, 2021; 10(2):753–761. DOI:10.1007/s40121-021-00413-x.

6. Faíco-Filho KS, Finamor Júnior FE, Moreira LVL, Lins PRG, Justo AFO, Bellei N. Evaluation of the Panbio™ COVID-19 Ag Rapid Test at an Emergency Room in a Hospital in São Paulo, Brazil. medRxiv [Preprint]; published March 24, 2021. DOI:10.1101/2021.03.15.21253313.

7. Fernandez-Montero A, Argemi J, Rodríguez JA, Ariño AH, Moreno-Galarraga L. Validation of a rapid antigen test as a screening tool for SARS-CoV-2 infection in asymptomatic populations. Sensitivity, specificity and predictive values. EClinicalMedicine, 2021; 37:100954. DOI:10.1016/j.eclinm.2021.100954.

8. Filgueiras P, Corsini C, Almeida NBF, Assis J, Pedrosa ML, de Oliveira A, et al. COVID-19 Rapid Antigen Test at hospital admission associated to the knowledge of individual risk factors allow overcoming the difficulty of managing suspected patients in hospitals COVID-19 Rapid Antigen Test facilitates the management of suspected patients on hospital admission. medRxiv [Preprint]; published January 08, 2021. DOI:10.1101/2021.01.06.21249282.

9. Jung C, Levy C, Varon E, Biscardi S, Batard C, Wollner A, et al. Diagnostic Accuracy of SARS-CoV-2 Antigen Detection Test in Children: A Real-Life Study. Frontiers in Pediatrics, 2021; 9:647274. DOI:10.3389/fped.2021.647274.

10. Korenkov M, Poopalasingam N, Madler M, Vanshylla K, Eggeling R, Wirtz M, et al. Evaluation of a rapid antigen test to detect SARS-CoV-2 infection and identify potentially infectious individuals. Journal of Clinical Microbiology, 2021; 59(9):e0089621. DOI:10.1128/jcm.00896-21.

11. Kurihara Y, Kiyasu Y, Akashi Y, Takeuchi Y, Narahara K, Mori S, et al. The evaluation of a novel digital immunochromatographic assay with silver amplification to detect SARS-CoV-2. Journal of Infection and Chemotherapy, 2021; 27(10):1493-1497. DOI:10.1016/j.jiac.2021.07.006.

12. Merino-Amador P, González-Donapetry P, Domínguez-Fernández M, González-Romo F, Sánchez-Castellano M, Seoane-Estevez A, et al. Clinitest rapid COVID-19 antigen test for the diagnosis of SARS-CoV-2 infection: A multicenter evaluation study. Journal of Clinical Virology, 2021; 143:104961. DOI:10.1016/j.jcv.2021.104961.

13. Merino-Amador P, Guinea J, Muñoz-Gallego I, González-Donapetry P, Galán J-C, Antona N, et al. Multicenter evaluation of the Panbio™ COVID-19 Rapid Antigen-Detection Test for the diagnosis of SARS-CoV-2 infection. Clinical Microbiology and Infection, 2020; 27(5):758-761. DOI:10.1016/j.cmi.2021.02.001.

14. Mertens P, De Vos N, Martiny D, Jassoy C, Mirazimi A, Cuypers L, et al. Development and Potential Usefulness of the COVID-19 Ag Respi-Strip Diagnostic Assay in a Pandemic Context. Frontiers in Medicine, 2020; 7:225. DOI:10.3389/fmed.2020.00225.

15. Micocci M, Buckle P, Hayward G, Allen J, Davies K, Kierkegaard P, et al. Point of Care Testing using rapid automated Antigen Testing for SARS-COV-2 in Care Homes – an exploratory safety, usability and diagnostic agreement evaluation. medRxiv [Preprint]; published April 26, 2021. DOI:10.1101/2021.04.22.21255948.

16. Nordgren J, Sharma S, Olsson H, Jämtberg M, Falkeborn T, Svensson L, et al. SARS-CoV-2 rapid antigen test: High sensitivity to detect infectious virus. Journal of Clinical Virology, 2021; 140:104846. DOI:10.1016/j.jcv.2021.104846.

17. Onsongo SN, Otieno K, van Duijn S, Adams E, Omollo M, Odero IA, et al. Field performance of NowCheck rapid antigen test for SARS-CoV-2 in Kisumu County, western Kenya. medRxiv [Preprint]; published August 13, 2021. DOI:10.1101/2021.08.12.21261462.

18. Orsi A, Pennati BM, Bruzzone B, Ricucci V, Ferone D, Barbera P, et al. On-field evaluation of a ultra-rapid fluorescence immunoassay as a frontline test for SARS-COV-2 diagnostic. Journal of Virological Methods, 2021; 295:114201. DOI:10.1016/j.jviromet.2021.114201.

19. Osmanodja B, Budde K, Zickler D, Naik MG, Hofmann J, Gertler M, et al. Accuracy of a Novel SARS-CoV-2 Antigen-Detecting Rapid Diagnostic Test from Standardized Self-Collected Anterior Nasal Swabs. Journal of Clinical Medicine, 2021; 10(10). DOI:10.3390/jcm10102099.

20. Osterman A, Baldauf HM, Eletreby M, Wettengel JM, Afridi SQ, Fuchs T, et al. Evaluation of two rapid antigen tests to detect SARS-CoV-2 in a hospital setting. Medical Microbiology and Immunology, 2021; 210(1):65-72. DOI:10.1007/s00430-020-00698-8.

21. Pérez-García F, Romanyk J, Gómez-Herruz P, Arroyo T, Pérez-Tanoira R, Linares M, et al. Diagnostic performance of CerTest and Panbio antigen rapid diagnostic tests to diagnose SARS-CoV-2 infection. Journal of Clinical Virology, 2021; 137:104781. DOI:10.1016/j.jcv.2021.104781.

22. Pilarowski G, Lebel P, Sunshine S, Liu J, Crawford E, Marquez C, et al. Performance characteristics of a rapid SARS-CoV-2 antigen detection assay at a public plaza testing site in San Francisco. The Journal of Infectious Diseases, 2020; 223(7):1139-1144. DOI:10.1101/2020.11.02.20223891.

23. Pollock NR, Tran K, Jacobs JR, Cranston AE, Smith S, O'Kane CY, et al. Performance and Operational Evaluation of the Access Bio CareStart Rapid Antigen Test in a High-Throughput Drive-Through Community Testing Site in Massachusetts. Open Forum Infectious Diseases, 2021; 8(7):ofab243. DOI:10.1093/ofid/ofab243.

24. Scohy A, Anantharajah A, Bodeus M, Kabamba-Mukadi B, Verroken A, Rodriguez-Villalobos H. Low performance of rapid antigen detection test as frontline testing for COVID-19 diagnosis. Journal of Clinical Virology, 2020; 129:104455. DOI:10.1016/j.jcv.2020.104455.

25. Suzuki H, Akashi Y, Ueda A, Kiyasu Y, Takeuchi Y, Maehara Y, et al. Diagnostic performance of a novel digital immunoassay (RapidTesta SARS-CoV-2): a prospective observational study with 1,127 nasopharyngeal samples. medRxiv [Preprint]; published August 04, 2021. DOI:10.1101/2021.07.26.21261162.

26. Takeuchi Y, Akashi Y, Kato D, Kuwahara M, Muramatsu S, Ueda A, et al. The evaluation of a newly developed antigen test (QuickNavi™-COVID19 Ag) for SARS-CoV-2: A prospective observational study in Japan. Journal of Infection and Chemotherapy, 2021; 27(6):890-894. DOI:10.1016/j.jiac.2021.02.029.

27. Takeuchi Y, Akashi Y, Kato D, Kuwahara M, Muramatsu S, Ueda A, et al. Diagnostic performance and characteristics of anterior nasal collection for the SARS-CoV-2 antigen test: a prospective study. Scientific Reports, 2021; 11(1):10519. DOI:10.1038/s41598-021-90026-8.

28. Thell R, Kallab V, Weinhappel W, Mueckstein W, Heschl L, Heschl M, et al. Evaluation of a novel, rapid antigen detection test for the diagnosis of SARS-CoV-2. medRxiv [Preprint]; published April 22, 2021. DOI:10.1101/2021.04.22.21255637.

29. Toptan T, Eckermann L, Pfeiffer A, Hoehl S, Ciesek S, Drosten C, et al. Evaluation of a SARS-CoV-2 rapid antigen test: potential to help reduce community spread? Journal of Clinical Virology, 2020; 135:104713. DOI:10.1016/j.jcv.2020.104713.

30. Torres I, Poujois S, Albert E, Álvarez G, Colomina J, Navarro D. Point-of-care evaluation of a rapid antigen test (CLINITEST) Rapid COVID-19 Antigen Test) for diagnosis of SARS-CoV-2 infection in symptomatic and asymptomatic individuals. Journal of Infection, 2021; 82(5):e11–e12. DOI:10.1016/j.jinf.2021.02.010.

31. Torres I, Poujois S, Albert E, Colomina J, Navarro D. Real-life evaluation of a rapid antigen test (Panbio COVID-19 Ag Rapid Test Device) for SARS-CoV-2 detection in asymptomatic close contacts of COVID-19 patients. Clinical Microbiology and Infection, 2020; 27(4):636.E1-636.E4. DOI:10.1016/j.cmi.2020.12.022.

32. Yin N, Debuysschere C, Decroly M, Bouazza FZ, Collot V, Martin C, et al. SARS-CoV-2 Diagnostic Tests: Algorithm and Field Evaluation From the Near Patient Testing to the Automated Diagnostic Platform. Frontiers in Medicine, 2021; 8:380. DOI:10.3389/fmed.2021.650581.

33. Young S, Taylor SN, Cammarata CL, Varnado KG, Roger-Dalbert C, Montano A, et al. Clinical evaluation of BD Veritor SARS-CoV-2 point-of-care test performance compared to PCR-based testing and versus the Sofia 2 SARS Antigen point-of-care test. Journal of Clinical Microbiology, 2020; 59(1). DOI:10.1128/jcm.02338-20.

34. Akashi Y, Kiyasu Y, Takeuchi Y, Kato D, Kuwahara M, Muramatsu S, et al. Evaluation and clinical implications of the time to a positive results of antigen testing for SARS-CoV-2. medRxiv [Preprint]; published June 13, 2021. DOI:10.1101/2021.06.09.21258157.

35. Blairon L, Cupaiolo R, Thomas I, Piteüs S, Wilmet A, Beukinga I, et al. Efficacy comparison of three rapid antigen tests for SARS-CoV-2 and how viral load impact their performance. Journal of Medical Virology, 2021; 93:5783-5788. DOI:10.1002/jmv.27108.

36. Bouassa MRS, Veyer D, Péré H, Bélec L. Analytical performances of the point-of-care SIENNA™ COVID-19 Antigen Rapid Test for the detection of SARS-CoV-2 nucleocapsid protein in nasopharyngeal swabs: A prospective evaluation during the COVID-19 second wave in France. International Journal of Infectious Diseases, 2021; 106:8-12. DOI:10.1016/j.ijid.2021.03.051.

37. Karon BS, Donato L, Bridgeman AR, Blommel JH, Kipp B, Maus A, et al. Analytical sensitivity and specificity of four point of care rapid antigen diagnostic tests for SARS-CoV-2 using real-time quantitative PCR, quantitative droplet digital PCR, and a mass spectrometric antigen assay as comparator methods. Clinical Chemistry, 2021; hvab138. DOI:10.1093/clinchem/hvab138.

38. Kim D, Lee J, Bal J, Seo SK, Chong CK, Lee JH, et al. Development and Clinical Evaluation of an Immunochromatography-Based Rapid Antigen Test (GenBody (TM) COVAG025) for COVID-19 Diagnosis. Viruses-Basel, 2021; 13(5). DOI:10.3390/v13050796.

39. Kim HW, Park M, Lee JH. Clinical Evaluation of the Rapid STANDARD Q COVID-19 Ag Test for the Screening of Severe Acute Respiratory Syndrome Coronavirus 2. Annals of Laboratory Medicine, 2022; 42(1):100-104. DOI:10.3343/alm.2022.42.1.100.

40. Pekosz A, Cooper C, Parvu V, Li M, Andrews J, Manabe YCC, et al. Antigen-based testing but not real-time PCR correlates with SARS-CoV-2 virus culture. Clinical Infectious Diseases, 2020; ciaa1706. DOI:10.1093/cid/ciaa1706.

41. Pickering S, Batra R, Merrick B, Snell LB, Nebbia G, Douthwaite S, et al. Comparative performance of SARS-CoV-2 lateral flow antigen tests and association with detection of infectious virus in clinical specimens: a single-centre laboratory evaluation study. Lancet Microbe, 2021; 2(9):E461-E471. DOI:10.1016/s2666-5247(21)00143-9.

42. Porte L, Legarraga P, Iruretagoyena M, Vollrath V, Pizarro G, Munita J, et al. Evaluation of two fluorescence immunoassays for the rapid detection of SARS-CoV-2 antigen - new tool to detect infective COVID-19 patients. PeerJ, 2020; 9:e10801. DOI:10.1101/2020.10.04.20206466.

43. Strömer A, Rose R, Schäfer M, Schön F, Vollersen A, Lorentz T, et al. Performance of a Point-of-Care Test for the Rapid Detection of SARS-CoV-2 Antigen. Microorganisms, 2020; 9(1). DOI:10.3390/microorganisms9010058.

44. Takeda Y, Mori M, Omi K. SARS-CoV-2 qRT-PCR Ct value distribution in Japan and possible utility of rapid antigen testing kit. medRxiv [Preprint]; published June 19, 2020. DOI:10.1101/2020.06.16.20131243.

45. Van Honacker E, Van Vaerenbergh K, Boel A, De Beenhouwer H, Leroux-Roels I, Cattoir L. Comparison of five SARS-CoV-2 rapid antigen detection tests in a hospital setting and performance of one antigen assay in routine practice: a useful tool to guide isolation precautions? Journal of Hospital Infection, 2021; 114:144-152. DOI:10.1016/j.jhin.2021.03.021.

46. Weitzel T, Legarraga P, Iruretagoyena M, Pizarro G, Vollrath V, Araos R, et al. Comparative evaluation of four rapid SARS-CoV-2 antigen detection tests using universal transport medium. Travel Medicine and Infectious Disease, 2020; 39:101942. DOI:10.1016/j.tmaid.2020.101942.

47. Bachman CM, Grant BD, Anderson CE, Alonzo LF, Garing S, Byrnes SA, et al. Clinical validation of an open-access SARS-COV-2 antigen detection lateral flow assay, compared to commercially available assays. PLoS ONE, 2021; 16(8):e0256352. DOI:10.1371/journal.pone.0256352.
