# Supplementary material for: Accuracy of rapid point-of-care antigen-based diagnostics for SARS-CoV-2: An updated systematic review and meta-analysis with meta-regression analyzing influencing factors
Source: PLoS Med. 2022 May 26;19(5):e1004011. doi: 10.1371/journal.pmed.1004011 (PMC9187092; doi:10.1371/journal.pmed.1004011)
Supplement: Attachment — Submitted filename: Point-to-point response.docx [file pmed.1004011.s025.docx]

Point-to-point response

Dear Editor and Reviewers,

we are thankful for your thorough review of our manuscript and the opportunity to respond to your comments and suggestions. Please find a point-by-point response in this document as well as the revised changes within the submitted manuscript in track change mode.

Editor:

| Please update the search to the most recent date feasible. | Thank you very much for this suggestion, which we have discussed intensively within our team. Given the complexity of our analysis and the number of different stakeholders involved, it would take again several months to conduct the additional review and analysis. In comparison to our previous analysis, the core results did not change. The additional data from an additional 82 clinical accuracy studies allowed further sub-analysis, to explain heterogeneity in the findings. We do not expect further insights from an additional search, particularly as emerging data suggests that variability of sensitivity between variants is also minimal to absent. Thus, we would prefer to keep the search until August 31, 2021. |
| --- | --- |
| Our academic editor commented: "the variant specific analyses one of the reviewers mentioned may be plausible with alpha, delta, and omicron estimates if up to date data are available (would be interesting to see whether there is effect modification depending on what was the predominant variant at the time of testing)". | We fully agree that this is a very interesting analysis. We added pooled estimates for sensitivity and specificity in the results section (line 435 to 446), described the underlying procedure in the methods (line 223 to 226), and interpreted the results in the discussion (line 605 to 610). |
| Please add a new final sentence to the "Methods and findings" subsection of your abstract, which should begin "Study limitations include ..." or similar and should quote 2-3 of the study's main limitations. | We have included a respective statement in line 61 to 62. |
| In the abstract and throughout the paper, please quote p values alongside 95% CI, where available. | Thank you for pointing out this aspect. However, based on recent guidance (Rothmann Eur J Epidemiology 2016, Amrheim Commentary Nature 2019) and as already done in our previous analysis published with PLOS medicine, we would prefer to avoid using p-values. Nonetheless, if the editors find this to be a requirement, we are of course happy to add p values alongside the 95% CI. |
| After the abstract, please add a new and accessible "Author summary" section in non-identical prose. You may find it helpful to consult one or two recently-published research papers in PLOS Medicine to get a sense of the preferred style. | We have added an “Author summary” after the abstract in line 70 to 99. |
| Please add some additional detail in the Methods section (main text) regarding the methods for extracting studies and how disagreement between researchers was resolved, for example. | We have added respective details in line 180 to 206. |
| At line 162, we note that heterogeneity was estimated "visually". Can statistical analyses be reported? | We thank the editors for pointing out this aspect and fully agree that a thorough assessment of heterogeneity is essential. We have added Cochran’s Q and I2 assessment for all pooled estimates, and described the respective procedure in the methods (line 238 to 245). Nonetheless, we would like to highlight that standard guidance suggests not to assess heterogeneity statistically for bivariate diagnostic meta-analysis, since standardized methods are missing (<https://pubmed.ncbi.nlm.nih.gov/15774235/>; https://pubmed.ncbi.nlm.nih.gov/26772804/). Thus, we would prefer to present the statistical measures for heterogeneity in the supplements (Table S3), but are happy to report in the results section if requested. |
| Please restructure the start of the Discussion section (main text), as the first paragraph should summarize the study's main findings. | We have adjusted the first paragraph of our discussion accordingly (line 569 to 575). |
| Throughout the text, please remove spaces from within reference call-outs (e.g., "... in which they are used [2,3]."). | We have adjusted the reference call-outs accordingly. |
| In the reference list, please convert all italic and boldface text into plain text. | We have adjusted the reference list accordingly. |
| Please list 6 authors names followed by "et al.", where appropriate. | We have adjusted the reference list accordingly. |
| Noting reference 181, please use the journal name abbreviation "PLoS ONE".  Noting reference 222 and others, please ensure that all citations have full access details. | We have adjusted reference 181 (now 183) to “PLoS ONE” and added DOI (or URLs where feasible) to all other references. |

Reviewer #1

**Major points:**

| The only substantive comment regards viral load. As the authors state, and as is well known, viral load ranges widely over the course of infection and among infected individuals. Unfortunately, and to some consternation, even after two-plus years, viral loads are still not reported clinically, as they are for numerous other infections (for example, HIV, HCV), for various reasons, or in studies, where the quantitative measure used is the Ct value. This is problematic, as Ct value ranges can differ from one reference test to another, sometimes markedly. | We highly appreciate the reviewer’s detailed review and thoughtful comments! |
| --- | --- |
| To their credit, the authors of this study are well aware of this issue, discuss it ably, and and use viral loads in the cases where they are available (I feel their discussion in 554-555 is perhaps a bit more defensive-sounding than it needs be: Ct value is essentially the only available quantitation at present, so however imperfect, it is completely reasonable to do; I would be surprised if there were pushback to that). | We have adjusted the respective sentence in the discussion (line 663 to 666), now stating that Ct-value ‘is only an estimate of viral load’, in contrast to the previous version, where we stated that Ct-value would be ‘not equivalent’ to viral load. |
| it would be of great utility to name the specific RT-qPCR assay/platform used in each assay, where available, in a supplemental document. (Apologies if this was done and I missed it; I looked ca. L283-286.) I suspect this would explain some of the outliers in Fig. 4. Ideally, the authors would add platform as a regression variable, but I respect that this might be too much to ask, so would not require  redoing the regression with platform as a condition of publication. The effect of differences in limits of detection of these platforms on sensitivity/specificity/concordance between tests should be mentioned (illustrated well in Fig. 2 of https://pubmed.ncbi.nlm.nih.gov/34076471/ , which should be cited). | We have added the types of RT-PCR used in each data set in the Supplement (Table S2). In addition, we described the three RT-PCR assays used most frequently in the results section (line 359 to 363). In the discussion, we highlighted the effects of differences in limits of detection of theses platforms and cited the mentioned source (line 663 to 666). |
| it would be useful (va. L308-319) to give the reader even a rough sense of what viral loads these Ct value cutoffs most likely correspond to, on average, if possible. If not possible, a sentence in this section declaring that to be the case, explaining that Ct scales vary (their reference 3 is a suitable citation). My hope is the field will at some point move to viral loads, and it would wonderful to "future-proof" this excellent review, simply by adding such a Ct-to-viral-load conversion anchor (even a rough one, for example, plus-or-minus two or three orders of magnitude). | Thank you for this suggestion! We have added a respective statement to the beginning of the Ct-value subgroup results section (line 385 to 387). |

**Minor points:**

| Given that it is known what the predominant strain was in each geography (for example https://github.com/hodcroftlab/covariants), is it not possible to list the likely strain for each study? That would offer the opportunity to regress by strain in the future. | Thank you for this valuable suggestion. We have listed the predominant strain in Table S2 and performed a subgroup analysis per strain (line 435 to 446). Furthermore, we added this approach to our methods section (line 223 to 226), and interpreted the results in the discussion (line 605 to 610). |
| --- | --- |
| L99-110, PRISMA checklist: I missed whether the search was done independently by two individuals. This may be a requirement of MOOSE but not PRISMA, or other journals, but not PRISMA/PLoS Medicine. If it was done, please mention. If it was not, that is also fine. (I am *not* suggesting it be done, if it wasn't, as a condition for publication.) | The search was performed by one person, but checked against an expert assembled list of relevant papers. Also, when manually searching the FIND website, this was done by one author and controlled by a second. We have added this to the methods section (line 137 to 145). |
| L228-230: Were the non-English-language papers included? I assume yes, but please clarify. | We included studies without any language restrictions. We have further specified this in the methods (line 154) and results (line 299 to 300). |
| L151-155: I did not quite follow whether this "relaxing" allowed/resulted in overlap, or just aligned bin edges. Perhaps the authors could clarify. | The relaxing resulted in an overlap within the respective subgroups. We have further specified this in line 222 to 223. |
| L302-303: the 95% CIs overlap. Would the authors comment on the probability that this increase is real? I ask especially since the 95% CIs do not overlap for the specificities, yet the authors do not claim that the specificity was lower when using the IFU (which would not be unexpected, given a lower sensitivity). The comparator adjective should be used similarly: I would suggest either these are both comparable, or the first is comparable (since the 95% CIs overlap) and the second is lower. I would suggest they are both comparable. | Thank you for pointing this out. We apologize that there is a misunderstanding and have clarified the respective paragraph (line 379 to 381). |
| L328-331: It is interesting that saliva swabs showed the lowest sensitivity, since saliva has been shown to be comparable to NP as a specimen type (https://pubmed.ncbi.nlm.nih.gov/34406838/). The 95%CI range for saliva in Fig. 3 raises the same question (5% to 95%!). Was there/was it possible to detect a difference between the swabbed saliva and the whole-mouth saliva samples? | As for swabbed saliva only one and for whole-mouth fluid only three data sets were available, we were not able to present pooled estimates for these subgroups. Nonetheless, we added the respective sensitivity ranges to the results section (line 409 to 412). |
| Fig. 5: Given the narrow range of specificities, might the x-axis be usefully contracted to 0.85 or 0.9-1.0, so that differences might be visualized? | We thank the reviewer for this suggestion and have adjusted the forest plots presented in Fig 3a-f (line 457 to 458) and Fig 5 (line 504) accordingly. |
| L551-552: Would also cite <https://www.medrxiv.org/>  content/10.1101/2021.12.22.21268274v1 | Thank you for suggesting this source. We have added it to the respective section in the discussion (line 661). |

Reviewer 2

| I believe the IFU evaluation is valuable, but think there is likely more that can be done, if the data is available. The value of Ag-RDTs can only be fully realized if they can be performed without aid or observation of health workers. The authors specifically mention on lines 523-524 that future research could evaluate "Ag-RDTs that are self-performed or those that are instrument-based." It would be interesting to know if any of the 194 studies were self-performed and/or self-read, and if further stratified analyses could be done to evaluate if sensitivity is impacted (as compared to health care professional performed and read). This would better clarify the role and impact of Ag-RDTs as a public health tool. | Thank you very much for pointing out this aspect. We have just completed a systematic review on self-sampling and self-testing in respect to accuracy and clinical utility for the WHO guideline review committee and are intending to publish this separately. Given that the WHO guidance was just published, we referred to it in the text (line 629 to 631). |
| --- | --- |
| In the same vein, performance of Ag-RDT may be impacted by circulating variants. Were data available to evaluate this (e.g., for wild type, alpha, delta) within the existing cohorts in the review (understanding this study was done pre-omicron)? From the discussion section, it is clear that most studies were done during wild-type or alpha dominance. Proxies could be used such as dominating variant circulating in the study setting during the study period (which I believe is defensible as study mean age and Ct were used in meta-regression), and would permit comparison. | Thank you very much for this suggestion. As already outlined in our response to the other reviewers’ comments, we have listed the predominant strain in Table S2 and performed a subgroup analysis per strain (line 435 to 446). Furthermore, we added this approach to our methods section (line 223 to 226), and interpreted the results in the discussion (line 605 to 610). |
| With respect to specific brands of Ag-RDT being the best performers, I was left wondering if studies that used these Ag-RDT also contained participants with characteristics that increased sensitivity (e.g., IFU-conforming, symptomatic patients, early in symptoms, lower Ct), which would limit our ability to conclude which test may have highest sensitivity. I think more could be done to contextualize these findings, particularly in the discussion. Forgive me if I missed this information. | We listed and analyzed study design factors that could potentially influence test performance in the Supplement for each data set. We fully recognize that this was not clearly mentioned in our manuscript before and have specified this in the results section (line 525 to 526). |
| What were the specimens collected for RT-PCR for the reference standard across studies? I was unable to find this information, however I may have missed it in the dense supplement. There are differences in sensitivity between specimen types for RT-PCR, so if there were different specimens used for the reference standard across studies this should be clear and evaluated. | Thank you for pointing out that we missed to present this information. We have now included it in the Supplement (Table S2) and also referenced to it in line 362 to 363. |
| I believe there may be typos with respect to number of included studies and data sets throughout the manuscript (e.g., Fig 1 states 194 studies included, line 216 says 174). Please review. | We checked this section again and think this might be a misunderstanding. The 174 in the text (previously line 216) only refer to the number of studies found through the search algorithm. To have the full number of studies included (i.e., 194), one still needs to add the 20 studies found on the FIND website. We fully agree that this was written confusingly and have adjusted the respective section (now line 290 to 292). |

Reviewer #3

**Statistical Review**

| As a non-clinical reader, I would have appreciated an explanation of Ct values, in the sense that a low Ct value indicates a higher viral load, earlier in the paper, or even in the abstract. | Thank you for highlighting that Ct-value was not clearly explained. We have added a respective statement in the introduction (line 119 to 121). Given that the abstract is limited to 500 words, we would prefer to use these words for other aspects, but are happy to also add a statement here if required. |
| --- | --- |
| The statistical methods section of the paper is quite good, but does not mention the use of summary ROC curves, when perhaps it should. Also, line 181 mentions multivariate regression of factors associated with sensitivity; "multivariable" is a more accurate term. | Thank you for pointing out that we did not describe creating ROC curves in the methods. We have added this (line 231 to 233) and the results are described in line 516 to 518 and Supplement S10 Fig.  We have rephrased the mentioned line (line 254) to ‘multivariable’. |
| There is mention of the assessment of publication bias using Deeks' test for funnel plot asymmetry. However, I could not find any funnel plots in the paper or the supplement, nor any results of these tests. Why are these not reported? Also, assessment of publication bias is usually not recommended when the number of studies is small; did the authors have specific criteria in terms of the number of studies, when it came to the assessment of publication bias? | We highly appreciate that you recognized the funnel plots were missing. We have added the assessment of publication bias at the end of the results section (line 553 to 556) and the respective figures in the Supplement (S12 Fig). |
| Most research studies require a justification for the sample size of the study. For meta analyses, this is generally not an issue. However, in this case, since this is a living review, the authors have been able to see the data accumulating over time, so perhaps some sort of sample size justification is warranted. Why do the analysis now? Were there pre-defined criteria that triggered this analysis? | Thank you very much for pointing out that we did not specify this sufficiently in the manuscript. We published our first analysis in Feb 2021 (with literature until Dec 15, 2021) without a predefined sample size, but with the aim to inform public health decision makers with the latest results as soon as possible. Based on peer-review, we updated this analysis in June 2021 (with ca. 4 further months of literature). For the present analysis, we included literature for another 4 months. We have included this description in the introduction (line 107 to 112). We would prefer to explain this in the introduction and not in the methods section, as the frequency of our updates arose more from the needs of decision makers than from pre-defined targets. If the editors and reviewers find the methods section more appropriate for this explanation, we are happy to adjust the manuscript. |
| 3 sensitivity analyses are reported. What is the rationale for doing these as sensitivity analyses (i.e. repeating the analysis after excluding a group of studies) rather than as subgroup analyses (i.e. estimating the difference in sensitivity and specificity estimates obtained from studies of different types)? | We only performed subgroup analysis on variables that we expected to directly influence test performance. However, for the variables included in the sensitivity analysis, we view them as confounding factors that should not have a true effect on test performance, but might still impair our analysis. |
| In Figures 3 and 5, it is noticeable that the pooled specificity estimates are all very high. So high, in fact, that it is very difficult to see what is going on, as all the points and confidence intervals are bunched up to the right of the figure. I can understand that there is a need to keep the axes the same, for comparability, but would it help to truncate the x-axis in these figures, e.g. with a lower range of 90%? A lower limit can be used in the supplement, but for the main body of the paper, a higher value perhaps makes sense. | We highly appreciate the reviewer’s comment. As already described above, we have adjusted Fig 3a-f (line 457 to 458) and Fig 5 (line 504) accordingly. |

Reviewer 4

| I've read the present study with great interest. Following a living review of POC antigen-based test for SARS-CoV-2 (updated until August 2021), Authors were able to summarise available evidence on sensitivity and specificity of such instruments. Because of the reduced turnaround time, as well as the option to perform such tests in settings other than hospitals and medical laboratories, POC antigen-based test represent a valuable asset in in our global efforts against SARS-CoV-2 pandemic. Unfortunately, as previously stressed by several studies, the actual sensitivity of these POC tests may be quite inappropriate to fulfil the target of promptly and accurately identify incident cases of SARS-CoV-2 infections. With a sensitivity of around 70%, a large share of cases may fail to be diagnosed, particularly in cases with a low to moderate replication of the pathogen, and in early stages of infections.  Authors, through an appropriate study design and an accurate and diligent application of a proper methodology were able to provide a valuable piece of information for all professionals interested in this specific topic.  Briefly, I've neither requests or recommendations for improving this paper, whose acceptance "as it is" I strongly recommend. | We are very thankful for the reviewer’s thorough evaluation of our manuscript and are very pleased about the recommendation for acceptance! |
| --- | --- |
