# Supplementary material for: Accuracy of rapid point-of-care antigen-based diagnostics for SARS-CoV-2: An updated systematic review and meta-analysis with meta-regression analyzing influencing factors
Source: PLoS Med. 2022 May 26;19(5):e1004011. doi: 10.1371/journal.pmed.1004011 (PMC9187092; doi:10.1371/journal.pmed.1004011)
Supplement: Attachment — Submitted filename: Point-by-point response.docx [file pmed.1004011.s026.docx]

Dear Editor and Reviewers,

we are very thankful for your positive feedback and further comments on our manuscript to enhance the quality of the presented work. We appreciate your trust in our work. You can find a point-by-point response within this letter, as well as the revised changes in the track changes and clean-version manuscript submitted.

Editor

| Our academic editor requests that you state in the paper, as a limitation, perhaps, that the absence of literature post-August 2021 means that information on testing for the Delta and Omicron variants, for example, is lacking. | Thank you very much for this suggestion. We’ve added a respective limitation in line 674 to 676. |
| --- | --- |
| At line 81, please make that "... proved to be" or similar. | We’ve adjusted line 81 accordingly. |
| At line 649, we suggest "... enabled an improved analysis". | We’ve adjusted the mentioned sentence (now line 651) accordingly. |
| Please add an institutional author name to references 1 & 3, and any others. | Thank you for highlighting this. We’ve updated the respective references. |
| Please spell out the group author name in reference 10 and any other relevant references. | We’ve updated the respective source and checked all others (e.g., also adjusted reference 15). |

Reviewer #1

| L385: Would suggest the following change (additions in ALL CAPS): "Can be assumed to be" --> "AS A POINT OF REFERENCE, we assume AS A MEDIAN CONVERSION that Ct value of 25 corresponds to a viral load of 1.5 * 10^6 RNA copies per milliliter OF TRANSPORT MEDIA, but [rest of sentence and refs are fine]". Gives a better idea of why the assumption was made. | Thank you for your suggestion. We have changed the sentence accordingly. Please find the revised sentence at line 384 to 387 |
| --- | --- |
| L222-223: please cite Covariants.org as follows, per the author's recommendation (https://covariants.org/faq#how-should-i-cite-or-acknowledge-this-work): Emma B. Hodcroft. 2021. "CoVariants: SARS-CoV-2 Mutations and Variants of Interest." https://covariants.org/. This will help the author rationalize to funding agencies the value of this work, keeping it available to the scientific community going forward. | Thank you for pointing this out. We have changed the citation respectively (line 223 and reference 16). |
| L662 (or possibly L175-177): It is worth noting explicitly that RT-PCR tests are generally much more sensitive than antigen tests, which fits nicely with the better correlation at lower Ct values (most COVID-19-experienced readers will know this, but safer to not assume that future/all readers will). Please cite <https://academic.oup.com/cid/>  article/73/9/e3042/6127024 as to the importance of LoD. | We agree and have added a respective statement to the limitation of using RT-PCR as a reference standard and have cited the mentioned source (line 661). |
| Table S2: Would add a column for the limit of detection of the comparator and a column for the limit of detection of the Ag-RDT (I went through all the supplements; apologies if I missed this). These are also usefully mentioned where appropriate in L360-362. | We agree with the reviewer that understanding the limit of detection (LOD) of tests is of high importance. Ideally, the LOD as assessed in analytical accuracy studies independent of the test manufacturers would be reported. However, in our review we focus on the clinical accuracy and originally did not extract this data. Nonetheless, where data was publicly available from the test manufacturers, we have noted the LOD as given by the test manufacturer for each Ag-RDT (next to the Ag-RDT’s name) and type of RT-PCR (column ‘Type of RT-PCR used’, directly behind the RT-PCR’s name) listed in Table S2. Still, given that this is data only from test manufacturers, we would prefer to present these values only in the Supplements, but have referred to them in line 362. |

Reviewer #2

| I'd like to thank the authors for thoroughly revising their manuscript based on the editor and reviewer comments. My only remaining comment is for the authors to potentially re-consider how they speak about overlapping confidence intervals - which are not a perfect method to state two measures are not significantly different (see: <https://www.tandfonline.com/doi/abs/>  10.1198/000313001317097960 and https://cscu.cornell.edu/wp-content/uploads/73_ci.pdf). The authors can maintain their messaging even with these statements on overlapping CIs removed. Currently, there is an implication that differences in sensitivity do not exist between tests since confidence intervals overlap - though I think the authors intend to say there is heterogeneity. | Thank you very much for reviewing our manuscript again and suggesting to adjust our reasoning in regards to the confidence intervals. We have adjusted the respective statement in line 606 to 609. |
| --- | --- |

Reviewer #3

| I thank the authors for their consideration of my previous comments. I am happy with the responses, and have no further comments to make. | We highly appreciated your comments and are very pleased that you consider our adjustments to be sufficient. |
| --- | --- |
